# Supplementary material for: The work of farmers in short food supply chains: Systematic literature review and research agenda
Source: PLoS One. 2025 Jun 4;20(6):e0314175. doi: 10.1371/journal.pone.0314175 (PMC12136416; doi:10.1371/journal.pone.0314175)
Supplement: S1 Document — The work of farmers marketing via Short Food Supply Chains: Protocol for a Systematic Literature Review. 2023. doi.org/10.17180/NWMX-3X92. (DOCX) [file pone.0314175.s001.docx]

The work of farmers marketing via
Short Food Supply Chains

Protocol for a Systematic Literature Review

Philippine Dupé^1,2^, Benoît Dedieu^2^, Pierre Gasselin^1^, Guillaume Ollivier^3^October 2023

1. Selmet, Univ. Montpellier, CIRAD, INRAE, Institut Agro, Montpellier, France.
2. Innovation, Univ. Montpellier, CIRAD, INRAE, Institut Agro, Montpellier, France.
3. UR Ecodéveloppement, INRAE, Avignon, France.

DOI : **[10.17180/NWMX-3X92](https://dx.doi.org/10.17180/NWMX-3X92)**

**Summary**

Over the last 20 years or so, farmers the world over have been expressing renewed interest in Short Food Supply Chains (SFSCs). Bringing consumers and producers closer together, these marketing channels are being promoted as a means of recovering added value more effectively. They are seen as part of the solution to the problems associated with long supply chains. However, marketing via SFSCs means that farmers have to take on new tasks that can disrupt their existing work routines. This protocol for a Systematic Literature Review lays out in detail our method to take stock of existing scientific knowledge about the work of farmers marketing their products via SFSCs. After confirming the innovative nature of our approach, we identified several approaches for understanding the concepts of work of farmers and SFSCs. On this basis, we formulated a query that we will use to search two international databases: Web of Science and Scopus. The articles constituting the final corpus will be selected according to detailed eligibility criteria, following a systematic approach. The ‘Rayyan’ selection assistance application will be used to facilitate this selection stage, and a risk of bias assessment will be undertaken. Finally, we present in detail the analysis approach we will use for the qualitative analysis.

Key words**:**  Agriculture, Short Food Supply Chains, Work, Workers

**Contents**

[**Summary** 1](#_Toc159661914)

[**1** **Thematic introduction** 3](#_Toc159661915)

[**2** **Method: a systematic literature review** 3](#_Toc159661916)

[**2.1** **From the research question to formulating a query** 4](#_Toc159661917)

[**2.1.1** **Construction of the thesaurus from the research question** 4](#_Toc159661918)

[**2.1.2** **Identification: information sources** 6](#_Toc159661919)

[**2.1.3** **Search strategy: the query** 7](#_Toc159661920)

[**2.2** **From the results of the query to the development of a corpus liable to answer the research question** 7](#_Toc159661921)

[**2.2.1** **Eligibility criteria** 7](#_Toc159661922)

[**2.2.2** **Screening according to eligibility criteria** 8](#_Toc159661923)

[**2.2.3** **Selection process according to eligibility criteria** 8](#_Toc159661924)

[**2.3** **Building a complementary corpus using the ‘citation chasing’ method** 8](#_Toc159661925)

[**2.4** **Data analysis** 9](#_Toc159661926)

[**2.4.1** **Types of data collected as output** 9](#_Toc159661927)

[**2.4.2** **Risk of bias assessment in the case of meta-analysis of quantitative data** 9](#_Toc159661928)

[**2.4.3** **Qualitative analysis** 10](#_Toc159661929)

[**2.5** **Bibliographical context** 10](#_Toc159661930)

[**2.5.1** **Preliminary searches** 10](#_Toc159661931)

[**2.5.2** **Corpus of publications used to test the query** 11](#_Toc159661932)

[**3** **Appendices** 12](#_Toc159661933)

[**3.1** **Appendix 1: Prisma diagram of the authors’ contributions, inspired by Martin *et al.* (2022)** 12](#_Toc159661934)

[**3.2** **Appendix 2: List of publications retrieved from our query to ensure that an existing review similar to the one we propose to carry out does not already exist** 13](#_Toc159661935)

[**3.3** **Appendix 3: Examples of definitions of SFSCs** 14](#_Toc159661936)

[**3.4** **Appendix 4: References** 16](#_Toc159661937)

1. **Thematic introduction**

There has been a revival since the early 2000s of Short Food Supply Chains (SFSCs) in many countries (Kneafsey et al. 2013; Heinisch et al. 2014a; Philipon et al. 2017) in a variety of contexts (Deverre and Lamine 2010). Some SFSCs have existed for a long time (open-air markets, farm-gate sales), while others are more recent (teikei^^[[1]](#footnote-1)^^, Community Supported Agriculture (CSA)^^[[2]](#footnote-2)^^, farmers’ drives, online sales, public purchases of local produce for catering, etc.). Although in a number of countries the volumes of production sold through these channels remain small compared with those through long channels (Augère-Granier 2016; Martinez and Park 2021), these SFSCs are now finding favour with a significant proportion of farmers (*Ibid.*).

Encouraging a reconnection between producers and consumers, leading to an improved distribution of added value, these SFSCs are often promoted as part of the solution to certain problems of current agricultural and food models (Kneafsey et al. 2013; United Nations Industrial Development Organization 2020; Enthoven and Van den Broeck 2021). They are therefore gradually gaining institutional recognition and dedicated support (Heinisch et al. 2014b; Augère-Granier 2016; Martinez and Park 2021).

However, farmers who want to sell their products through SFSCs have to take on additional tasks concerning marketing or even the processing of their products (Chiffoleau et al. 2013), thus adding to their already busy work schedules (Philipon et al. 2017). Furthermore, farmers have to develop new skills corresponding to these tasks within work collectives (Chiffoleau et al. 2013). While a number of literature reviews on SFSCs have focused on the sustainability of these marketing channels, the issues of income and job creation in agricultural areas, and the producer-consumer relationship in short distribution channels (Michel-Villarreal et al. 2019; Chiffoleau and Dourian 2020; Evola et al. 2022), none of them has addressed the issue of the work of farmers in SFSCs. This issue is however clearly identified as a challenge in the literature (Bayir et al. 2022), and it is the one we focused on. Through our review, we will identify and characterise the knowledge available in the scientific literature on agricultural work in SFSCs.

1. **Method: a systematic literature review**

Since the systematic literature review method as described by Petticrew and Roberts (2006) makes it possible to find the answer to a specific question and to account for a diversity of phenomena at work and their evolution (Petticrew and Roberts 2006; Munn et al. 2018), we have chosen to use it. This method of conducting a literature review is transparent and reproducible (Munn et al. 2018).

We use the methodological checklist developed by PRISMA (Preferred Reporting Items for Systematic Reviews and Meta-Analysis) to specify our approach (Page et al. 2021). We first present in detail the construction of our thesaurus based on our research question. We then introduce the databases that we intend to query, as well as the queries we have formulated to identify existing articles that are liable to provide answers to our research question. Finally, we describe the publication selection process and the risks of bias.

- 1. **From the research question to formulating a query**

In this sub-section, we explain in detail our choices regarding the construction of our query, first concerning the construction of our thesaurus, then those concerning the databases we have chosen to query and finally the queries themselves.

- - 1. **Construction of the thesaurus from the research question**
       1. **Work in agriculture: thesaurus and analytical frameworks**

The concept of ‘work’ is used by various disciplines, each mobilising specific analytical frameworks (Malanski et al. 2019); it is thus a particularly polysemous term (Dujarier 2021). We hypothesise that marketing agricultural products through SFSCs is likely to impact many dimensions of agricultural work, which compels us to consider several facets of this concept in our review.

- Some authors focus on **elements of farm structures or the agricultural fabric** around SFSCs (Aubert 2013; Benedek et al. 2018; Morsel and Garambois 2022) or on the **agricultural labour market** as pertaining to SFSCs (Mundler and Jean-Gagnon 2020 ; Schreiber et al. 2023).
- Other authors examine the **technical and economic performance of labour** on these farms, using indicators such as labour productivity, farm income and added value (Galt 2013; Mundler and Jean-Gagnon 2020).
- Some researchers have studied the **organisation of work** in SFSCs at different scales, either by considering the spatio-temporal organisation of tasks between workers at various scales (Aubry et al. 2011; Paranthoën and Wavresky 2021; Morsel and Garambois 2022) or by focusing in particular on the social relations of production (Weiler et al. 2016).
- Some studies have explored agricultural **working conditions** in SFSCs, sometimes in terms of physical or mental hardship, or in terms of job satisfaction or meaning (Dupré et al. 2017; Azima and Mundler 2022).
- Finally, others examine the role played by SFSCs in forming the identity of **professions**, along with their norms and values, their practices and their skills (Le Bahers et al. 2016; Chiffoleau 2017).

The aim is therefore to select terms that reflect these different dimensions, while eliminating as much noise as possible. As Martin *et al.* (2022) mention:

- ‘**Work**’ is the term most often used by the research community in relation to work in agriculture (Malanski et al. 2019; Hostiou et al. 2020; Duval et al. 2021; Barisaux et al. 2022).
- ‘**Labour**’ or ‘**labor**’ is widely used in economics and sociology to examine the social relations of production or to study labour as a factor of production in order to evaluate its performance (‘labour productivity’).
- ‘**Job**’ or ‘**employment**’ refer to a macro-economic vision of work.
- ‘**Task**’ is used in ergonomics and in the study of the organisation of work.
- ‘**Occupation**’ relates to identity, profession, and social status.

All these terms are therefore included in our query. Following the example of Malanski *et al.* (2019) and Martin *et al.* (2022), we exclude the terms ‘activity’ and ‘practice’, even though they are frequently used in ergonomics, because they add too much noise to the query.

- - - 1. **Agriculture: thesaurus**

We use the general terms ‘agriculture’, ‘farm’ and ‘rural’ for this thesaurus. These terms refer to different scales of analysis at which agricultural work seems likely to be impacted by SFSCs, namely: the scale of the agricultural worker, the scale of the agricultural production system (Dedieu 2019), the scale of agricultural territories (labour market, territorial organisation of work), and the scale of agricultural models (Gasselin et al. 2023). Even though all types of production can be marketed through SFSCs, we have chosen not to classify in detail the different types of production in the thesaurus in order to avoid a lack of exhaustiveness.

- - - 1. **SFSCs: thesaurus**

Although SFSCs have been around for a long time, the phenomenon has only recently been conceptualised by the research community and incorporated in public policy (Kneafsey et al. 2013). Building a thesaurus for SFSCs therefore involves identifying the various concepts used in the literature to refer to them. Several criteria have been used in the literature to establish typologies of marketing channels. Among them, some authors distinguish the different types of marketing channels on the basis of geographical or organisational proximities as defined by Kebir and Torre (2013) or Bouba-Olga *et al.* (2015). Other authors use instead elements of breaks with the conventional model to distinguish different types of short circuits. Still others classify them according to how much they respect the three pillars of sustainable development.

- - - - 1. Short Food Supply Chains (SFSCs)

Some 20 years after the phenomenon was first conceptualised, most notably by Marsden *et al.* (2000), the concept of the ‘short food supply chain’ continues to be defined differently by authors (Kneafsey et al. 2013). It sometimes refers to marketing channels that involve a limited number of intermediaries, often no more than one, between the producer and the consumer (MAAF 2009; Bayir et al. 2022). Other definitions add elements of geographical proximity to this criterion (Marsden et al. 2000; Kebir and Torre 2013). With regard to agricultural work, we hypothesise that reducing the number of intermediaries or reducing the physical distances between producers and consumers impacts the work of farmers involved in SFSCs. For this reason, we retain a broad and inclusive definition of SFSCs, following the example of Paciarotti and Torregiani (2021) in their review.

- - - - 1. Concepts peripheral to SFSCs

The scientific literature on SFSCs often uses similar concepts such as ‘Local Food Systems’, ‘Alternative Food Networks’ and ‘Sustainable Food Supply Chains’.

- ‘Local Food Systems’ (LFS) are generally defined on the basis of several registers of proximity (geographical, relational, organisational, social, and of values) with an emphasis on geographical proximity (Enthoven and Van den Broeck 2021). SFSCs are considered by many authors to be one of the main forms of marketing channels falling into this category (*Ibid*.).
- ‘Alternative Food Networks’ (AFN) tend to designate marketing channels ‘as somehow oppositional to “conventional” food systems’, in particular because of the limited number of intermediaries and the local origin of products (Michel-Villarreal et al. 2019; Renkema and Hilletofth 2022).
- ‘Sustainable food supply chains’ refer to channels that respect the pillars of sustainable development (Kumar et al. 2022 ; Chiffoleau and Dourian 2020). As such, some SFSCs are included in this broader concept (Malak-Rawlikowska et al. 2019). This concept, in particular because it takes into account a pillar of social sustainability, makes it possible to explore issues such as job creation at a territorial scale, working conditions, remuneration and the sharing of added value, which are fully within the scope of our interest.

These three concepts and the terms associated with them are therefore included in our thesaurus, in addition to that of SFSC, in order to impart robustness to our review by widening our search field.


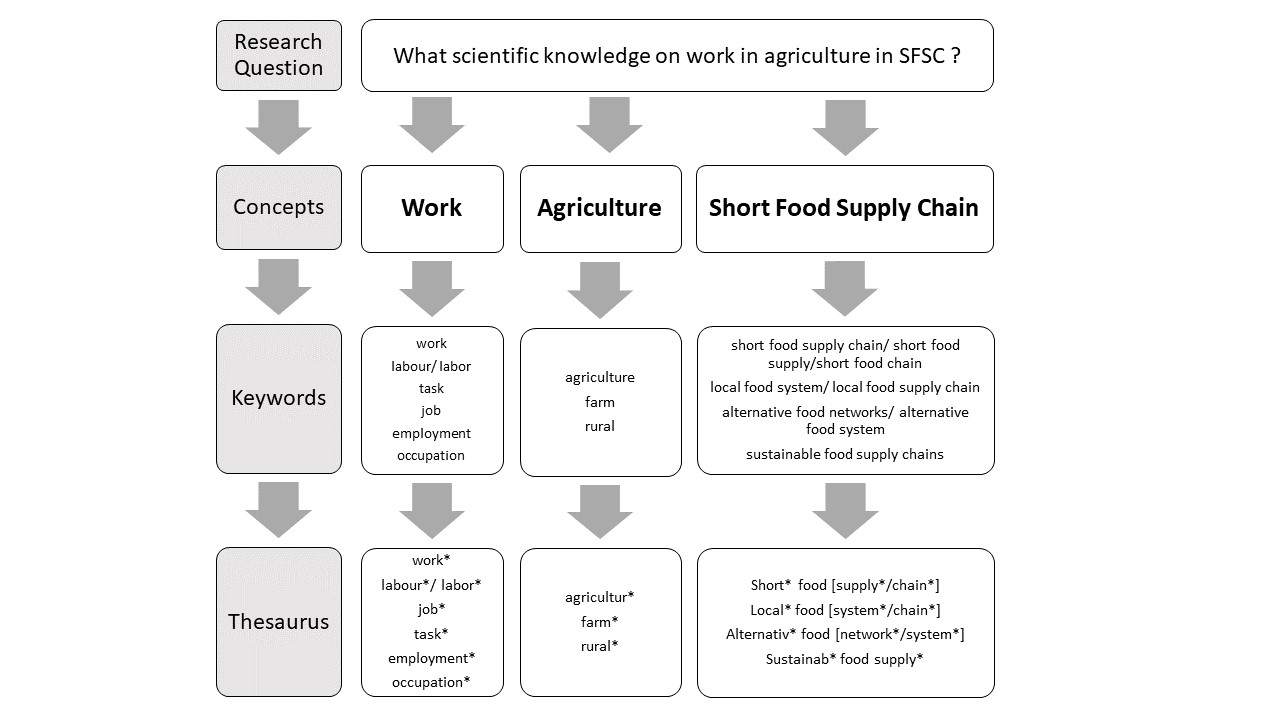


*Figure 1. Summary table of the construction of the thesaurus on the basis of our research question.*

- - 1. **Identification: information sources**

We have chosen to use the Web of Science Core Collection (WoS) and Scopus, the world’s main catalogues of scientific literature. These are the databases preferred by journals on work in agriculture (Malanski et al. 2019; Hostiou et al. 2020; Duval et al. 2021; Martin et al. 2022) as well as by journals focusing on SFSCs (Michel-Villarreal et al. 2019; Paciarotti and Torregiani 2021; Evola et al. 2022; Kumar et al. 2022; Renkema and Hilletofth 2022).

We will allow ourselves to add articles identified as being of interest even if they are not returned by our queries, but which are cited several times by the articles that will be returned by our queries (see section 2.3 on ‘citation chasing’).

- - 1. **Search strategy: the query**

We will include in our review all peer-reviewed articles or book chapters. We will filter the query to only return English-language publications. We will consider papers from all disciplines, methods and countries. Documents will be retrieved for all the years available in the databases.

After preliminary testing of the queries in the two databases, we intend to use the queries listed below.

- - - 1. **Reminder of the thesauri**

W= (work* OR labour* OR labor* OR job* OR task* OR employment* OR occupation*)

A= (agricultur* OR farm* OR rural*)

S = ("short* food supply*" OR "short* food chain*" OR "local* food system*" OR "local* food chain*" OR "alternativ* food network*" OR "alternativ* food system*" OR "sustainab* food supply*")

- - - 1. **Web of Science query**

**Query used** 🡪 **W (Topic) AND A (Topic) AND S (Topic)**

**Language**: English

**Document types**: Article OR Book Chapters OR Review article

- - - 1. **Scopus query**

**Query used** 🡪 **W (TITLE-ABS-KEY) AND A (TITLE-ABS-KEY) AND S (TITLE-ABS-KEY)**

**Language**: English

**Document types**: Article OR Book Chapter OR Review

- 1. **From the results of the query to the development of a corpus liable to answer the research question**

We present in detail below our method for eliminating publications returned by the query but which are unlikely to provide an answer to our research question. First, we will eliminate duplicates that will be returned from both databases. We will then assess the relevance of the articles in terms of the eligibility criteria, first on the basis of titles, abstracts and keywords, and then on the basis of a final reading of the full texts (*Figure 3*).

- - 1. **Eligibility criteria**

To restrict our selection to only those publications that are likely to provide answers to our research question, we have developed eligibility criteria based on the PICO method (Methley et al. 2014).

| PICO Components | Details |
| --- | --- |
| Participants (population) | Farms, farmers and other agricultural workers in all sectors of production |
| Intervention (exposure) | Marketing at least part of the production through SFSCs |
| Comparators (control) | Farms that are not, not yet or no longer marketing through SFSCs |
| Outcomes | - **Structural elements of work in SFSCs (Structure): categories of workers, farm structures and associated labour market:** type of workers involved in SFSCs-related agricultural work, production systems and activity systems of the farms concerned, labour market and employment rate linked to SFSC-related agricultural work at the farm and territorial scales. - **Economic Performance:** main economic indicators pertaining to work performance (productivity, profitability, income, etc.). - **Work Organisation:** Spatio-temporal organisation of tasks between workers and social relations of production at the scale of: - Farms - Collective sales outlets - Agricultural territories - **Working Conditions:** - **Physical and mental hardship** - **Meaning of work:** any form of subjectivity and emotion in the work (stress, satisfaction, recognition, autonomy, sense of coherence, identity, etc.) or system of motivation around this work - **Occupation:** identity of the profession, skills (objectivised, situational) and training, professional norms and values (especially craftsmanship/entrepreneurship), practices, objectives, job solidarity |

*Figure 2. PICO table summarising our eligibility criteria.*

- - 1. **Screening according to eligibility criteria**

After articles will be retrieved from the databases, and duplicates eliminated via Rayyan – a free screening assistance application (Ouzzani et al. 2016) –, two individuals will conduct the screening process. During this pre-selection stage, the publications, abstract titles and key words of each article will be checked against the pre-established eligibility criteria (*Figure 2*) in a double-blind process. Articles on which there may be disagreement will then be discussed between the authors to arrive at a decision (Appendix 1).

- - 1. **Selection process according to eligibility criteria**

We will then assess the relevance of the remaining publications after reading them in full, again in light of the eligibility criteria (*Figure 2*). In this way, we will obtain our final corpus.

- 1. **Building a complementary corpus using the ‘citation chasing’ method**

Citation chasing is a supplementary search technique for complementing a corpus. It checks potentially relevant publications that may have been missed by the lexical search. This technique adds to the methodological rigour of the review.

We will use the ‘citationchaser’ tool (<https://estech.shinyapps.io/citationchaser>) which generates a list of backward and forward citations from a first set of selected papers using the citation database from the lens.org API (Haddaway et al. 2022). Backward citation are references cited by papers in the selected corpus, while forward citations are publications citing papers in this corpus. The tool returns these two lists with the citation counts. We will merge both lists in order to delete duplicates, given that some publications may cite or be cited by the initial corpus. Using DOI, we will remove publications that will already have been screened out, providing a new list of candidate publications.

The publications identified using citation chasing will themselves be checked against the eligibility criteria during (i) a screening phase based on titles, then abstracts and keywords, followed by (ii) an eligibility phase based on a full reading of the remaining publications. This complementary corpus of publications will be added to the list of articles from the queries to form the final corpus for analysis.


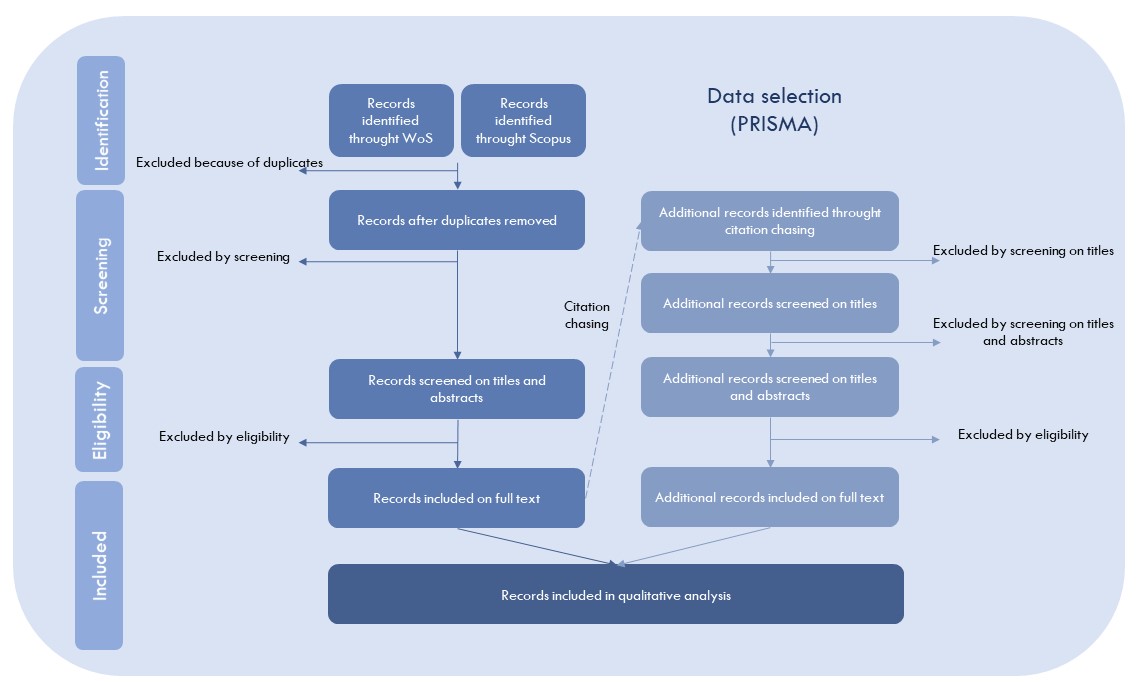


*Figure 3. Summary diagram of the method used to compile the corpus.*

- 1. **Data analysis**
     1. **Types of data collected as output**

Two types of data will be extracted from the selected articles:

- Qualitative data on the organisation of work, the meaning of work and the conception of the profession.
- Conclusions drawn from the analysis of quantitative data. It should be noted that quantitative data will not be subjected to a meta-analysis and will not be included as such in our review, unless we obtain a sufficient quantity and quality of data on a subject.
  - 1. **Risk of bias assessment in the case of meta-analysis of quantitative data**

Should we obtain a sufficient quantity of quantitative and comparable data, we will be able to carry out a meta-analysis. In this case, we will carry out a risk of bias assessment in order to be transparent about the limits of our reasoning. We have identified two major risks of bias.

- One relates to our query, which is inherently incomplete. In order to obtain a humanly processable output corpus, we have had to make choices to limit the noise that would be generated with an overly broad thesaurus.
- The other relates to the possible biases of the publications themselves. Indeed, we believe that any scientific method can be improved. It should be noted that, since we are assessing the risk of bias in the light of our research question, we do not pass judgement as such on the authors’ methodological choices, which were motivated by their own research questions.
  - As for the publications, it may be that the situations presented in the publications selected are inadequately characterised in terms of:
    - Location and contextual information
    - Technical and economic characterisation of the farms concerned
    - Characterisation of the workers involved (farm managers, family labour, farm employees, etc.) and of the tasks they perform (production, processing, marketing, etc.)
    - Characterisation of the SFSCs concerned (number of intermediaries, proximity factors, etc.)
  - It can also be possible that any changes in the various dimensions of the work described are not due to the SFSCs, but to structural changes in the way the farm operates, or to changes in the socio-economic and regulatory context.

Should we decide to carry out such a meta-analysis, we would first specify our criteria for assessing the risk of bias in the light of the nature of the data considered, and would draw up a bias analysis table.

- - 1. **Qualitative analysis**

Five dimensions of work will be considered in our qualitative analysis, corresponding to the Outcomes in our PICO table (*Figure 2*). We will address these five themes with particular attention to :

- The different types of farms (size, main productions, types of work, combinations of products and marketing networks)
- The different SFSC marketing channels
- The different finished products (vegetables, fruit, meat products, dairy products, other)
- The different countries studied.
  1. **Bibliographical context**
     1. **Preliminary searches**

To confirm the relevance of our review project, we carried out a preliminary search on WoS and Scopus to ensure that such a review did not already exist.

- W= (work* OR labour* OR labor* OR job* OR task* OR employment* OR occupation*)
- A= (agricultur* OR farm* OR rural*)
- S = ("short* food supply*" OR "short* food chain*" OR "local* food system*" OR "local* food chain*" OR "alternativ* food network*" OR "alternativ* food system*" OR "sustainab* food supply*")
- R =(review* OR survey* OR bibliometric* OR literature* OR meta-analysis)

**Query used for WoS** 🡪 **W (topic) AND A (topic) AND S (topic) AND R (title)**

**Language**: English

**Document types**: All

**Query date**: 18/10/2023

**Query used for Scopus** 🡪 **W (Article title, Abstract, Keywords) AND A (Article title, Abstract, Keywords) AND S (Article title, Abstract, Keywords) AND R (Article title)**

**Language**: English

**Document types**: All

**Query date**: 18/10/2023

After using Rayyan to eliminate duplicates, none of the resulting 20 publications focused on our topic or used a Systematic Literature Review method.

- - 1. **Corpus of publications used to test the query**

To test the reliability of our query, we have compiled a corpus of publications on the basis of our knowledge of the subject. These publications have to appear in our list of final publications.

| Authors | Title | Journal | Date | DOI |
| --- | --- | --- | --- | --- |
| Azima, S., Mundler, P. | Does Direct Farm Marketing Fulfill Its Promises? Analyzing Job Satisfaction among Direct-Market Farmers in Canada | *Agriculture and Human Values* | 2022 | 10.1007/s10460-021-10289-9. |
| Azima, S., Mundler, P. | The Gendered Motives and Experiences of Canadian Women Farmers in Short Food Supply Chains: Work Satisfaction, Values of Care, and the Potential for Empowerment | *Journal of Rural Studies* | 2022 | 10.1016/j.jrurstud.2022.10.007. |
| Dupré, L.  Lamine, C., Navarrete, M. | Short Food Supply Chains, Long Working Days: Active Work and the Construction of Professional Satisfaction in French Diversified Organic Market Gardening | *Sociologia Ruralis* | 2017 | 10.1111/soru.12178. |
| Galt, R.E. | The Moral Economy Is a Double-edged Sword: Explaining Farmers’ Earnings and Self-exploitation in Community-Supported Agriculture | *Economic Geography* | 2013 | 10.1111/ecge.12015 |
| Morsel, N. and Garambois, N. | Agroecology in the Limousin Mountains: Relocating and Diversifying Food Production to Encourage Employment and Conserve Semi-Natural Spaces. | *Revue de Géographie Alpine* | 2022 | 10.4000/rga.10633 |
| Mundler, P., et Jean-Gagnon, J. | Short Food Supply Chains, Labor Productivity and Fair Earnings: An Impossible Equation? | *Renewable Agriculture and Food Systems* | 2020 | 10.1017/S1742170519000358. |
| Schreiber, K., Soubry, B., Dove-McFalls, C. and MacDonald, G. K. | Untangling the role of social relationships for overcoming challenges in local food systems: a case study of farmers in Québec, Canada. | *Agriculture and Human Values* | 2023 | 10.1007/s10460-022-10343-0 |
| Weiler, A.M.; Otero, G.; Wittman, H. | Rock Stars and Bad Apples: Moral Economies of Alternative Food Networks and Precarious Farm Work Regimes | *Antipode* | 2016 | 10.1111/anti.12221 |

*Figure 4: Corpus of publications identified as being of interest for our research question.*

These publications did indeed all appear in the output of our queries.

1. **Appendices**
   1. **Appendix 1: Prisma diagram of the authors’ contributions, inspired by Martin *et al.* (2022)**


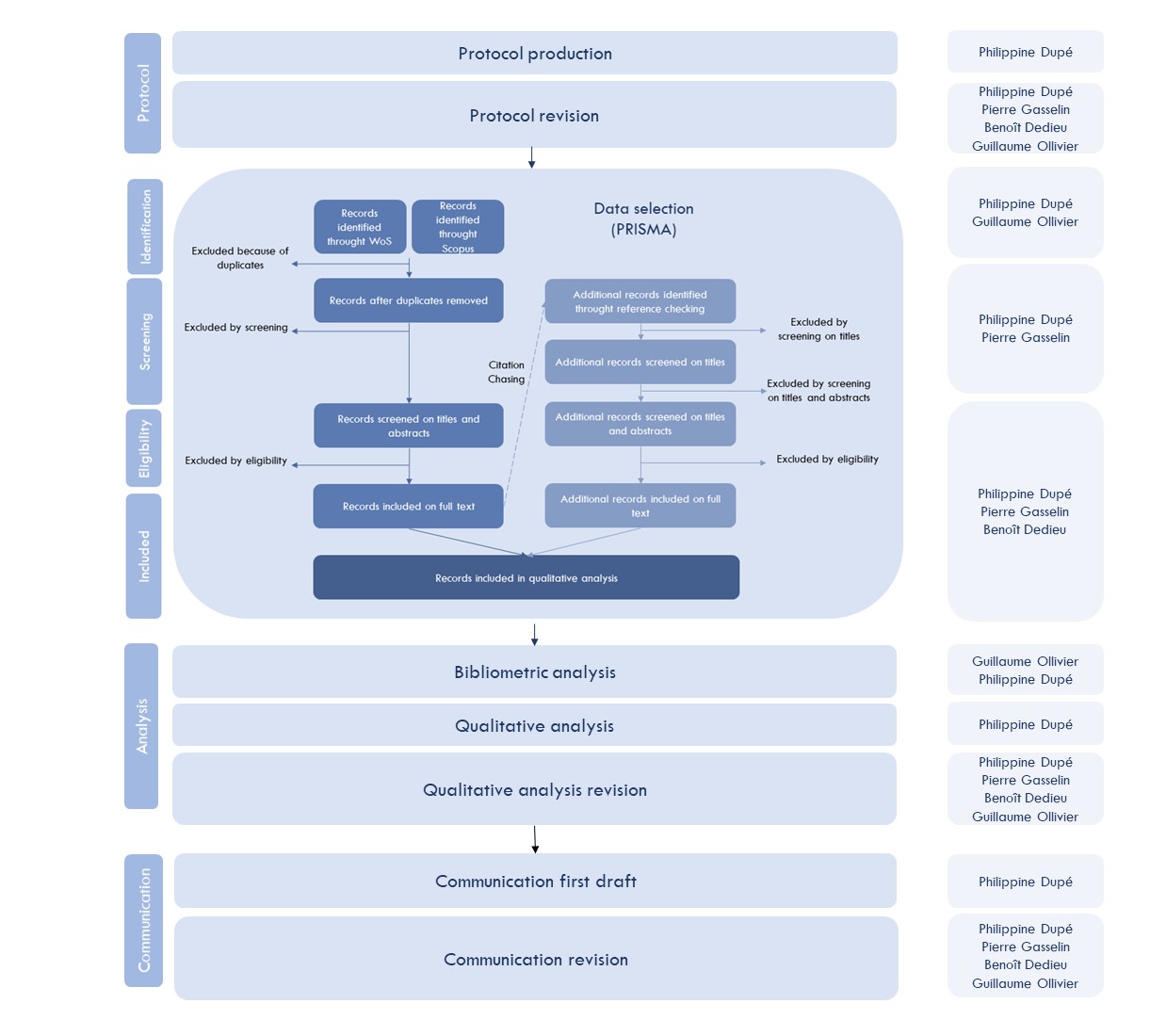


- 1. **Appendix 2: List of publications retrieved from our query to ensure that an existing review similar to the one we propose to carry out does not already exist**

| Authors | Title | Journal | Date | Reason(s) for exclusion |
| --- | --- | --- | --- | --- |
| Akram, H.W., Akhtar, S., Ahmad, A., Anwar, I., Sulaiman, M.A.A.B. | Developing a Conceptual Framework Model for Effective Perishable Food Cold-Supply-Chain Management Based on Structured Literature Review | SUSTAINABILITY | 2023 | Out of scope |
| Egli, L., Rüschhoff, J., Priess, J. | A systematic review of the ecological, social and economic sustainability effects on community-supported agriculture | FRONTIERS IN SUSTAINABLE FOOD SYSTEMS | 2023 | Not focused on work. Only focused on one type of SFSC |
| Sulistyowati, C.A., Afiff, S.A.A. , Baiquni, M., Siscawati, M. | Challenges and potential solutions in developing community supported agriculture: a literature review | AGROECOLOGY AND SUSTAINABLE FOOD SYSTEMS | 2023 | Not focused on work. Only focused on one type of SFSC. Not a systematic literature review |
| Apaliya, M.T., Kwaw, E., Osae, R., Alolga, R.N., Aikins, A.S.S., Otoo, G.S., Kaburi, S.A., Lamptey, F.P., Amo-Broni, C. | THE IMPACT OF COVID-19 ON FOOD SECURITY: GHANA IN REVIEW | JOURNAL OF FOOD TECHNOLOGY RESEARCH | 2022 | Out of scope |
| Csordas, A., Lengyel, P., Fuzesi, I. | Who Prefers Regional Products? A Systematic Literature Review of Consumer Characteristics and Attitudes in Short Food Supply Chains | SUSTAINABILITY | 2022 | Not focused on work |
| Anderson, J. D., Mitchell, J.L., Maples, J.G. | INVITED REVIEW: Lessons from the COVID-19 pandemic for food supply chains | APPLIED ANIMAL SCIENCE | 2021 | Not focused on work. Not a systematic literature review |
| Benedek, Z., FertÃ¶, I., Marreiros, C.G., De Aguiar, P.M., Pocol, C.B., Cechura, L., Poder, A., PÃÃso, P., Bakucs, Z. | Farm diversification as a potential success factor for small-scale farmers constrained by COVID-related lockdown. Contributions from a survey conducted in four European countries during the first wave of COVID-19 | PLOS ONE | 2021 | Not focused on work. Not a systematic literature review |
| Paciarotti, C., Torregiani, F. | The logistics of the short food supply chain: A literature review | SUSTAINABLE PRODUCTION AND CONSUMPTION | 2021 | Not focused on work. Not a systematic literature review |
| Saediman, H., Gafaruddin, A., Hidrawati, H., Salam, I., Ulimaz, A., Sarimustaqiyma Rianse, I., Sarinah, S., Adha Taridala, S.A | The contribution of home food gardening program to household food security in Indonesia: A review | WSEAS TRANSACTIONS ON ENVIRONMENT AND DEVELOPMENT | 2021 | Out of scope |
| Szente, V., Ferto I., Benedek, Z. | Does the purchase of local food contribute to local economy development? A systematic review | TER ES TARSADALOM | 2021 | Not focused on work |
| Panday, U.S., Pratihast, A. K., Aryal, J., Kayastha, R.B. | A Review on Drone-Based Data Solutions for Cereal Crops | DRONES | 2020 | Out of scope |
| Figueroa-Rodriguez, K.A., del Alvarez-Avila, M., Hernandez-Castillo, F., Schwentesius-Rindermann, R., Figueroa-Sandoval, B. | Farmers’ Market Actors, Dynamics, and Attributes: A Bibliometric Study | SUSTAINABILITY | 2019 | Not focused on work. Only focused on one type of SFSC. Not a systematic literature review |
| Francis, C.A., Lieblein, G. | Review and Critique of the TATA-BOX Model - AGROECOLOGICAL TRANSITIONS: From Theory to Practice in Local Participatory Design |  | 2019 | Out of scope |
| Machida, D., Yoshida, T. | Factors that Affect Nonmarket Fruit and Vegetable Receptions: Analyses of Two Cross-Sectional Surveys in Gunma, Japan | AGRICULTURE-BASEL | 2019 | Not a review |
| Aggestam, V., Fleiss, E., Posch, A. | Scaling-up short food supply chains? A survey study on the drivers behind the intention of food producers | JOURNAL OF RURAL STUDIES | 2017 | Not a systematic literature review. Only one dimension of work considered |
| Demartini, E., Gaviglio, A., Pirani, A. | Farmers’ motivation and perceived effects of participating in short food supply chains: evidence from a North Italian survey | AGRICULTURAL ECONOMICS-ZEMEDELSKA EKONOMIKA | 2017 | Not a review |
| Drake, L., Lawson, L.J. | Results of a US and Canada community garden survey: shared challenges in garden management amid diverse geographical and organizational contexts | AGRICULTURE AND HUMAN VALUES | 2015 | Not a review |
| Fabbrizzi, S., Menghini, S., Marinelli, N | The short food supply chain: A concrete example of sustainability. A literature review | RIVISTA DI STUDI SULLA SOSTENIBILITA | 2014 | Not focused on work. Not a systematic literature review |
| Lin, Y., Vogt, R., Larssen, T. | Environmental mercury in China: A review | ENVIRONMENTAL TOXICOLOGY AND CHEMISTRY | 2012 | Out of scope |
| Zasada, I. | Multifunctional peri-urban agriculture – A review of societal demands and the provision of goods and services by farming | LAND USE POLICY | 2011 | Not focused on work. Not a systematic literature review |

- 1. **Appendix 3: Examples of definitions of SFSCs**

| Publication | Proposed definition |
| --- | --- |
| Bayir, B., A. Charles, A. Sekhari, and Y. Ouzrout. 2022. ‘Issues and Challenges in Short Food Supply Chains: A Systematic Literature Review.’ *Sustainability (Switzerland)* 14 (5). https://doi.org/10.3390/su14053029. | *The French Ministry of Agriculture, Food, and Forestry defines SFSCs as the ‘commercialization of agricultural products through direct selling or indirect selling when only one intermediary is involved’ (p. 198). According to this definition, even though the locality of the food and the minimized number of intermediaries make part of ideal-type SFSCs, these distribution channels are not limited to direct sales or local food. Similarly, SFSCs can be limited to organic products, but this is not always necessarily the case (Bayir et al., 2022, p. 1).* |
| Renkema, Marije, and Per Hilletofth. 2022. ‘Intermediate short food supply chains: a systematic review.’ *British Food Journal* 124 (13): 541‑58. https://doi.org/10.1108/BFJ-06-2022-0463. | *A SFSC can be conceived either as a physical distance or as a cognitive distance, based on the number of actors involved in linking production and consumption (Loconto et al., 2018).*  *The main idea of SFSCs is the direct or closest possible relationship between the producer and the consumer, rather than solely an exchange of a product. The relationship involves the construction of knowledge, value and meaning about the product and its origin, production and consumption (Maciejczak, 2014).*  *Marsden et al. (2000) propose three main types:*   - - *Direct-to-consumer SFSCs, where consumers buy a product directly from the producer, allowing for authenticity and trust via personal interactions;*   - *Proximate intermediate SFSCs, where products are sold close to where they are produced and consumers are aware of the ‘local’ nature of goods at retail level; and*   - *Spatially extended intermediate SFSCs, where production and point of sale are not necessarily local, but information about the place of production, as well as the producer, is communicated to consumers. These SFSCs may create sustainability in the food system by their focus on economic sustainability, where the producers get more value for their products, social sustainability, where producers develop socially embedded relationships and environmental sustainability often calculated by shorter food miles and environmentally friendly production methods (Malak-Rawlikowska et al., 2019).*   *Direct-to-consumer relations have previously been regarded as a favourable solution to unsustainable global food supply chains. However, the disadvantages of direct-to-consumer supply chains are many; being in control of sales adds labor and marketing costs, limits scalability and may result in unreliable turnover for producers and limited supply for customers (Cembalo et al., 2015) […] Due to these disadvantages of direct-to-consumer SFSCs, researchers have changed their focus to intermediate SFSCs as a possible solution to create sustainable food systems. In fact, research recognizes an overall increase in sales through all types of SFSCs. However, sales via intermediate SFSCs make out the largest volume sold and market share compared to other direct SFSCs, where the number of producers might be relatively higher, the volume of sold produce and market share is low (Malak-Rawlikowska et al., 2019; Plakias et al., 2020).* |
| Paciarotti, Claudia, and Francesco Torregiani. 2021. ‘The Logistics of the Short Food Supply Chain: A Literature Review.’ *Sustainable Production and Consumption* 26 (April): 428‑42. https://doi.org/10.1016/j.spc.2020.10.002. | *There is no shared and unique definition of SFSC within the scientific community (Kneafsey et al., 2013). The most intuitive and frequently cited feature of SFSC is geographical proximity, that is, the closeness between producers and consumers (Ilbery and Maye, 2006; Kebir and Torre, 2013). This closeness can be conceptualised in terms of political boundaries, that is, in terms of regions or countries (Zepeda and Leviten-Reid, 2004; Engelseth and Hogset, 2016), or in relation to distance, whether measured in kilometres (Chambers et al., 2007; Durham et al., 2009) […] [T]he distance between producers and consumers is not unambiguously defined, but it is a function of the morphological and demographical characteristics of a territory as well as of the actors involved and their objectives.*  *Marsden et al. (2000) put the ‘emphasis upon the type of relationship between the producer and the consumer in these supply chains, and the role of this relationship in constructing value and meaning, rather than solely the type of product itself’. SFSC is based on the direct contact between farmers and consumers and their ensuing relationships based on trust and honesty. SFSC is characterised by a small number/absence of intermediaries (Parker, 2005; Kneafsey et al., 2013). The chain and any possible intermediates must provide a means of communication between farmers and customers: farmers can supply consumers with information and receive their feedback in return (Galli and Brunori, 2013).*  *The European Union (Reg.1305/13) provided a broad definition that includes both ‘social’ proximity (minimum or null number of actors) and geographical proximity (physical distance between farmer and consumers): SFSC is ‘a supply chain involving a limited number of economic operators committed to cooperation, local economic development, and close geographical and social relations between producers, processors and consumers’ (European Union, 2013).*  *The Slow Food association provides another exhaustive and deep definition (Slow Food, 2013): ‘A short food supply chain is created when producers and final consumers realise they share the same goals, which can be achieved by creating new opportunities that strengthen local food networks. It is an alternative strategy that enables producers to regain an active role in the food system, as it focuses on local production – decentralised regional food systems that minimise the number of steps involved and the distance travelled by food (food miles).’*  *SFSC can be categorised as a form of sustainable supply chain (Rajesh, 2018), as it encompasses environmental objectives, but it also focuses on a social and ethical matter. SFSC is described as a Value Chain: it ensures social and economic benefits for supply chain actors, and it does not permanently deplete natural resources (FAO, 2014a). It implicates a high level of trust, transparency, cooperation and shared governance between supply chain actors (Taylor, 2005; Stevenson and Pirog, 2008). Schmitt et al. (2018) identified seven criteria of localness: distance, supply chain size, number of intermediaries by the typology of sales channels, percentage of direct sales, local knowhow, product identity in relation to territory, governance (degree of control of local actors). The different combinations of these criteria are associated with a different degree of localness.*  *Regardless of the specific form it assumes, SFSC represents an alternative food system that aims to achieve sustainability goals. Sustainability is not a status to be achieved, but a continuous process (Li et al., 2014; Brunori et al., 2016). Therefore an interesting approach could be reasoning in terms of the objectives pursued by SFSC. By analysing the main goals of alternative food systems (Cleveland et al., 2015) it is possible to highlight the sustainability objectives they aim to reach. The structure proposed by the SAFA (Sustainability Assessment of Food and Agriculture systems) guidelines (FAO, 2014b), a holistic global framework for the assessment of sustainability along food and agriculture value chains, are taken as reference. As for the SAFA vision, the food and agriculture systems worldwide are characterised by four dimensions of sustainability: good governance, environmental integrity, economic resilience and social well-being.* |

*Figure 5: Examples of the definition and discussion of the SFSC concept in three literature reviews focusing on this concept.*

- 1. **Appendix 4: References**

Aubert, M., 2013. Déterminants de la commercialisation en circuit court: Quels exploitants, sur quelles exploitations ?, 16.

Aubry, C. C., Bressoud, F. F. and Petit, C. C., 2011. Les circuits courts en agriculture revisitent-ils l’organisation du travail dans l’exploitation ? *In*: *Le travail en agriculture : son organisation et ses valeurs face à l’innovation*. Editeur L’Harmattan.

Augère-Granier, M.-L., 2016. *Short Food Supply Chains and Local Food Supply Chains in the EU* [online]. European Parliamentary Research Service. Briefing European Parliament. Available from: https://www.europarl.europa.eu/RegData/etudes/BRIE/2016/586650/EPRS_BRI(2016)586650_EN.pdf [Accessed 7 Jul 2022].

Azima, S. and Mundler, P., 2022. Does direct farm marketing fulfill its promises? analyzing job satisfaction among direct-market farmers in Canada. *Agriculture and Human Values*, 39 (2), 791–807.

Barisaux, M., Gasselin, P. and Laurens, L., 2022. Pourquoi et comment conduire une Scoping Review de la littérature en Sciences Humaines et Sociales ? Presented at the Journées des Recherches en Sciences Sociales, Clermont-Ferrand.

Bayir, B., Charles, A., Sekhari, A. and Ouzrout, Y., 2022. Issues and Challenges in Short Food Supply Chains: A Systematic Literature Review. *Sustainability (Switzerland)*, 14 (5).

Benedek, Z., Fertő, I. and Molnár, A., 2018. Off to market: but which one? Understanding the participation of small-scale farmers in short food supply chains—a Hungarian case study. *Agriculture and Human Values*, 35 (2), 383–398.

Bouba-Olga, O., Carrincazeaux, C., Coris, M. and Ferru, M., 2015. Proximity Dynamics, Social Networks and Innovation. *Regional Studies*, 49 (6), 901–906.

Chiffoleau, Y., 2017. Dynamique des identités collectives dans le changement d’échelle des circuits courts alimentaires. *Revue Française de Socio-Économie*, 18 (1), 123–141.

Chiffoleau, Y. and Dourian, T., 2020. Sustainable Food Supply Chains: Is Shortening the Answer? A Literature Review for a Research and Innovation Agenda. *Sustainability*, 12 (23), 9831.

Chiffoleau, Y., Gauche, A. and Ollivier, 2013. *Impacts Sociaux des circuits courts alimentaires sur les exploitations agricoles. Diversité des modèles et analyses croisées* [online]. IDELE, INRA, CERD, TRAME. Available from: https://chambres-agriculture.fr/fileadmin/user_upload/National/002_inst-site-chambres/pages/exploitation_agri/Impacts_sociaux_des_circuits_courts_alimentaires_sur_les_exploitations_agricoles-ilovepdf-compressed.pdf.

Dedieu, B., 2019. Transversal views on work in agriculture. *Cahiers Agricultures*, 28, 8.

Deverre, C. and Lamine, C., 2010. Les systèmes agroalimentaires alternatifs. Une revue de travaux anglophones en sciences sociales. *Économie rurale. Agricultures, alimentations, territoires*, (317), 57–73.

Dujarier, M.-A., 2021. *Troubles dans le travail. Sociologie d’une catégorie de pensée*. Puf.

Dupré, L., Lamine, C. and Navarrete, M., 2017. Short Food Supply Chains, Long Working Days: Active Work and the Construction of Professional Satisfaction in French Diversified Organic Market Gardening. *Sociologia Ruralis*, 57 (3), 396–414.

Duval, J., Cournut, S. and Hostiou, N., 2021. Livestock farmers’ working conditions in agroecological farming systems. A review. *Agronomy for Sustainable Development*, 41 (2), 22.

Enthoven, L. and Van den Broeck, G., 2021. Local food systems: Reviewing two decades of research. *Agricultural Systems*, 193, 103226.

Evola, R. S., Peira, G., Varese, E., Bonadonna, A. and Vesce, E., 2022. Short Food Supply Chains in Europe: Scientific Research Directions. *Sustainability*, 14 (6), 3602.

Galt, R., 2013. The Moral Economy Is a Double-edged Sword: Explaining Farmers’ Earnings and Self-exploitation in Community-Supported Agriculture. *ECONOMIC GEOGRAPHY*, 89 (4), 341–365.

Gasselin, P., Lardon, S., Cerdan, C., Loudiyi, S. and Sautier, D., 2023. General Introduction. Questions, Issues and Analytical Framework. *In*: Gasselin Pierre (ed.), Lardon Sylvie (ed.), Cerdan Claire (ed.), Loudiyi Salma (ed.), Sautier Denis (ed.). Coexistence and confrontation of agricultural and food models. A new paradigm of territorial development?. Dordrecht : Springer; Ed. Quae. xix–xxxiii.

Haddaway, N. R., Grainger, M. J. and Gray, C. T., 2022. Citationchaser: A tool for transparent and efficient forward and backward citation chasing in systematic searching. *Research Synthesis Methods*, 13 (4), 533–545.

Heinisch, C., Gasselin, P. and Durand, G., 2014a. Circuits alimentaires de proximité dans les Andes. Vers une reconnaissance de l’agriculture familiale et paysanne. *Économie rurale. Agricultures, alimentations, territoires*, (343), 71–86.

Heinisch, C., Gasselin, P. and Durand, G., 2014b. Circuits alimentaires de proximité dans les Andes. Vers une reconnaissance de l’agriculture familiale et paysanne. *Économie rurale. Agricultures, alimentations, territoires*, (343), 71–86.

Hostiou, N., Vollet, D., Benoit, M. and Delfosse, C., 2020. Employment and farmers’ work in European ruminant livestock farms: A review. *Journal of Rural Studies*, 74, 223–234.

Kebir, L. and Torre, A., 2013. Geographical proximity and new short supplyfood chains. *In*: *Creative Industries and Innovation in Europe, Concepts, Measures, and Comparatives Case Studies*. New-York: Routledge.

Kneafsey, M., Venn, L., Schmutz, U., Balasz, B., Trenchard, L., Eyden-Wood, T., Bos, E., Sutton, G. and Blackett, M., 2013. *Short Food Supply Chains and Local Food Systems in the EU. A State of Play of their Socio-Economic Characteristics.* [online]. European Commision. Available from: https://publications.jrc.ec.europa.eu/repository/handle/JRC80420 [Accessed 20 Mar 2023].

Kumar, A., Mangla, S. K. and Kumar, P., 2022. An integrated literature review on sustainable food supply chains: Exploring research themes and future directions. *Science of The Total Environment*, 821, 153411.

Le Bahers, G., Paturel, D., Théodore, M. and Villarroel, A., 2016. Métiers Circuits Courts : comprendre l’évolution des métiers des agriculteurs engagés dans les démarches de circuits courts pour mieux accompagner leur professionnalisation. *Innovations Agronomiques*, 49, 297–308.

Malak-Rawlikowska, A., Majewski, E., Wąs, A., Borgen, S. O., Csillag, P., Donati, M., Freeman, R., Hoàng, V., Lecoeur, J.-L., Mancini, M. C., Nguyen, A., Saïdi, M., Tocco, B., Török, Á., Veneziani, M., Vittersø, G. and Wavresky, P., 2019. Measuring the Economic, Environmental, and Social Sustainability of Short Food Supply Chains. *Sustainability*, 11 (15), 4004.

Malanski, P. D., Schiavi, S. and Dedieu, B., 2019. Characteristics of “work in agriculture” scientific communities. A bibliometric review. *Agronomy for Sustainable Development*, 39 (4), 36.

Malanski P., Dedieu B., Schiavi S., 2021. Mapping the research domains on work in agriculture. A bibliometric review from Scopus database. Journal of Rural Studies 81 (2021) 305–314 https://doi.org/10.1016/j.jrurstud.2020.10.050

Marsden, T., Banks, J. and Bristow, G., 2000. Food Supply Chain Approaches: Exploring their Role in Rural Development. *Sociologia Ruralis*, 40 (4), 424–438.

Martin, T., Gasselin, P., Hostiou, N., Feron, G., Laurens, L., Purseigle, F. and Ollivier, G., 2022. Robots and transformations of work in farm: a systematic review of the literature and a research agenda. *Agronomy for Sustainable Development* [online], 42 (4). Available from: https://link.springer.com/epdf/10.1007/s13593-022-00796-2 [Accessed 13 Jul 2022].

Martinez, S. and Park, T., 2021. *Marketing Practices and Financial Performance of Local Food Producers: A Comparison of Beginning and Experienced Farmers* [online]. U.S. Departement of Agriculture. Economic Information Bulletin. Available from: http://www.ers.usda.gov/publications/pub-details/?pubid=101785 [Accessed 11 Apr 2023].

Methley, A. M., Campbell, S., Chew-Graham, C., McNally, R. and Cheraghi-Sohi, S., 2014. PICO, PICOS and SPIDER: a comparison study of specificity and sensitivity in three search tools for qualitative systematic reviews. *BMC Health Services Research*, 14 (1), 579.

Michel-Villarreal, R., Hingley, M., Canavari, M. and Bregoli, I., 2019. Sustainability in Alternative Food Networks: A Systematic Literature Review. *Sustainability*, 11 (3), 859.

Morsel, N. and Garambois, N., 2022. Agroecology in the Limousin Mountains: Relocating and Diversifying Food Production to Encourage Employment and Conserve Semi-Natural Spaces. *Revue de Geographie Alpine*, 110 (2).

Mundler, P. and Jean-Gagnon, J., 2020. Short food supply chains, labor productivity and fair earnings: an impossible equation? *Renewable Agriculture and Food Systems*, 35 (6), 697–709.

Munn, Z., Peters, M. D. J., Stern, C., Tufanaru, C., McArthur, A. and Aromataris, E., 2018. Systematic review or scoping review? Guidance for authors when choosing between a systematic or scoping review approach. *BMC Medical Research Methodology*, 18 (1), 143.

Ouzzani, M., Hammady, H., Fedorowicz, Z. and Elmagarmid, A., 2016. Rayyan—a web and mobile app for systematic reviews. *Systematic Reviews*, 5 (1), 210.

Paciarotti, C. and Torregiani, F., 2021. The logistics of the short food supply chain: A literature review. *Sustainable Production and Consumption*, 26, 428–442.

Page, M. J., McKenzie, J. E., Bossuyt, P. M., Boutron, I., Hoffmann, T. C., Mulrow, C. D., Shamseer, L., Tetzlaff, J. M., Akl, E. A., Brennan, S. E., Chou, R., Glanville, J., Grimshaw, J. M., Hróbjartsson, A., Lalu, M. M., Li, T., Loder, E. W., Mayo-Wilson, E., McDonald, S., McGuinness, L. A., Stewart, L. A., Thomas, J., Tricco, A. C., Welch, V. A., Whiting, P. and Moher, D., 2021. The PRISMA 2020 statement: an updated guideline for reporting systematic reviews. *BMJ*, 372, n71.

Paranthoën, J.-B. and Wavresky, P., 2021. Travailler en circuits courts : des exploitations sous tensions. *La nouvelle revue du travail* [online], (18). Available from: https://journals.openedition.org/nrt/8240 [Accessed 17 Jan 2022].

Petticrew, M. and Roberts, H., 2006. *Systematic Reviews in the Social Sciences*. Blackwell Publishing.

Philipon, P., Chiffoleau, Y. and Wallet, F., 2017. *Et si on mangeait local?* [online]. Editions Quae. Available from: https://hal.archives-ouvertes.fr/hal-01606209 [Accessed 20 May 2022].

Renkema, M. and Hilletofth, P., 2022. Intermediate short food supply chains: a systematic review. *British Food Journal*, 124 (13), 541–558.

Schreiber, K., Soubry, B., Dove-McFalls, C. and MacDonald, G. K., 2023. Untangling the role of social relationships for overcoming challenges in local food systems: a case study of farmers in Québec, Canada. *Agriculture and Human Values*, 40 (1), 141–156.

United Nations Industrial Development Organization, 2020. *Short Food Supply Chains for promoting local food on local markets* [online]. United Nations Industrial Development Organization. Available from: https://hub.unido.org/sites/default/files/publications/SHORT%20FOOD%20SUPPLY%20CHAINS.pdf [Accessed 20 Mar 2023].

Weiler, A. M., Otero, G. and Wittman, H., 2016. Rock Stars and Bad Apples: Moral Economies of Alternative Food Networks and Precarious Farm Work Regimes. *Antipode*, 48 (4), 1140–1162.

1. Existing in Japan since the 1970s, a ‘teikei’ is an association for the preservation of peasant agriculture, with consumers buying food products, often organic, directly from farmers. [↑](#footnote-ref-1)
2. First introduced in the USA in the 1980s, and then appearing in other countries, Community-Supported Agriculture is a partnership between farmers and consumers in which the responsibilities, risks and benefits of farming are shared. [↑](#footnote-ref-2)
