# Supplementary material for: The work of farmers in short food supply chains: Systematic literature review and research agenda
Source: PLoS One. 2025 Jun 4;20(6):e0314175. doi: 10.1371/journal.pone.0314175 (PMC12136416; doi:10.1371/journal.pone.0314175)
Supplement: S2 Table — (DOCX) [file pone.0314175.s002.docx]

**Full-text records included and records excluded with reason for exclusion**

**Supplementary table 2a : Full-text records included (n=79)**

| Authors | | Year | Title | Journal | DOI | Country of the study | Source |
| --- | --- | --- | --- | --- | --- | --- | --- |
| Black, JE | 2024 | | Fun, community, and culture in a Japanese alternative food network | Frontiers in Sustainable Food Systems | 10.3389/fsufs.2024.1346129 | Japan | Query |
| Chavez-Miguel, G.; Hämmerle, J.; González, A.; Canetti, C.; Gleich, P.; Halfast, R.L.; Feuchter, M.; Buszydlo, D.; Schwarz, L.; Scheepstra, I.; de Haan, S.; Ccanto, R.; Sieber, S.; Bonatti, M. | 2024 | | Local food system resilience in the context of shocks and crises: vulnerabilities and responses of agroecology-based farmers in Peru, Germany, and the United States | Agroecology and Sustainable Food Systems | 10.1080/21683565.2024.2341987 | Several countries | Query |
| Flachs, A.; Raturi, A.; Low, M.; Miller, V.; Norton, J.; Redmond, C.; Thomas, H. | 2024 | | Digital tools for local farmers: Thinking with spreadsheets in the wake of the COVID-19 pandemic | Culture, Agriculture, Food ans Environment | 10.1111/cuag.12316 | US | Query |
| Hvitsand, C.; Nicolaysen, A.M.; Gjotterud, S.; Raanaas, R.K. | 2024 | | Piloting a co-created local and alternative food network involving professional buyers in Norway: Forces and tensions influencing viability | Journal of Rural Studies | 10.1016/j.jrurstud.2024.103362 | Norway | Query |
| Kondo, C., Zollet, S., Kobayashi, M., Yamamoto, N. | 2024 | | Fifty years of Teikei: the evolution of the movement’s ten principles and its impact on alternative food initiatives in Japan | Frontiers in Sustainable Food Systems | 10.3389/fsufs.2024.1368253 | Japan | Query |
| O'Connor, G.; Reis, K.; Desha, C.; Burkett, I. | 2024 | | Valuing farmers in transitions to more sustainable food systems: A systematic literature review of local food producers' experiences and contributions in short food supply chains | Agriculture and Human Values | 10.1007/s10460-024-10601-3 | All | Query |
| Paraušić, V., Dashi, E.M., Subić, J., Pomianek, I., Šarić, B.B. | 2024 | | Response of Short Food Supply Chains in Western Balkan countries to the covid crise : a case study in honey sector | European Countryside | 10.2478/euco-2024-0006. | Several countries | Query |
| Rossi, A.; Piccoli, A.; Feola, G. | 2024 | | Transforming labour around food? the experience of community supported agriculture in Italy | Agriculture and Human Values | 10.1007/s10460-024-10572-5 | Italy | Query |
| Rosman, A.; Macpherson, J.; Arndt, M.; Helming, K. | 2024 | | Perceived resilience of community supported agriculture in Germany | Agricultural Systems | 10.1016/j.agsy.2024.104068 | Germany | Query |
| Savels R., Dessein J.; Lucantoni D.; Speelman S. | 2019 | | Assessing the agroecological performance and sustainability of community supported agriculture farms in Flanders, Belgium | Frontiers in Sustainable Food Systems | 10.3389/fsufs.2024.1359083 | Belgium | Citation Chasing |
| Wang M.; Kumar V.; Ruan X.; Mülling Neutzling D. | 2019 | | farmers’ attitudes towards participation in short food supply chains: evidence from a Chinese field research | Revista Ciências Administrativas | 10.5020/2318-0722.2018.9067 | China | Citation Chasing |
| Azima, S.; Mundler, P. | 2023 | | Farmer satisfaction and short food supply chains | Agriculture and Human Values | 10.1007/s10460-023-10447-1 | Canada | Query |
| Boillat, S.; Bottazzi, P.; Sabaly, I. K. | 2023 | | The division of work in Senegalese conventional and alternative food networks: a contributive justice perspective | Frontiers in Sustainable Food Systems | 10.3389/fsufs.2023.1127593 | Senegal | Query |
| Chiaverina, P.; Drogué, S.; Jacquet, F.; Lev, L.; King, R. | 2023 | | Does short food supply chain participation improve farm economic performance? A meta‐analysis | Agricultural Economics | 10.1111/agec.12764 | Several countries | Citation Chasing |
| Egli, L.; Rüschhoff, J.; Priess, J. | 2023 | | A systematic review of the ecological, social and economic sustainability effects of community-supported agriculture | Frontiers in Sustainable Food Systems | 10.3389/fsufs.2023.1136866 | All | Query |
| Kelmenson, S. | 2023 | | Between the farm and the fork: job quality in sustainable food systems | Agriculture and Human Values | 10.1007/s10460-022-10362-x | US | Query |
| Parausic, V.; Kolasinac, S.; Muca (Dashi), E.; Saric, B.B. | 2023 | | Competencies of Western Balkan farmers for participating in short food supply chains: Honey case study | New medit | 10.30682/nm2304e | Several countries | Query |
| Raj, G.; Feola, G.; Runhaar, H. | 2023 | | Work in progress: power in transformation to postcapitalist work relations in community–supported agriculture | Agriculture and Human Values | 10.1007/s10460-023-10486-8 | Portugal | Citation Chasing |
| Rode, E.L., Rover, O.J., Desconti, C. | 2023 | | Agroecology and direct sale of organic food: a study of two marketing experiences in Santa Catarina, Brazil | International Journal of Sustainable Development and Planning | 10.5380/dma.v62i0.81710 | Brazil | Query |
| Schreiber, K; Soubry, B; Dove-McFalls, C; MacDonald, GK | 2023 | | Untangling the role of social relationships for overcoming challenges in local food systems: a case study of farmers in Quebec, Canada | Agriculture and Human Values | 10.1007/s10460-022-10343-0 | Canada | Query |
| Sulistyowati, C.A.; Afiff, S.A.; Baiquni, M.; Siscawati, M. | 2023 | | Challenges and potential solutions in developing community supported agriculture: a literature review | Agroecology and Sustainable Food Systems | 10.1080/21683565.2023.2187002 | All | Query |
| Azima, S.; Mundler, P. | 2022 | | The gendered motives and experiences of Canadian women farmers in short food supply chains: Work satisfaction, values of care, and the potential for empowerment | Journal of Rural Studies | 10.1016/j.jrurstud.2022.10.007 | Canada | Query |
| Azima, S; Mundler, P. | 2022 | | Does direct farm marketing fulfill its promises? analyzing job satisfaction among direct-market farmers in Canada | Agriculture and Human Values | 10.1007/s10460-021-10289-9 | Canada | Query |
| Bezerra, A.G.C.; Soler Montiel, M.; Butto Zarzar, A.L.; García Roces, I. | 2022 | | Gender, agroecology and local markets at São José de Mipibu in Rio Grande do Norte, Brazil | Agroecology and Sustainable Food Systems | 10.1080/21683565.2021.2025193 | Brazil | Query |
| Birtalan, I.L.; Ferto, I.; Neulinger, A.; Racz, J.; Olah, A. | 2022 | | The wellbeing paradox in Hungarian local sustainable agriculture: a health psychology approach | BMC Public Heatlh | 10.1186/s12889-022-14643-2 | Hungary | Query |
| Florick, L.; Park, C.H. | 2022 | | A pilot study exploring the impacts of COVID-19 on small-scale direct-marketing farmers in Northwest Arkansas and their responses to the pandemic | Journal of Agriculture Food Systems and Community Development | 10.5304/jafscd.2022.121.006 | US | Query |
| Leitheiser, S.; Horlings, I.; Franklin, A.; Trell, E.-M. | 2022 | | Regeneration at a distance from the state: From radical imaginaries to alternative practices in Dutch farming | Sociologia Ruralis | 10.1111/soru.12403 | Netherlands | Query |
| Morsel, N.; Garambois, N. | 2022 | | Agroecology in the Limousin Mountains: Relocating and Diversifying Food Production to Encourage Employment and Conserve Semi-Natural Spaces | Revue de Geographie Alpine | 10.4000/rga.10633 | France | Query |
| Nichols, D.C.; Janssen, D.B.; Beamer, C.; Ferring, C. | 2022 | | Pivoting is exhausting: A critical analysis of local food system resilience | Journal of Rural Studies | 10.1016/j.jrurstud.2022.10.024 | US | Query |
| Schreiber, K.; Soubry, B.; Dove-McFalls, C.; MacDonald, G.K. | 2022 | | Diverse adaptation strategies helped local food producers cope with initial challenges of the Covid-19 pandemic: Lessons from Québec, Canada | Journal of Rural Studies | 10.1016/j.jrurstud.2022.02.002 | Canada | Query |
| Benedek, Z.; Fertö, I.; Marreiros, C.G.; De Aguiar, P.M.; Pocol, C.B.; Čechura, L.; Poder, A.; Pääso, P.; Bakucs, Z. | 2021 | | Farm diversification as a potential success factor for small-scale farmers constrained by COVID-related lockdown. Contributions from a survey conducted in four European countries during the first wave of COVID-19 | PLoS ONE | 10.1371/journal.pone.0251715 | Several countries | Query |
| Biazoti, A.R.; Nakamura, A.C.; Nagib, G.; Leão, V.O.P.S.; Giacchè, G.; Mauad, T. | 2021 | | The impact of covid-19 on urban agriculture in São Paulo, Brazil | Sustainability (Switzerland) | 10.3390/su13116185 | Brazil | Query |
| Bui, T. N.; Nguyen, A.H.; Le, T.T.H.; Nguyen, V.P.; Le T.T.H.; Tran, T.T.H.; Nguyen, T.K.O.; Le, T.K.O.; Nguyen, T.K.O.; Nguyen, T.T.T., Dao, H.V.; Doan, T.N.T. ; Vu, T.H.N.; Bui, V.H., Hoa, H.C. ; Lebailly, P. | 2021 | | Can a short food supply chain create sustainable benefits for small farmers in developing countries? an exploratory study of Vietnam | Sustainability (Switzerland) | 10.3390/su13052443 | Vietnam | Citation Chasing |
| Campos, A.S.N.; Satolo, E.G.; Mac-Lean, P.A.B.; Júnior, S.S.B. | 2021 | | Economic sustainability analysis of the specialty coffee farmers in Garça/SP | Coffee Science | 10.25186/.V16I.1993 | Brazil | Query |
| Drottberger, A.; Melin, M.; Lundgren, L. | 2021 | | Alternative food networks in food system transition—values, motivation, and capacity building among young Swedish market gardeners | Sustainability (Switzerland) | 10.3390/su13084502 | Sweden | Query |
| Hoang, V. | 2021 | | Modern short food supply chain, good agricultural practices, and sustainability: A conceptual framework and case study in Vietnam | Agronomy | 10.3390/agronomy11122408 | Vietnam | Query |
| Kondo, C. | 2021 | | Re-energizing Japan's teikei movement: Understanding intergenerational transitions of diverse economies | Journal of Agriculture Food Systems and Community Development | 10.5304/jafscd.2021.104.031 | Japan | Query |
| Medici, M.; Canavari, M.; Castellini, A. | 2021 | | Exploring the economic, social, and environmental dimensions of community-supported agriculture in Italy | Journal of Cleaner Production | 10.1016/j.jclepro.2021.128233 | Italy | Query |
| Koretskaya, O.; Feola, G. | 2020 | | A framework for recognizing diversity beyond capitalism in agri-food systems | Journal of Rural Studies | 10.1016/j.jrurstud.2020.10.002 | All | Query |
| Mundler, P.; Jean-Gagnon, J. | 2020 | | Short food supply chains, labor productivity and fair earnings: An impossible equation? | Renewable Agriculture and Food Systems | 10.1017/S1742170519000358 | Canada | Query |
| Rucabado-Palomar, T.; Cuéllar-Padilla, M. | 2020 | | Short food supply chains for local food: A difficult path | Renewable Agriculture and Food Systems | 10.1017/S174217051800039X | Spain | Query |
| Beingessner, N.; Fletcher, A.J. | 2019 | | “Going local”: farmers’ perspectives on local food systems in rural Canada | Agriculture and Human Values | 10.1007/s10460-019-09975-6 | Canada | Citation Chasing |
| Bruce, A.B. | 2019 | | Farm entry and persistence: Three pathways into alternative agriculture in southern Ohio | Journal of Rural Studies | 10.1016/j.jrurstud.2019.04.007 | US | Query |
| Charatsari, C. ; Kitsios, F.; Lioutas, E.D. | 2019 | | Short food supply chains: the link between participation and farmers' competencies | Renewable Agriculture and Food Systems | 10.1017/s1742170519000309 | Greece | Citation Chasing |
| Eckert Matzembacher, E.; Bittencourt Meira, F. | 2019 | | Sustainability as business strategy in community supported agriculture | British Food Journal | 10.1108/bfj-03-2018-0207 | Brazil | Citation Chasing |
| Jablonski, B. B. R.; Sullins, M.; McFadden, D.T. | 2019 | | Community-Supported Agriculture Marketing Performance: Results from Pilot Market Channel Assessments in Colorado | Sustainability | 10.3390/su11102950 | US | Citation Chasing |
| Liu, P., Ravenscroft, N.; Ding, D.; Li, D. | 2019 | | From pioneering to organised business: the development of ecological farming in China | Local Environment | 10.1080/13549839.2019.1597032 | Several countries | Query |
| Malak-Rawlikowska, A.; Majewski, E.; Wąs A.; Ole Borgen, S.; Csillag, P.; Donati, M.; Freeman, R.; Hoàng, V.; Lecoeur, J.-L.; Mancini, M.C. ; Nguyễn, A.; Saïdi, M.; Tocco, B.; Török, A.; | 2019 | | Measuring the economic, environmental, and social sustainability of short food supply chains | Sustainability | 10.3390/su11154004 | Several countries | Citation Chasing |
| de Mansoldo M.; Cavagnaro E.; de Oliveira Menezes V. | 2019 | | Farmers’ trade skills: exploring the local food chain in Leeuwarden, the 2018 European capital of culture | Research in Hospitality Management | 10.1080/22243534.2019.1653584 |  | Citation Chasing |
| Sureau, S.; Lohest, F.; Van Mol, J.; Bauler, T.; Achten, W.M.J. | 2019 | | How do chain governance and fair trade matter? A S-LCA methodological proposal applied to food products from Belgian alternative chains (Part 2) | Resources | 10.3390/resources8030145 | Belgium | Query |
| Dunay, A.; Lehota, J; Mácsai, E.C.; Bálint Illés, C. | 2018 | | short supply chain: goals, objectives and attitudes of producers | Acta Polytechnica Hungarica | 10.12700/aph.15.6.2018.6.17 |  | Citation Chasing |
| Parodi, G. | 2018 | | Agroecological transition and reconfiguration of horticultural work among family farmers in Buenos Aires, Argentina | Cahiers Agricultures | 10.1051/cagri/2018020 | Argentina | Query |
| Paul, M. | 2018 | | Community‐supported agriculture in the United States: Social, ecological, and economic benefits to farming | Journal of Agrarian Change | 10.1111/joac.12280 | US | Citation Chasing |
| Bruce, A.B.; Som Castellano, R.L. | 2017 | | Labor and alternative food networks: Challenges for farmers and consumers | Renewable Agriculture and Food Systems | 10.1017/S174217051600034X | US | Query |
| Chaparro Africano, A.; Calle Collado, Á. | 2017 | | Peasant economy sustainability in peasant markets, Colombia | Agroecology and Sustainable Food Systems | 10.1080/21683565.2016.1266069 | Colombia | Query |
| Dupré, L.; Lamine, C.; Navarrete, M. | 2017 | | Short Food Supply Chains, Long Working Days: Active Work and the Construction of Professional Satisfaction in French Diversified Organic Market Gardening | Sociologia Ruralis | 10.1111/soru.12178 | France | Query |
| Leiper, C.; Clarke-Sather, A. | 2017 | | Co-creating an alternative: the moral economy of participating in farmers’ markets | Local Environment | 10.1080/13549839.2017.1296822 | US | Query |
| Lutz, J.; Smetschka, B.; Grima, N. | 2017 | | Farmer cooperation as a means for creating local food systems-Potentials and challenges | Sustainability (Switzerland) | 10.3390/su9060925 | Austria | Query |
| Balázs B.; Balázs B. ; Lazányi O. | 2016 | | Prospects for the future: community supported agriculture in Hungary | Futures | 10.1016/j.futures.2016.03.005 | Hungary | Citation Chasing |
| Biewener, C. | 2016 | | Paid Work, Unpaid Work, and Economic Viability in Alternative Food Initiatives: Reflections from Three Boston Urban Agriculture Endeavors | The Journal of Agriculture, Food Systems, and Community Development | 10.5304/jafscd.2016.062.019 | US | Citation Chasing |
| Hvitsand, C. | 2016 | | Community supported agriculture (CSA) as a transformational act—distinct values and multiple motivations among farmers and consumers | Agroecology and Sustainable Food Systems | 10.1080/21683565.2015.1136720 | Norway | Query |
| MacAuley, L.; Niewolny, K. L. | 2016 | | Situating On-farm Apprenticeships within the Alternative Agrifood Movement: Labor and Social Justice Implications | Journal of Agriculture, Food Systems, and Community Development | 10.5304/jafscd.2016.062.024 | US | Citation Chasing |
| Mincyte, D.; Dobernig, K. | 2016 | | Urban farming in the North American metropolis: Rethinking work and distance in alternative food networks | Environment and Planning A | 10.1177/0308518X16651444 | US | Citation Chasing |
| Mundler, P.; Laughrea, S. | 2016 | | The contributions of short food supply chains to territorial development: A study of three Quebec territories | Journal of Rural Studies | 10.1016/j.jrurstud.2016.04.001 | Canada | Citation Chasing |
| Weiler, A.M.; Otero, G.; Wittman, H. | 2016 | | Rock Stars and Bad Apples: Moral Economies of Alternative Food Networks and Precarious Farm Work Regimes | Antipode | 10.1111/anti.12221 | Canada | Citation Chasing |
| Galt R.E. ; Bradley K.A. ; Christensen L.; Van Soelen Kim J. ; Lobo R. | 2015 | | Eroding the community in Community supported agriculture : competition's effects in alternative food networks in California | Sociologia Ruralis | 10.1111/soru.12102 | US | Citation Chasing |
| Aubry, C.; Kebir, L. | 2013 | | Shortening food supply chains: a means for maintaining agriculture close to urban areas? The case of the French metropolitan area of Paris | Food Policy | 10.1016/j.foodpol.2013.04.006 | France | Query |
| Galt, R.E. | 2013 | | The Moral Economy Is a Double-edged Sword: Explaining Farmers' Earnings and Self-exploitation in Community-Supported Agriculture | Economic Geography | 10.1111/ecge.12015 | US | Query |
| Conner, D.; King, B.R. ; Kolodinsky, J. ; Roche, E. ; Koliba, C.; Trubek, A.B. | 2012 | | You can know your school and feed it too: Vermont farmers’ motivations and distribution practices in direct sales to school food services | Agriculture and Human Values | 10.1007/s10460-012-9357-y | US | Citation Chasing |
| Jarosz, L. | 2011 | | Nourishing women: toward a feminist political ecology of community supported agriculture in the United States | Gender, Place & Culture | 10.1080/0966369x.2011.565871 | US | Citation Chasing |
| Pilgeram, R. | 2011 | | “The Only Thing That Isn't Sustainable . . . Is the Farmer”: Social Sustainability and the Politics of Class among Pacific Northwest Farmers Engaged in Sustainable Farming | Rural Sociology | 10.1111/j.1549-0831.2011.00051.x | US | Citation Chasing |
| Uematsu, H.; Mishra, A. K. | 2011 | | Use of Direct Marketing Strategies by Farmers and Their Impact on Farm Business Income | Agricultural and Resource Economics Review | 10.1017/s1068280500004482 | US | Citation Chasing |
| Hardesty, S.D.; Leff, P. | 2009 | | Determining marketing costs and returns in alternative marketing channels | Renewable Agriculture and Food Systems | 10.1017/s1742170509990196 | US | Citation Chasing |
| Cox, R.; Holloway, L.; Venn, L.; Dowler, L. ; Ricketts, Hein J.; Kneafsey, M. ; Tuomainen, H. | 2008 | | Common ground? motivations for participation in a community-supported agriculture scheme | Local Environment | 10.1080/13549830701669153 | UK | Citation Chasing |
| Trauger, A. | 2007 | | Un/re-constructing the agrarian dream: Going back-to-the-land with an organic marketing co-operative in south-central Pennsylvania, USA | Tijdschrift voor Economische en Sociale Geografie | 10.1111/j.1467-9663.2007.00372.x | US | Query |
| Ross, N.J. | 2006 | | How civic is it? Success stories in locally focused agriculture in Maine | Renewable Agriculture and Food Systems | 10.1079/RAF2005134 | US | Query |
| Ilbery, B.; Maye, D. | 2005 | | Alternative (shorter) food supply chains and specialist livestock products in the Scottish–English borders | Environment and Planning A Economy and Space | 10.1068/a3717 | UK | Citation Chasing |
| Wells, B. L.; Gradwell, S. | 2001 | | Gender and resource management: Community supported agriculture as caring-practice | Agriculture and Human Values | 10.1023/a:1007686617087 | US | Citation Chasing |
| Govindasamy, R.; Hossain, F.; Adelaja, A. O. | 1999 | | Income of farmers who use direct marketing | Agricultural and Resource Economics Review | 10.1017/s106828050000099x | US | Citation Chasing |

**Supplementary Table 2b : Records excluded with reasons for exclusion (n=59)**

*The eligibility criteria are examined one by one. If one of the elements is not present in the text, the paper is considered to be excluded and the examination of the other elements is abandoned.*

| **Authors** | **Year** | **Title** | **Journal** | **DOI** | **Participants : farmers ?** | **Intervention : SFSC ?** | **Outocomes : Dimensions of work ?** | **Farmers' work linked SFSC : central or peripheral result ?** | **Sources** |
| --- | --- | --- | --- | --- | --- | --- | --- | --- | --- |
| Kujala, S., Koppelmäki, K. | 2024 | Regional economic assessment of a novel place-based model for sustainable food systems | Geography and Sustainability | 10.1016/j.geosus.2024.02.002. | NO |  |  |  | Query |
| Lara, L.G.; Feola, G.; Driessen, P. | 2024 | Drawing boundaries: Negotiating a collective 'we' in community-supported agriculture networks | Journal of Rural Studies | 10.1016/j.jrurstud.2024.103197 | NO |  |  |  | Query |
| Leitheiser, S.; Vezzoni, R. | 2024 | Joining the ideational and the material: transforming food systems toward radical food democracy | Frontiers in Sustainable Food Systems | 10.3389/fsufs.2024.1307759 | NO |  |  |  | Query |
| López-García, D. ; Carrascosa-García, M. | 2024 | Sustainable food policies without sustainable farming? Challenges for agroecology-oriented farmers in relation to urban (sustainable) food policies | Journal of Rural Studies | 10.1016/j.jrurstud.2023.103160. | YES | YES | NO |  | Query |
| Balcom, R.; Abebe, G.K.; Yiridoe, E.K.; Hartt, C.M. | 2023 | Sustainable production and distribution practices in Atlantic Canadian short food supply chains: Explorative study | Frontiers in Sustainable Food Systems | 10.3389/fsufs.2023.1121006 | YES | YES | YES | NO | Query |
| Baumann, S.; Johnston, J.; Oleschuk, M. | 2023 | How do producers imagine consumers? Connecting farm and fork through a cultural repertoire of consumer sovereignty | Sociologia Ruralis | 10.1111/soru.12401 | YES | NO |  |  | Query |
| Charatsari, C. ; Lioutas, E.D. ; Michailidis, A. ; Aidonis, D. ; De Rosa, M. ; Partalidou, M. ; Achillas, C. ; Nastis, S. ; Camanzi, L. | 2023 | Facets of value emerging through the operation of short food supply chains | NJAS: Impact in Agricultural and Life Sciences | 10.1080/27685241.2023.2236961 | YES | YES | YES | NO | Query |
| Geissberger, S. ; Chapman, M. | 2023 | The Work that Work does: How intrinsic and instrumental values are transformed into relational values through active work participation in Swiss community supported agriculture | People and Nature | 10.1002/pan3.10531 | NO |  |  |  | Query |
| Kloczko-Gajewska, A.; Malak-Rawlikowska, A.; Majewski, E.; Wilkinson, A.; Gorton, M.; Tocco, B.; Was, A.; Saidi, M.; Torok, A.; Veneziani, M. | 2023 | What are the economic impacts of short food supply chains? A local multiplier effect (LM3) evaluation | European Urban and Regional Studies | 10.1177/09697764231201572 | YES | YES | YES | NO | Query |
| MacKay, C. | 2023 | Grass-Fed Beef, Alterity, and Care: Complicating food Binaries, Relations, and Practices | Journal of Agricultural and Environmental Ethics | 10.1007/s10806-023-09906-w | YES | NO |  |  | Query |
| Apaliya, M.T.; Kwaw, E.; Osae, R.; Alolga, R.N.; Aikins, A.S.S.; Otoo, G.S.; Kaburi, S.A.; Lamptey, F.P.; Amo-Broni, C. | 2022 | THE IMPACT OF COVID-19 ON FOOD SECURITY: GHANA IN REVIEW The impact of Covid-19 on food security : Ghana in review | Journal of Food Technology Research | 10.18488/jftr.v9i3.3228 | NO |  |  |  | Query |
| Ghosh-Jerath, S.; Kapoor, R.; Dhasmana, A.; Singh, A.; Downs, S.; Ahmed, S. | 2022 | Effect of COVID-19 Pandemic on Food Systems and Determinants of Resilience in Indigenous Communities of Jharkhand State, India: A Serial Cross-Sectional Study | Frontiers in Sustainable Food Systems | 10.3389/fsufs.2022.724321 | YES | NO |  |  | Query |
| Parrot, L.; Biard, Y.; Klaver, D.; Kabré, E.; Vannière, H. | 2022 | Slicing the fruit five ways: An economic, social, and environmental assessment of five mango food supply chains in Burkina Faso | Sustainable Production and Consumption | 10.1016/j.spc.2022.01.019 | YES | NO |  |  | Query |
| Thant, P.S.; Espino, A.; Soria, G.; Myae, C.; Rodriguez, E.; Barbon, W.J.; Gonsalves, J. | 2022 | Myanmar local food systems in a changing climate: Insights from multiple stakeholders | Environmental and Sustainability Indicators | 10.1016/j.indic.2022.100170 | NO |  |  |  | Query |
| Ajayi, O.; Ekanem, E.; Mafuyai, M. | 2021 | Economic contributions of the local food systems in tennessee | Journal of Food Distribution Research | 0 | NO |  |  |  | Query |
| Bachman, G.H.; Lupolt, S.N.; Strauss, M.; Kennedy, R.D.; Nachman, K.E. | 2021 | An examination of adaptations of direct marketing channels and practices by Maryland fruit and vegetable farmers during the COVID-19 pandemic | Journal of Agricultural Food Systems and Community Development | 10.5304/jafscd.2021.104.010 | YES | YES | NO |  | Query |
| Canal Vieira, L.; Serrao-Neumann, S.; Howes, M. | 2021 | Daring to build fair and sustainable urban food systems: A case study of alternative food networks in Australia | Agroecology and Sustainable Food Systems | 10.1080/21683565.2020.1812788 | NO |  |  |  | Query |
| Klimek, M.; Bingen, J.; Freyer, B.; Paxton, R. | 2021 | From schnitzel to sustainability: Shifting values at Vienna’s urban farmers markets | Sustainability (Switzerland) | 10.3390/su13158327 | NO |  |  |  | Query |
| Naglis-Liepa, K.; Proškina, L.; Paula, L.; Kaufmane, D. | 2021 | Modelling the multiplier effect of a local food system | Agronomy Research | 10.15159/AR.21.072 | NO |  |  |  | Query |
| Bisht, I.S.; Rana, J.C.; Ahlawat, S.P. | 2020 | The Future of Smallholder Farming in India: Some Sustainability Considerations | Sustainability | 10.3390/su12093751 | YES | YES | NO |  | Query |
| King, H.B. | 2020 | Whose work is real work? A triple labor framework for sustainable development initiatives | Economic Anthropology | 10.1002/sea2.12174 | NO |  |  |  | Query |
| Kurtsal, Y.; Ayalp, E.K.; Viaggi, D. | 2020 | Exploring governance mechanisms, collaborative processes and main challenges in short food supply chains: The case of turkey | Bio-based and Applied Economics | 10.13128/bae-8242 | NO |  |  |  | Query |
| Michel-Villarreal, R.; Vilalta-Perdomo, E.L.; Hingley, M. | 2020 | Exploring producers' motivations and challenges within a farmers' market | British Food Journal | 10.1108/BFJ-09-2019-0731 | YES | YES | NO |  | Query |
| Sitaker, M.; Kolodinsky, J.; Wang, W.; Chase, L.C.; Kim, J.V.S.; Smith, D.; Estrin, H.; Vlaanderen, Z.V.; Greco, L. | 2020 | Evaluation of farm fresh food boxes: A hybrid alternative food network market innovation | Sustainability (Switzerland) | 10.3390/su122410406 | YES | YES | YES | NO | Query |
| Slamet, A.S.; Hadiguna, R.A.; Mulyati, H. | 2020 | Making food supply chain sustainable: Participating smallholder farmers in modern retail channels | International Journal of Sustainable Agricultural Management and Informatics | 10.1504/IJSAMI.2020.108361 | NO |  |  |  | Query |
| Watson, D.J. | 2020 | Working the fields: The organization of labour in community supported agriculture | Organization | 10.1177/1350508419888898 | NO |  |  |  | Query |
| Yu, W.; Spencer, D.M. | 2020 | A qualitative study of visitors to small-scale farms on a tropical Island | Tourism Recreation Research | 10.1080/02508281.2019.1710678 | YES | NO |  |  | Query |
| Zhang, L. | 2020 | From left behind to leader: gender, agency, and food sovereignty in China | Agriculture and Human Values | 10.1007/s10460-020-10114-9 | NO |  |  |  | Query |
| Brulard, N.; Cung, V.-D.; Catusse, N.; Dutrieux, C. | 2019 | An integrated sizing and planning problem in designing diverse vegetable farming systems | International Journal of Production Research | 10.1080/00207543.2018.1498985 | YES | YES | YES | NO | Query |
| Dancer, A.; Newton, P.; House, V. | 2019 | The Difficulties of Developing Local Food Systems: Perspectives of Farmers and Other Key Stakeholders in Boulder County, Colorado | Food Studies | 10.18848/2160-1933/CGP/v09i04/1-20 | YES | YES | YES | NO | Query |
| Dong, H.; Campbell, B.; Rabinowitz, A.N. | 2019 | Factors impacting producer marketing through community supported agriculture | PLoS ONE | 10.1371/journal.pone.0219498 | YES | YES | NO |  | Query |
| Figueroa-Rodríguez, K.A.; álvarez-Ávila, M.D.C.; Castillo, F.H.; Rindermann, R.S.; Figueroa-Sandoval, B. | 2019 | Farmers' market actors, dynamics, and attributes: A bibliometric study | Sustainability (Switzerland) | 10.3390/su11030745 | YES | YES | NO |  | Query |
| Samoggia, A.; Perazzolo, C.; Kocsis, P.; Del Prete, M. | 2019 | Community Supported Agriculture Farmers' Perceptions of Management Benefits and Drawbacks | Sustainability | 10.3390/su11123262 | YES | YES | YES | NO | Query |
| Silva, E.M.; Hendrickson, J.; Mitchell, P.D.; Bietila, E. | 2019 | From the field: A participatory approach to assess labor inputs on organic diversified vegetable farms in the Upper Midwestern USA | Renewable Agriculture and Food Systems | 10.1017/S1742170517000266 | YES | NO |  |  | Query |
| Aguiar, L.C.; DelGrossi, M.E.; Thomé, K.M. | 2018 | Short food supply chain: Characteristics of a family farm | Ciencia Rural | 10.1590/0103-8478cr20170775 | YES | YES | YES | NO | Query |
| Charatsari, C.; Kitsios, F.; Stafyla, A.; Aidonis, D.; Lioutas, E. | 2018 | Antecedents of farmers’ willingness to participate in short food supply chains | British Food Journal | 10.1108/BFJ-09-2017-0537 | YES | YES | NO |  | Query |
| Ding, D.; Liu, P.; Ravenscroft, N. | 2018 | The new urban agricultural geography of Shanghai | Geoforum | 10.1016/j.geoforum.2018.02.010 | NO |  |  |  | Query |
| Skog, K.L.; Eriksen, S.E.; Brekken, C.A.; Francis, C. | 2018 | Building resilience in social-ecological food systems in Vermont | Sustainability (Switzerland) | 10.3390/su10124813 | NO |  |  |  | Query |
| Demartini, E.; Gaviglio, A.; Pirani, A. | 2017 | Farmers’ motivation and perceived effects of participating in short food supply chains: Evidence from a North Italian survey | Agricultural Economics (Czech Republic) | 10.17221/323/2015-AGRICECON | YES | YES | NO |  | Query |
| Mars, M.M.; Schau, H.J. | 2017 | Institutional entrepreneurship and the negotiation and blending of multiple logics in the Southern Arizona local food system | Agriculture and Human Values | 10.1007/s10460-016-9722-3 | NO |  |  |  | Query |
| McLaughlin, J. | 2017 | Strengthening the backbone: Local food, foreign labour and social justice | Nourishing Communities: From Fractured Food Systems to Transformative Pathways | 10.1007/978-3-319-57000-6_2 | YES | NO |  |  | Query |
| Schmit, T.M.; Jablonski, B.B.R.; Mansury, Y. | 2016 | Assessing the Economic Impacts of Local Food System Producers by Scale: A Case Study From New York | Economic Development Quarterly | 10.1177/0891242416657156 | YES | YES | NO |  | Query |
| Migliore, G.; Schifani, G.; Romeo, P.; Hashem, S.; Cembalo, L. | 2015 | Are Farmers in Alternative Food Networks Social Entrepreneurs? Evidence from a Behavioral Approach | Journal of Agricultural and Environmental Ethics | 10.1007/s10806-015-9562-y | YES | YES | NO |  | Query |
| Tudisca, S.; Di Trapani, A.M.; Sgroi, F.; Testa, R. | 2015 | Socio-economic assessment of direct sales in Sicilian farms | Italian Journal of Food Science | 10.14674/1120-1770/ijfs.v80 | YES | YES | YES | NO | Query |
| Cross, P.; Edwards, R.T.; Opondo, M.; Nyeko, P.; Edwards-Jones, G. | 2009 | Does farm worker health vary between localised and globalised food supply systems? | Environment International | 10.1016/j.envint.2009.04.009 | YES | YES | NO |  | Query |
| Follett, J.R. | 2009 | Choosing a food future: Differentiating among alternative food options | Journal of Agricultural and Environmental Ethics | 10.1007/s10806-008-9125-6 | NO |  |  |  | Query |
| Anderson, M.D. | 2008 | Rights-based food systems and the goals of food systems reform | Agriculture and Human Values | 10.1007/s10460-008-9151-z | NO |  |  |  | Query |
| Noireaux, V.; Cassière, F. | 2024 | Role tensions in agricultural distribution in a mid-mountain area | Supply Chain Forum an International Journal | 10.1080/16258312.2024.2329609 | No access |  |  |  | Citation Chasing |
| Enthoven, L.; Skambracks, M.; Van den Broeck, G. | 2023 | Improving the design of local short food supply chains: farmers’ views in Wallonia, Belgium | Journal of Rural Studies | 10.1016/j.jrurstud.2023.01.016 | YES | YES | NO |  | Citation Chasing |
| Vaderna, C.; Home, R.; Migliorini, P.; Roep, D. | 2022 | Overcoming divergence: managing expectations from organisers and members in community supported agriculture in Switzerland | Humanities and Social Sciences Communications | 10.1057/s41599-022-01115-6 | NO |  |  |  | Citation Chasing |
| Vitterso, G. ; Torjusen, H. ; Laitala, K. ; Tocco, B. ; Biasini, B. ; Csillag, P.; Duboys, de Labarre M.; Lecoeur, J.-L. ; Maj, A. ; Majewski, E.; Malak-Rawlikowska, A. ; Menozzi,D. ; Torok, A. ; Wavresky, P. | 2019 | Short food supply chains and their contributions to sustainability: participants’ views and perceptions from 12 european cases | Sustainability | 10.3390/su11174800 | YES | YES | NO |  | Citation Chasing |
| Sellitto M.A. ; Machado Vial L.A. ; Viegas C.L. | 2018 | Critical success factors in short food supply chains: case studies with milk and dairy producers from Italy and Brazil | Journal of Cleaner Production | 10.1016/j.jclepro.2017.09.235 | YES | YES | NO |  | Citation Chasing |
| Schmutz U.; Kneafsey M.; Sarrouy Kay C.; Doernberg A. ; Zasada I. | 2017 | Sustainability impact assessments of different urban short food supply chains: examples from London, UK | Renewable Agriculture and Food Systems | 10.1017/s1742170517000564 | NO |  |  |  | Citation Chasing |
| Chen, W. | 2013 | Perceived value of a community supported agriculture (csa) working share. the construct and its dimensions | Appetite | 10.1007/s10460-012-9357-y | NO |  |  |  | Citation Chasing |
| Brown, C.; Miller, S. | 2008 | The Impacts of Local Markets: A Review of Research on Farmers Markets and Community Supported Agriculture (CSA) | American Journal of Agricultural Economics | 10.1111/j.1467-8276.2008.01220.x | NO | YES | NO |  | Citation Chasing |
| Feenstra, G.W.; Lewis, C.C.; Clare Hinrichs, C.; Gillespie Jt., G.W.; Hilchey, D. | 2003 | Entrepreneurial outcomes and enterprise size in US retail farmers' markets | American Journal of Alternative Agriculture | 10.1079/ajaa2003046 | NO | YES | NO |  | Citation Chasing |
