# Supplementary material for: The work of farmers in short food supply chains: Systematic literature review and research agenda
Source: PLoS One. 2025 Jun 4;20(6):e0314175. doi: 10.1371/journal.pone.0314175 (PMC12136416; doi:10.1371/journal.pone.0314175)
Supplement: S3 Table — (DOCX) [file pone.0314175.s003.docx]

**Supplementary Table 3: Main references cited by the corpus (n=45, times cited >4)**

| Times cited | Publication | In corpus ? |
| --- | --- | --- |
| 25 | Renting, Henk; Marsden, Terry K; Banks, Jo, 2003. Understanding Alternative Food Networks: Exploring the Role of Short Food Supply Chains in Rural Development. Environment and Planning A: Economy and Space. doi:10.1068/a3510 | N |
| 19 | Hinrichs, C.Clare, 2000. Embeddedness and local food systems: notes on two types of direct agricultural market. Journal of Rural Studies. doi:10.1016/s0743-0167(99)00063-7 | N |
| 15 | Galt, Ryan E., 2013. The Moral Economy Is a Double‐edged Sword: Explaining Farmers’ Earnings and Self‐exploitation in Community‐Supported Agriculture. Economic Geography. doi:10.1111/ecge.12015 | Y |
| 11 | Sage, Colin, 2003. Social embeddedness and relations of regard:. Journal of Rural Studies. doi:10.1016/s0743-0167(02)00044-x | N |
| 10 | Jarosz, Lucy, 2008. The city in the country: Growing alternative food networks in Metropolitan areas. Journal of Rural Studies. doi:10.1016/j.jrurstud.2007.10.002 | N |
| 10 | Marsden, Terry; Banks, Jo; Bristow, Gillian, 2000. Food Supply Chain Approaches: Exploring their Role in Rural Development. Sociologia Ruralis. doi:10.1111/1467-9523.00158 | N |
| 10 | Cone, Cynthia Abbott; Myhre, Andrea, 2000. Community-Supported Agriculture: A Sustainable Alternative to Industrial Agriculture?. Human Organization. doi:10.17730/humo.59.2.715203t206g2j153 | N |
| 9 | Allen, Patricia; FitzSimmons, Margaret; Goodman, Michael; Warner, Keith, 2003. Shifting plates in the agrifood landscape: the tectonics of alternative agrifood initiatives in California. Journal of Rural Studies. doi:10.1016/s0743-0167(02)00047-5 | N |
| 9 | Hardesty, Shermain D.; Leff, Penny, 2009. Determining marketing costs and returns in alternative marketing channels. Renewable Agriculture and Food Systems. doi:10.1017/s1742170509990196 | Y |
| 9 | Bruce, Analena B.; Som Castellano, Rebecca L., 2016. Labor and alternative food networks: challenges for farmers and consumers. Renewable Agriculture and Food Systems. doi:10.1017/s174217051600034x | N |
| 8 | DuPuis, E. Melanie; Goodman, David, 2005. Should we go “home” to eat?: toward a reflexive politics of localism. Journal of Rural Studies. doi:10.1016/j.jrurstud.2005.05.011 | N |
| 8 | Tregear, Angela, 2011. Progressing knowledge in alternative and local food networks: Critical reflections and a research agenda. Journal of Rural Studies. doi:10.1016/j.jrurstud.2011.06.003 | N |
| 8 | Winter, Michael, 2003. Embeddedness, the new food economy and defensive localism. Journal of Rural Studies. doi:10.1016/s0743-0167(02)00053-0 | N |
| 8 | Cox, Rosie; Holloway, Lewis; Venn, Laura; Dowler, Liz; Hein, Jane Ricketts; Kneafsey, Moya; Tuomainen, Helen, 2008. Common ground? Motivations for participation in a community-supported agriculture scheme. Local Environment. doi:10.1080/13549830701669153 | Y |
| 8 | Sonnino, Roberta; Marsden, Terry, 2005. Beyond the divide: rethinking relationships between alternative and conventional food networks in Europe. Journal of Economic Geography. doi:10.1093/jeg/lbi006 | N |
| 8 | Hendrickson, Mary K.; Heffernan, William D., 2002. Opening Spaces through Relocalization: Locating Potential Resistance in the Weaknesses of the Global Food System. Sociologia Ruralis. doi:10.1111/1467-9523.00221 | N |
| 8 | Watts, D. C. H.; Ilbery, B.; Maye, D., 2005. Making reconnections in agro-food geography: alternative systems of food provision. Progress in Human Geography. doi:10.1191/0309132505ph526oa | N |
| 7 | Forssell, Sini; Lankoski, Leena, 2014. The sustainability promise of alternative food networks: an examination through “alternative” characteristics. Agriculture and Human Values. doi:10.1007/s10460-014-9516-4 | N |
| 7 | Mundler, Patrick; Laughrea, Sophie, 2016. The contributions of short food supply chains to territorial development: A study of three Quebec territories. Journal of Rural Studies. doi:10.1016/j.jrurstud.2016.04.001 | Y |
| 7 | Jarosz, Lucy, 2011. Nourishing women: toward a feminist political ecology of community supported agriculture in the United States. Gender, Place &amp; Culture. doi:10.1080/0966369x.2011.565871 | Y |
| 7 | Brown, Cheryl; Miller, Stacy, 2008. The Impacts of Local Markets: A Review of Research on Farmers Markets and Community Supported Agriculture (CSA). American Journal of Agricultural Economics. doi:10.1111/j.1467-8276.2008.01220.x | N |
| 7 | Thompson, Craig J.; Coskuner-Balli, Gokcen, 2007. Enchanting Ethical Consumerism. Journal of Consumer Culture. doi:10.1177/1469540507081631 | N |
| 6 | Feagan, Robert; Henderson, Amanda, 2008. Devon Acres CSA: local struggles in a global food system. Agriculture and Human Values. doi:10.1007/s10460-008-9154-9 | N |
| 6 | Hayden, Jennifer; Buck, Daniel, 2012. Doing community supported agriculture: Tactile space, affect and effects of membership. Geoforum. doi:10.1016/j.geoforum.2011.08.003 | N |
| 6 | Ilbery, Brian; Maye, Damian, 2005. Food supply chains and sustainability: evidence from specialist food producers in the Scottish/English borders. Land Use Policy. doi:10.1016/j.landusepol.2004.06.002 | N |
| 6 | Ilbery, Brian; Maye, Damian, 2005. Alternative (Shorter) Food Supply Chains and Specialist Livestock Products in the Scottish–English Borders. Environment and Planning A: Economy and Space. doi:10.1068/a3717 | Y |
| 6 | Paul, Mark, 2018. Community‐supported agriculture in the United States: Social, ecological, and economic benefits to farming. Journal of Agrarian Change. doi:10.1111/joac.12280 | Y |
| 5 | Kloppenburg, Jack; Hendrickson, John; Stevenson, G. W., 1996. Coming in to the foodshed. Agriculture and Human Values. doi:10.1007/bf01538225 | N |
| 5 | Trauger, Amy; Sachs, Carolyn; Barbercheck, Mary; Brasier, Kathy; Kiernan, Nancy Ellen, 2009. “Our market is our community”: women farmers and civic agriculture in Pennsylvania, USA. Agriculture and Human Values. doi:10.1007/s10460-008-9190-5 | N |
| 5 | Aubry, Christine; Kebir, Leïla, 2013. Shortening food supply chains: A means for maintaining agriculture close to urban areas? The case of the French metropolitan area of Paris. Food Policy. doi:10.1016/j.foodpol.2013.04.006 | Y |
| 5 | Feldmann, Corinna; Hamm, Ulrich, 2015. Consumers’ perceptions and preferences for local food: A review. Food Quality and Preference. doi:10.1016/j.foodqual.2014.09.014 | N |
| 5 | Nost, Eric, 2014. Scaling-up local foods: Commodity practice in community supported agriculture (CSA). Journal of Rural Studies. doi:10.1016/j.jrurstud.2014.01.001 | N |
| 5 | Govindasamy, Ramu; Hossain, Ferdaus; Adelaja, Adesoji, 1999. Income of Farmers Who Use Direct Marketing. Agricultural and Resource Economics Review. doi:10.1017/s106828050000099x | Y |
| 5 | Wells, Betty L.; Gradwell, Shelly, 2001. . Agriculture and Human Values. doi:10.1023/a:1007686617087 | Y |
| 5 | DeLind, Laura B., 2002. . Agriculture and Human Values. doi:10.1023/a:1019994728252 | N |
| 5 | Whatmore, Sarah; Stassart, Pierre; Renting, Henk, 2003. What's Alternative about Alternative Food Networks?. Environment and Planning A: Economy and Space. doi:10.1068/a3621 | N |
| 5 | Wilson, Amanda DiVito, 2012. Beyond Alternative: Exploring the Potential for Autonomous Food Spaces. Antipode. doi:10.1111/j.1467-8330.2012.01020.x | N |
| 5 | Goodman, David, 2004. Rural Europe Redux? Reflections on Alternative Agro‐Food Networks and Paradigm Change. Sociologia Ruralis. doi:10.1111/j.1467-9523.2004.00258.x | N |
| 5 | Venn, Laura; Kneafsey, Moya; Holloway, Lewis; Cox, Rosie; Dowler, Elizabeth; Tuomainen, Helena, 2006. Researching European ‘alternative’ food networks: some methodological considerations. Area. doi:10.1111/j.1475-4762.2006.00694.x | N |
| 5 | Galt, Ryan E.; Bradley, Katharine; Christensen, Libby; Van Soelen Kim, Julia; Lobo, Ramiro, 2015. Eroding the Community in Community Supported Agriculture (CSA): Competition's Effects in Alternative Food Networks in <scp>C</scp>alifornia. Sociologia Ruralis. doi:10.1111/soru.12102 | Y |
| 5 | Born, Branden; Purcell, Mark, 2006. Avoiding the Local Trap. Journal of Planning Education and Research. doi:10.1177/0739456x06291389 | N |
| 5 | Cooley, Jack P.; Lass, Daniel A., 1998. Consumer Benefits from Community Supported Agriculture Membership. Review of Agricultural Economics. doi:10.2307/1349547 | N |
| 5 | Murdoch, Jonathan; Marsden, Terry; Banks, Jo, 2000. Quality, Nature, and Embeddedness: Some Theoretical Considerations in the Context of the Food Sector. Economic Geography. doi:10.2307/144549 | N |
| 5 | Malak-Rawlikowska, Agata; Majewski, Edward; Wąs, Adam; Borgen, Svein Ole; Csillag, Peter; Donati, Michele; Freeman, Richard; Hoàng, Viet; Lecoeur, Jean-Loup; Mancini, Maria Cecilia; Nguyen, An; Saïdi, Monia; Tocco, Barbara; Török, Áron; Veneziani, Mario; Vittersø, Gunnar; Wavresky, Pierre, 2019. Measuring the Economic, Environmental, and Social Sustainability of Short Food Supply Chains. Sustainability. doi:10.3390/su11154004 | Y |
| 5 | Galt, Ryan E.; O'Sullivan, Libby; Beckett, Jessica; Hiner, Colleen C., 2012. Community Supported Agriculture is thriving in the Central Valley. California Agriculture. doi:10.3733/ca.v066n01p8 | N |
