# Supplementary material for: The work of farmers in short food supply chains: Systematic literature review and research agenda
Source: PLoS One. 2025 Jun 4;20(6):e0314175. doi: 10.1371/journal.pone.0314175 (PMC12136416; doi:10.1371/journal.pone.0314175)
Supplement: S7 Table — (DOCX) [file pone.0314175.s007.docx]

**List of articles from query output, after duplicate removal (n=494)**

1.

Hatipi NAM, Sarnin SS, Tan MNM, Dohad MR, Kamaruzaman NFEN, Naim NF, et al. IoT-Based Smart Vertical Hydroponic System for Chili Plant. Journal of Advanced Research in Applied Sciences and Engineering Technology. 2025;43: 124–133. doi:[10.37934/araset.43.2.124133](https://doi.org/10.37934/araset.43.2.124133)

2.

Bolang PD, Baddianaah I, Osumanu IK. Towards meeting the food needs of urban households in sub-Saharan Africa: an analysis of formal sector employees’ participation in agricultural production in urban Wa, Ghana. COGENT SOCIAL SCIENCES. 2024;10. doi:[10.1080/23311886.2024.2309711](https://doi.org/10.1080/23311886.2024.2309711)

3.

Rosman A, Macpherson J, Arndt M, Helming K. Perceived resilience of community supported agriculture in Germany. AGRICULTURAL SYSTEMS. 2024;220. doi:[10.1016/j.agsy.2024.104068](https://doi.org/10.1016/j.agsy.2024.104068)

4.

Hvitsand C, Nicolaysen AM, Gjotterud S, Raanaas RK. Piloting a co-created local and alternative food network involving professional buyers in Norway: Forces and tensions influencing viability. JOURNAL OF RURAL STUDIES. 2024;110. doi:[10.1016/j.jrurstud.2024.103362](https://doi.org/10.1016/j.jrurstud.2024.103362)

5.

Winkler G, Kaufmann S, Hruschka N, Vogl CR. Participatory Guarantee Systems: structure, benefits and reasons for participation - insights from the Italian case study of Campi Aperti. FRONTIERS IN SUSTAINABLE FOOD SYSTEMS. 2024;8. doi:[10.3389/fsufs.2024.1388853](https://doi.org/10.3389/fsufs.2024.1388853)

6.

Kondo C, Zollet S, Kobayashi M, Yamamoto N. Fifty years of *Teikei*: the evolution of the movement’s ten principles and its impact on alternative food initiatives in Japan. FRONTIERS IN SUSTAINABLE FOOD SYSTEMS. 2024;8. doi:[10.3389/fsufs.2024.1368253](https://doi.org/10.3389/fsufs.2024.1368253)

7.

Kapoor R, Sabharwal M, Ghosh-Jerath S. Co-existence of potentially sustainable indigenous food systems and poor nutritional status in Ho indigenous community, India: an exploratory study. ENVIRONMENTAL RESEARCH LETTERS. 2024;19. doi:[10.1088/1748-9326/ad4b44](https://doi.org/10.1088/1748-9326/ad4b44)

8.

Zerbian T, Lopez-Garcia D. Navigating agroecological urbanism: examining linkages and interdependencies within alternative food networks. FRONTIERS IN SUSTAINABLE FOOD SYSTEMS. 2024;8. doi:[10.3389/fsufs.2024.1375128](https://doi.org/10.3389/fsufs.2024.1375128)

9.

Mahroof K, Omar A, Yaroson EV, Tenebe SA, Rana NP, Sivarajah U, et al. Evaluating the intention to use Industry 5.0 (I5.0) drones for cleaner production in Sustainable Food Supply Chains: an emerging economy context. SUPPLY CHAIN MANAGEMENT-AN INTERNATIONAL JOURNAL. 2024;29: 468–496. doi:[10.1108/SCM-01-2023-0045](https://doi.org/10.1108/SCM-01-2023-0045)

10.

Alexander A, Kumar M, Walker H, Gosling J. Innovation for zero-deforestation sustainable supply chain management services: a performance measurement and management approach. SUPPLY CHAIN MANAGEMENT-AN INTERNATIONAL JOURNAL. 2024;29: 620–641. doi:[10.1108/SCM-02-2023-0088](https://doi.org/10.1108/SCM-02-2023-0088)

11.

Leitheiser S, Vezzoni R. Joining the ideational and the material: transforming food systems toward radical food democracy. FRONTIERS IN SUSTAINABLE FOOD SYSTEMS. 2024;8. doi:[10.3389/fsufs.2024.1307759](https://doi.org/10.3389/fsufs.2024.1307759)

12.

Black JE. Fun, community, and culture in a Japanese alternative food network. FRONTIERS IN SUSTAINABLE FOOD SYSTEMS. 2024;8. doi:[10.3389/fsufs.2024.1346129](https://doi.org/10.3389/fsufs.2024.1346129)

13.

Parausic V, Dashi EM, Subic J, Pomianek I, Saric BB. Response of Short Food Supply Chains in Western Balkan Countries to the COVID Crisis: A Case Study in the Honey Sector. EUROPEAN COUNTRYSIDE. 2024;16: 86–109. doi:[10.2478/euco-2024-0006](https://doi.org/10.2478/euco-2024-0006)

14.

Nguyen-Trung K, Uekusa S, Matthewman S. Locked into a permanent position of vulnerability? Farmers’ trust and social capital with the government from Critical Disaster Studies perspectives. NATURAL HAZARDS. 2024;120: 4463–4483. doi:[10.1007/s11069-023-06383-2](https://doi.org/10.1007/s11069-023-06383-2)

15.

Stone TF, Thompson JR, Zimmerman E, Brighenti TM, Liebman M. What would it look like? Visualizing a future US Corn Belt landscape with more table food production. RENEWABLE AGRICULTURE AND FOOD SYSTEMS. 2024;39. doi:[10.1017/S1742170524000024](https://doi.org/10.1017/S1742170524000024)

16.

Baker C. Humane dog food? caring and killing in the certified humane dog food value chain. ENVIRONMENT AND PLANNING E-NATURE AND SPACE. 2024;7: 311–329. doi:[10.1177/25148486231165457](https://doi.org/10.1177/25148486231165457)

17.

Zi C, Winterbottom D, Liu J. Strategies for building edible green infrastructure in traditional villages and insights into bio-districts: a case study of Dong villages in Huanggang, China. FRONTIERS IN SUSTAINABLE FOOD SYSTEMS. 2024;8. doi:[10.3389/fsufs.2024.1305094](https://doi.org/10.3389/fsufs.2024.1305094)

18.

Vuksanovic N, Bajrami DD, Petrovic MD, Radovanovic MM, Malinovic-Milicevic S, Radosavac A, et al. The Role of Culinary Tourism in Local Marketplace Business-New Outlook in the Selected Developing Area. AGRICULTURE-BASEL. 2024;14. doi:[10.3390/agriculture14010130](https://doi.org/10.3390/agriculture14010130)

19.

Kristiansen MLT, Gillette MB. Polanyi and the Other Alternative Food Network: What San Francisco-Based Multi-Level Marketers of “Healthy” Food Tell Us About Values in Market Societies. ECONOMIC ANTHROPOLOGY. 2024;11: 100–111. doi:[10.1002/sea2.12298](https://doi.org/10.1002/sea2.12298)

20.

Kpenekuu F, Antwi-Agyei P, Nimoh F, Osei MA, Guodaar L. Understanding drought risk management in vulnerable communities in dryland farming systems: Evidence from northwest Ghana. INTERNATIONAL JOURNAL OF DISASTER RISK REDUCTION. 2024;100. doi:[10.1016/j.ijdrr.2023.104186](https://doi.org/10.1016/j.ijdrr.2023.104186)

21.

Charatsari C, Michailidis A, Francescone M, De Rosa M, Aidonis D, Bartoli L, et al. Do Agricultural Knowledge and Innovation Systems Have the Dynamic Capabilities to Guide the Digital Transition of Short Food Supply Chains? INFORMATION. 2024;15. doi:[10.3390/info15010022](https://doi.org/10.3390/info15010022)

22.

Valle MPV, Viana MM, Alfinito S. FROM COLLECTIVE EXPERIENCE TO SUSTAINABILITY: THE MEANINGS OF BEING PART OF A COMMUNITY-SUPPORTED AGRICULTURE (CSA). Revista Brasileira de Gestao e Desenvolvimento Regional. 2024;20: 494–518. doi:[10.54399/rbgdr.v20i1.6122](https://doi.org/10.54399/rbgdr.v20i1.6122)

23.

Taji K, Sohail A, Shahzad T, Khan BS, Khan MA, Ouahada K. An Ensemble Hybrid Framework: A Comparative Analysis of Metaheuristic Algorithms for Ensemble Hybrid CNN Features for Plants Disease Classification. IEEE ACCESS. 2024;12: 61886–61906. doi:[10.1109/ACCESS.2024.3389648](https://doi.org/10.1109/ACCESS.2024.3389648)

24.

Stephens RJS. Alternative Food Networks and agri-food regime transitions: evolution of institutional knowledge on local food from the fruits and vegetables sector in France. SUSTAINABILITY SCIENCE. 2024. doi:[10.1007/s11625-024-01512-7](https://doi.org/10.1007/s11625-024-01512-7)

25.

Sanz Sanz E, Napoléone C, Debolini M, Martinetti D, Moreno Pérez O, de Benito C, et al. Farmland expansion and intensification do not foster local food self-sufficiency. Insights from the Mediterranean area. Journal of Environmental Management. 2024;351. doi:[10.1016/j.jenvman.2023.119769](https://doi.org/10.1016/j.jenvman.2023.119769)

26.

Saglie I-L. Motivations for Urban Agriculture Policies: Evidence from Norway’s Largest Urban Areas. GeoJournal Library. 2024;132: 237–254. doi:[10.1007/978-3-031-41550-0_11](https://doi.org/10.1007/978-3-031-41550-0_11)

27.

Rossi A, Piccoli A, Feola G. Transforming labour around food? the experience of community supported agriculture in Italy. AGRICULTURE AND HUMAN VALUES. 2024. doi:[10.1007/s10460-024-10572-5](https://doi.org/10.1007/s10460-024-10572-5)

28.

Pixova M, Plank C. Urban food governance without local food: missing links between Czech post-socialist cities and urban food alternatives. AGRICULTURE AND HUMAN VALUES. 2024. doi:[10.1007/s10460-024-10567-2](https://doi.org/10.1007/s10460-024-10567-2)

29.

Panghal A, Sindhu S, Sarao J, Dahiya S. Local Food Entrepreneurship in Rural India: Modelling the Challenges. International Journal of Rural Management. 2024;20: 65–84. doi:[10.1177/09730052231151796](https://doi.org/10.1177/09730052231151796)

30.

Osunmuyiwa OO, Sakariyawo OS, Peacock AD. Accelerating Farmers Move on the Irrigation Ladder Understanding the Potential of Solar-Powered Irrigation in Nigeria. Economic Trends and Sustainable Environmental Assessment. 2024; 37–56. doi:[10.1201/9781003306214-4](https://doi.org/10.1201/9781003306214-4)

31.

O’Neill K. Good food or industrial food: Discourses of the rural idyll and alternative food networks. Journal of Rural Studies. 2024;105. doi:[10.1016/j.jrurstud.2024.103199](https://doi.org/10.1016/j.jrurstud.2024.103199)

32.

O’Connor G, Reis K, Desha C, Burkett I. Valuing farmers in transitions to more sustainable food systems: A systematic literature review of local food producers’ experiences and contributions in short food supply chains. AGRICULTURE AND HUMAN VALUES. 2024. doi:[10.1007/s10460-024-10601-3](https://doi.org/10.1007/s10460-024-10601-3)

33.

Massengale SH, Hendrickson M. Testing and Expanding the Concept of Traditional and Contemporary Localism in Rural Local Food Systems with Ozark Wild Harvesters☆. Rural Sociology. 2024;89: 156–172. doi:[10.1111/ruso.12532](https://doi.org/10.1111/ruso.12532)

34.

Marín J, Garnatje T, Vallès J. Agro-Food Supply Chains in Peri-Urban Agricultural Areas: Do They Contribute to Preserve Local Biodiversity? The Case of Baix Llobregat Agrarian Park. Sustainability (Switzerland). 2024;16. doi:[10.3390/su16072882](https://doi.org/10.3390/su16072882)

35.

López-García D, Carrascosa-García M. Sustainable food policies without sustainable farming? Challenges for agroecology-oriented farmers in relation to urban (sustainable) food policies. Journal of Rural Studies. 2024;105. doi:[10.1016/j.jrurstud.2023.103160](https://doi.org/10.1016/j.jrurstud.2023.103160)

36.

Kujala S, Koppelmäki K. Regional economic assessment of a novel place-based model for sustainable food systems. Geography and Sustainability. 2024;5: 220–229. doi:[10.1016/j.geosus.2024.02.002](https://doi.org/10.1016/j.geosus.2024.02.002)

37.

Kraeger P, Phillips RG, Lubin JH, Weir J, Patterson K. Assessing Healthy Effects between Local Level Farmer’s Markets and Community-Supported Agriculture and Physical Well-Being at the State Level. Sustainability (Switzerland). 2024;16. doi:[10.3390/su16020867](https://doi.org/10.3390/su16020867)

38.

Kłoczko-Gajewska A, Malak-Rawlikowska A, Majewski E, Wilkinson A, Gorton M, Tocco B, et al. What are the economic impacts of short food supply chains? A local multiplier effect (LM3) evaluation. European Urban and Regional Studies. 2024;31: 281–301. doi:[10.1177/09697764231201572](https://doi.org/10.1177/09697764231201572)

39.

Guerrero Lara L, Feola G, Driessen P. Drawing boundaries: Negotiating a collective ‘we’ in community-supported agriculture networks. Journal of Rural Studies. 2024;106. doi:[10.1016/j.jrurstud.2024.103197](https://doi.org/10.1016/j.jrurstud.2024.103197)

40.

Garrity K, Krzyzanowski Guerra K, Hart H, Al-Muhanna K, Kunkler EC, Braun A, et al. Local Food System Approaches to Address Food and Nutrition Security among Low-Income Populations: A Systematic Review. Advances in Nutrition. 2024;15. doi:[10.1016/j.advnut.2023.100156](https://doi.org/10.1016/j.advnut.2023.100156)

41.

Flachs A, Raturi A, Low M, Miller V, Norton J, Redmond C, et al. Digital tools for local farmers: Thinking with spreadsheets in the wake of the COVID-19 pandemic. Culture, Agriculture, Food and Environment. 2024;46: 36–47. doi:[10.1111/cuag.12316](https://doi.org/10.1111/cuag.12316)

42.

Chavez-Miguel G, Hämmerle J, González A, Canetti C, Gleich P, Halfast RL, et al. Local food system resilience in the context of shocks and crises: vulnerabilities and responses of agroecology-based farmers in Peru, Germany, and the United States. Agroecology and Sustainable Food Systems. 2024;48: 876–897. doi:[10.1080/21683565.2024.2341987](https://doi.org/10.1080/21683565.2024.2341987)

43.

Cerf M, Le Bail C, Boccara V, Loyce C. Understanding and supporting intermediation work to address territorialized public policy issues: The case of a Territorial Food Project in France. WORK-A JOURNAL OF PREVENTION ASSESSMENT & REHABILITATION. 2024;77: 343–357. doi:[10.3233/WOR-220298](https://doi.org/10.3233/WOR-220298)

44.

Burton G, Gori B, Camara S, Ceci P, Conde N, Couch C, et al. Landrace diversity and heritage of the indigenous millet crop fonio (*Digitaria exilis*): Socio-cultural and climatic drivers of change in the Fouta Djallon region of Guinea. PLANTS PEOPLE PLANET. 2024. doi:[10.1002/ppp3.10490](https://doi.org/10.1002/ppp3.10490)

45.

Bi AZ, Umesh KB, Abdul BM, Sivakuma D, Srikanth P. Economic and environmental sustainability of agriculture production at the crop level. GLOBAL JOURNAL OF ENVIRONMENTAL SCIENCE AND MANAGEMENT-GJESM. 2024;10: 1433–1456. doi:[10.22034/gjesm.2024.03.29](https://doi.org/10.22034/gjesm.2024.03.29)

46.

Alberti N, Esposito G, Ferrando T. Organizing sustainable and fair agri-food systems: exploring the role of north-north alternative food networks in the European Union. AGROECOLOGY AND SUSTAINABLE FOOD SYSTEMS. 2024. doi:[10.1080/21683565.2024.2368122](https://doi.org/10.1080/21683565.2024.2368122)

47.

Parausic V, Kolasinac S, Muca (Dashi) E, Saric BB. Competencies of Western Balkan farmers for participating in short food supply chains: Honey case study. NEW MEDIT. 2023;22: 73–89. doi:[10.30682/nm2304e](https://doi.org/10.30682/nm2304e)

48.

Lamarque M, Tome-Martin P, Moro-Gutierrez L. Personal and community values behind sustainable food consumption: a meta-ethnography. FRONTIERS IN SUSTAINABLE FOOD SYSTEMS. 2023;7. doi:[10.3389/fsufs.2023.1292887](https://doi.org/10.3389/fsufs.2023.1292887)

49.

Bottazzi P, Seck SM, Niang M, Moser S. Beyond motivations: A framework unraveling the systemic barriers to organic farming adoption in northern Senegal. JOURNAL OF RURAL STUDIES. 2023;104. doi:[10.1016/j.jrurstud.2023.103158](https://doi.org/10.1016/j.jrurstud.2023.103158)

50.

Azima S, Mundler P. Farmer satisfaction and short food supply chains. AGRICULTURE AND HUMAN VALUES. 2023;40: 1531–1536. doi:[10.1007/s10460-023-10447-1](https://doi.org/10.1007/s10460-023-10447-1)

51.

Semakula HM, Liang S, Mukwaya PI, Mugagga F. Application of a Bayesian network modelling approach to predict the cascading effects of COVID-19 restrictions on the planting activities of smallholder farmers in Uganda. AGRICULTURAL SYSTEMS. 2023;211. doi:[10.1016/j.agsy.2023.103733](https://doi.org/10.1016/j.agsy.2023.103733)

52.

Paredes A. The political work of food delivery: consumer co-operative systems and women’s labor in “relationless” Japan. FOOD CULTURE & SOCIETY. 2023;26: 1174–1192. doi:[10.1080/15528014.2022.2113284](https://doi.org/10.1080/15528014.2022.2113284)

53.

Li L, Zhao H, Liu N. MCD-Yolov5: Accurate, Real-Time Crop Disease and Pest Identification Approach Using UAVs. ELECTRONICS. 2023;12. doi:[10.3390/electronics12204365](https://doi.org/10.3390/electronics12204365)

54.

Geissberger S, Chapman M. The Work that Work does: How intrinsic and instrumental values are transformed into relational values through active work participation in Swiss community supported agriculture. PEOPLE AND NATURE. 2023;5: 1649–1663. doi:[10.1002/pan3.10531](https://doi.org/10.1002/pan3.10531)

55.

Sharifi E, Fang L, Amin SH. A novel two-stage multi-objective optimization model for sustainable soybean supply chain design under uncertainty. SUSTAINABLE PRODUCTION AND CONSUMPTION. 2023;40: 297–317. doi:[10.1016/j.spc.2023.07.006](https://doi.org/10.1016/j.spc.2023.07.006)

56.

Guibrunet L, Rubio M, Flores Abreu IN. Reclaiming traditional food systems in alternative food networks. Insights from Mexico city peri-urban agriculture. LOCAL ENVIRONMENT. 2023;28: 1153–1172. doi:[10.1080/13549839.2023.2194618](https://doi.org/10.1080/13549839.2023.2194618)

57.

Zhang Y, Ridings C, Semenov A. What to post? Understanding engagement cultivation in microblogging with big data-driven theory building. INTERNATIONAL JOURNAL OF INFORMATION MANAGEMENT. 2023;71. doi:[10.1016/j.ijinfomgt.2022.102509](https://doi.org/10.1016/j.ijinfomgt.2022.102509)

58.

Vilalta-Perdomo E, Salinas-Navarro DE, Michel-Villarreal R, Garcia Bustamante R. Digitalization of the Logistics Process in Short Food Supply Chains. An online Viable System Model application during the COVID-19 pandemic. SYSTEMIC PRACTICE AND ACTION RESEARCH. 2023;36: 509–534. doi:[10.1007/s11213-022-09619-7](https://doi.org/10.1007/s11213-022-09619-7)

59.

Lopez-Garcia D, Carrascosa-Garcia M. Agroecology-oriented farmers’ groups. A missing level in the construction of agroecology-based local agri-food systems? AGROECOLOGY AND SUSTAINABLE FOOD SYSTEMS. 2023;47: 996–1022. doi:[10.1080/21683565.2023.2217095](https://doi.org/10.1080/21683565.2023.2217095)

60.

Sheisha H, Kaniewski D, Marriner N, Djamali M, Younes G, Chen Z, et al. Feeding the pyramid builders: Early agriculture at Giza in Egypt. QUATERNARY SCIENCE REVIEWS. 2023;312. doi:[10.1016/j.quascirev.2023.108172](https://doi.org/10.1016/j.quascirev.2023.108172)

61.

Rode EL, Rover OJ, Desconsi C. Agroecology and direct sale of organic food: a study of two marketing experiences in Santa Catarina, Brazil. DESENVOLVIMENTO E MEIO AMBIENTE. 2023;62: 29–46. doi:[10.5380/dma.v62i0.81710](https://doi.org/10.5380/dma.v62i0.81710)

62.

Marfurt F, Haller T, Bottazzi P. Green Agendas and White Markets: The Coloniality of Agroecology in Senegal. LAND. 2023;12. doi:[10.3390/land12071324](https://doi.org/10.3390/land12071324)

63.

Stehrenberger A, Schneider T. “At first, I was only a subscriber”: re-mediating food citizens’ solidarity practices through digital technologies. FRONTIERS IN SUSTAINABLE FOOD SYSTEMS. 2023;7. doi:[10.3389/fsufs.2023.1214354](https://doi.org/10.3389/fsufs.2023.1214354)

64.

MacKay C. Grass-Fed Beef, Alterity, and Care: Complicating food Binaries, Relations, and Practices. JOURNAL OF AGRICULTURAL & ENVIRONMENTAL ETHICS. 2023;36. doi:[10.1007/s10806-023-09906-w](https://doi.org/10.1007/s10806-023-09906-w)

65.

Boillat S, Bottazzi P, Sabaly IK. The division of work in Senegalese conventional and alternative food networks: a contributive justice perspective. FRONTIERS IN SUSTAINABLE FOOD SYSTEMS. 2023;7. doi:[10.3389/fsufs.2023.1127593](https://doi.org/10.3389/fsufs.2023.1127593)

66.

Retiere M, Darly S. School food policies and the transition of urban food systems in Brazil and France: insights from Sao Paulo and Greater Paris region case studies. FRONTIERS IN SUSTAINABLE FOOD SYSTEMS. 2023;7. doi:[10.3389/fsufs.2023.984207](https://doi.org/10.3389/fsufs.2023.984207)

67.

Rana JC, Bisht IS. Reviving Smallholder Hill Farming by Involving Rural Youth in Food System Transformation and Promoting Community-Based Agri-Ecotourism: A Case of Uttarakhand State in North-Western India. SUSTAINABILITY. 2023;15. doi:[10.3390/su15118816](https://doi.org/10.3390/su15118816)

68.

Pugas A da S, Rover OJ, Martinelli SS, Teodolino FC. A trajectory of social innovations for the direct purchase of organic food by food services: a case study in Florianópolis, Brazil. FRONTIERS IN SUSTAINABLE FOOD SYSTEMS. 2023;7. doi:[10.3389/fsufs.2023.1102891](https://doi.org/10.3389/fsufs.2023.1102891)

69.

Wisniewska-Paluszak J, Paluszak G, Fiore M, Coticchio A, Galati A, Lira J. Urban agriculture business models and value propositions: Mixed methods approach based on evidence from Polish and Italian case studies. LAND USE POLICY. 2023;127. doi:[10.1016/j.landusepol.2023.106562](https://doi.org/10.1016/j.landusepol.2023.106562)

70.

Bindi L, Belliggiano A. A Highly Condensed Social Fact: Food Citizenship, Individual Responsibility, and Social Commitment. SUSTAINABILITY. 2023;15. doi:[10.3390/su15086881](https://doi.org/10.3390/su15086881)

71.

Bezard M, Barlagne C, Diman J-L, Angeon V, Morin R, Ozier-Lafontaine H, et al. Co-designing innovative plantain cropping systems to support the diversity of agroecological pathways in Guadeloupe. AGRONOMY FOR SUSTAINABLE DEVELOPMENT. 2023;43. doi:[10.1007/s13593-023-00879-8](https://doi.org/10.1007/s13593-023-00879-8)

72.

Benedek Z. On the transformative potential of Hungarian local food-buying clubs. FRONTIERS IN SUSTAINABLE FOOD SYSTEMS. 2023;7. doi:[10.3389/fsufs.2023.1124877](https://doi.org/10.3389/fsufs.2023.1124877)

73.

Akram HW, Akhtar S, Ahmad A, Anwar I, Sulaiman MABA. Developing a Conceptual Framework Model for Effective Perishable Food Cold-Supply-Chain Management Based on Structured Literature Review. SUSTAINABILITY. 2023;15. doi:[10.3390/su15064907](https://doi.org/10.3390/su15064907)

74.

Speich C, Barth-Jaeggi T, Musard C, Havugimana C, Nwokoro C, Gakuba E, et al. Nutrition in City Ecosystems (NICE): Protocol of a multi-sectoral development project to improve food and nutrition security of secondary city populations in Bangladesh, Kenya and Rwanda. FRONTIERS IN PUBLIC HEALTH. 2023;11. doi:[10.3389/fpubh.2023.1081535](https://doi.org/10.3389/fpubh.2023.1081535)

75.

Balcom R, Abebe GK, Yiridoe EK, Hartt CM. Sustainable production and distribution practices in Atlantic Canadian short food supply chains: Explorative study. FRONTIERS IN SUSTAINABLE FOOD SYSTEMS. 2023;7. doi:[10.3389/fsufs.2023.1121006](https://doi.org/10.3389/fsufs.2023.1121006)

76.

Baumann S, Johnston J, Oleschuk M. How do producers imagine consumers? Connecting farm and fork through a cultural repertoire of consumer sovereignty. SOCIOLOGIA RURALIS. 2023;63: 178–199. doi:[10.1111/soru.12401](https://doi.org/10.1111/soru.12401)

77.

Young T, Cumming G, Kerns E, Hunter-Thomson K, Lu H, Manik-Perlman T, et al. Strategies for Increasing Participation of Diverse Consumers in a Community Seafood Program. Journal of Agricultural and Environmental Ethics. 2023;36. doi:[10.1007/s10806-023-09912-y](https://doi.org/10.1007/s10806-023-09912-y)

78.

Torquati B, Loce-Mandes F, Martino G. School Food Policy through a Project Financing. Italian Review of Agricultural Economics. 2023;78: 93–109. doi:[10.36253/rea-14983](https://doi.org/10.36253/rea-14983)

79.

Sulistyowati CA, Afiff SA, Baiquni M, Siscawati M. Challenges and potential solutions in developing community supported agriculture: a literature review. Agroecology and Sustainable Food Systems. 2023;47: 834–856. doi:[10.1080/21683565.2023.2187002](https://doi.org/10.1080/21683565.2023.2187002)

80.

Solarz K, Raftowicz M, Kachniarz M, Dradrach A. Back to Locality? Demand Potential Analysis for Short Food Supply Chains. International Journal of Environmental Research and Public Health. 2023;20. doi:[10.3390/ijerph20043641](https://doi.org/10.3390/ijerph20043641)

81.

Singh P. Pandemic, Resilience and Sustainability: Agroecology and Local Food System as the Way Forward. The Route Towards Global Sustainability: Challenges and Management Practices. 2023; 275–288. doi:[10.1007/978-3-031-10437-4_14](https://doi.org/10.1007/978-3-031-10437-4_14)

82.

Schreiber K, Winkler KJ, Abellon K, MacDonald GK. Planning the foodshed: Rural and peri-urban factors in local food strategies of major cities in Canada and the United States. Urban Agriculture and Regional Food Systems. 2023;8. doi:[10.1002/uar2.20041](https://doi.org/10.1002/uar2.20041)

83.

Schreiber K, Soubry B, Dove-McFalls C, MacDonald GK. Untangling the role of social relationships for overcoming challenges in local food systems: a case study of farmers in Québec, Canada. Agriculture and Human Values. 2023;40: 141–156. doi:[10.1007/s10460-022-10343-0](https://doi.org/10.1007/s10460-022-10343-0)

84.

Schilling F, Wahlen S, Domptail SE. The moral economy of community supported agriculture - hopes and troubles of farmers as community makers. Community Food Initiatives: A Critical Reparative Approach. 2023; 135–154. doi:[10.4324/9781003195085-10](https://doi.org/10.4324/9781003195085-10)

85.

Rozanski C, Gavin M. Growing in relation with the land: Experiential learning of Root and Regenerate Urban Farms. Journal of Agriculture, Food Systems, and Community Development. 2023;13. doi:[10.5304/jafscd.2023.131.002](https://doi.org/10.5304/jafscd.2023.131.002)

86.

Price MJ. Seeing Green: Lifecycles of an Arctic Agricultural Frontier☆. Rural Sociology. 2023;88: 941–971. doi:[10.1111/ruso.12506](https://doi.org/10.1111/ruso.12506)

87.

Popović V, Mihailović B. Women entrepreneurship in multifunctional agriculture for rural revival in Serbia. Sustainable Growth and Global Social Development in Competitive Economies. 2023; 225–247. doi:[10.4018/978-1-6684-8810-2.ch012](https://doi.org/10.4018/978-1-6684-8810-2.ch012)

88.

Obach R, Schusler T, Vaca P, Durkin S, Sheikh M. Connectivity and racial equity in responding to COVID-19 impacts in the Chicago regional food system. JOURNAL OF AGRICULTURE FOOD SYSTEMS AND COMMUNITY DEVELOPMENT. 2023;12: 305–320. doi:[10.5304/jafscd.2023.122.010](https://doi.org/10.5304/jafscd.2023.122.010)

89.

Mkhize X, Mthembu BE, Napier C. Transforming a local food system to address food and nutrition insecurity in an urban informal settlement area: A study in Umlazi Township in Durban, South Africa. Journal of Agriculture and Food Research. 2023;12. doi:[10.1016/j.jafr.2023.100565](https://doi.org/10.1016/j.jafr.2023.100565)

90.

Maysels R, Figueroa Casas A, Otero Sarmiento JD, Zuñiga Meneses SM. Conceptualization of alternative food networks in Latin America: a case study of a local food system in Southwestern Colombia. Frontiers in Sustainable Food Systems. 2023;7. doi:[10.3389/fsufs.2023.1216116](https://doi.org/10.3389/fsufs.2023.1216116)

91.

Levidow L, Sansolo D, Schiavinatto M. Territorialising Local Food Systems for an Agroecological Transition in Latin America. Land. 2023;12. doi:[10.3390/land12081577](https://doi.org/10.3390/land12081577)

92.

Lasco G, Gregory V, Compra JM, Leuangvilay P, Suphanchaimat R, Zhang Y, et al. Nutrition in Times of Crisis: A Qualitative Study in Siargao Island, Philippines, during the COVID-19 Pandemic. Acta Medica Philippina. 2023;57: 5–15. doi:[10.47895/amp.vi0.4963](https://doi.org/10.47895/amp.vi0.4963)

93.

Kelmenson S. Between the farm and the fork: job quality in sustainable food systems. Agriculture and Human Values. 2023;40: 317–358. doi:[10.1007/s10460-022-10362-x](https://doi.org/10.1007/s10460-022-10362-x)

94.

Kazak JK, Świąder M, Arciniegas G, Aslanoğlu R, Wascher D, Chrobak G. THE APPLICATION OF GEOPLANNER IN THE MANAGEMENT OF LOCAL DEVELOPMENT. Acta Scientiarum Polonorum, Administratio Locorum. 2023;22: 525–535. doi:[10.31648/aspal.9012](https://doi.org/10.31648/aspal.9012)

95.

Hernández PA. Enabling Conditions for Local Food Systems to Emerge in Predominately Rural Regions of Portugal—A Food Access Approach. Land. 2023;12. doi:[10.3390/land12020461](https://doi.org/10.3390/land12020461)

96.

Gromek-Broc K. The European green deal and regionalisation: Italian and polish case studies. Regional Approaches to the Energy Transition: A Multidisciplinary Perspective. 2023; 75–104. doi:[10.1007/978-3-031-19358-3_7](https://doi.org/10.1007/978-3-031-19358-3_7)

97.

Green A, Nemecek T, Mathys A. A proposed framework to develop nutrient profiling algorithms for assessments of sustainable food: the metrics and their assumptions matter. INTERNATIONAL JOURNAL OF LIFE CYCLE ASSESSMENT. 2023. doi:[10.1007/s11367-023-02210-9](https://doi.org/10.1007/s11367-023-02210-9)

98.

Egli L, Rüschhoff J, Priess J. A systematic review of the ecological, social and economic sustainability effects of community-supported agriculture. Frontiers in Sustainable Food Systems. 2023;7. doi:[10.3389/fsufs.2023.1136866](https://doi.org/10.3389/fsufs.2023.1136866)

99.

Dorr E, Goldstein B, Aubry C, Gabrielle B, Horvath A. Life cycle assessment of eight urban farms and community gardens in France and California. Resources, Conservation and Recycling. 2023;192. doi:[10.1016/j.resconrec.2023.106921](https://doi.org/10.1016/j.resconrec.2023.106921)

100.

Cirone F, Masotti M, Prosperi P, Bosi S, Dinelli G, Vittuari M. Business strategy pathways for short food supply chains: Sharing value between consumers and producers. Sustainable Production and Consumption. 2023;40: 458–470. doi:[10.1016/j.spc.2023.07.017](https://doi.org/10.1016/j.spc.2023.07.017)

101.

Charatsari C, Lioutas ED, Michailidis A, Aidonis D, De Rosa M, Partalidou M, et al. Facets of value emerging through the operation of short food supply chains. NJAS: Impact in Agricultural and Life Sciences. 2023;95. doi:[10.1080/27685241.2023.2236961](https://doi.org/10.1080/27685241.2023.2236961)

102.

Brady PJ, Kunkel K, Baltaci A, Gold A, Laska MN. Experiences of Food Pantry Stakeholders and Emergency Food Providers in Rural Minnesota Communities. Journal of Nutrition Education and Behavior. 2023;55: 710–720. doi:[10.1016/j.jneb.2023.07.009](https://doi.org/10.1016/j.jneb.2023.07.009)

103.

Atoloye AT, Bassarab K, Schouboe S, Misiaszek C, Harding J, Calancie L, et al. Developing a food system indicators database to facilitate local food systems assessments: Using a scoping review approach. Journal of Agriculture, Food Systems, and Community Development. 2023;13. doi:[10.5304/jafscd.2023.131.008](https://doi.org/10.5304/jafscd.2023.131.008)

104.

Swaminathan B, Palani S, Vairavasundaram S. Meta Learning-Based Dynamic Ensemble Model for Crop Selection. APPLIED ARTIFICIAL IN℡LIGENCE. 2022;36. doi:[10.1080/08839514.2022.2145646](https://doi.org/10.1080/08839514.2022.2145646)

105.

Nichols C, Janssen B, Beamer C, Ferring C. Pivoting is exhausting: A critical analysis of local food system resilience. JOURNAL OF RURAL STUDIES. 2022;96: 180–189. doi:[10.1016/j.jrurstud.2022.10.024](https://doi.org/10.1016/j.jrurstud.2022.10.024)

106.

Birtalan IL, Ferto I, Neulinger A, Racz J, Olah A. The wellbeing paradox in Hungarian local sustainable agriculture: a health psychology approach. BMC PUBLIC HEALTH. 2022;22. doi:[10.1186/s12889-022-14643-2](https://doi.org/10.1186/s12889-022-14643-2)

107.

Bellon-Maurel V, Lutton E, Bisquert P, Brossard L, Chambaron-Ginhac S, Labarthe P, et al. Digital revolution for the agroecological transition of food systems: A responsible research and innovation perspective. AGRICULTURAL SYSTEMS. 2022;203. doi:[10.1016/j.agsy.2022.103524](https://doi.org/10.1016/j.agsy.2022.103524)

108.

Marchant L, Campos J, Luco J, Ramirez C, Barrientos F, Carrasco B, et al. Potential of traditional Chilean blood-fleshed peach to support livelihood opportunities in local agriculture. FRONTIERS IN SUSTAINABLE FOOD SYSTEMS. 2022;6. doi:[10.3389/fsufs.2022.820811](https://doi.org/10.3389/fsufs.2022.820811)

109.

Pieroni A, Sulaiman N, Polesny Z, Soukand R. From *Sxex* to *Chorta*: The Adaptation of Maronite Foraging Customs to the Greek Ones in Kormakitis, Northern Cyprus. PLANTS-BASEL. 2022;11. doi:[10.3390/plants11202693](https://doi.org/10.3390/plants11202693)

110.

Leitheiser S, Horlings I, Franklin A, Trell E-M. Regeneration at a distance from the state: From radical imaginaries to alternative practices in Dutch farming. SOCIOLOGIA RURALIS. 2022;62: 699–725. doi:[10.1111/soru.12403](https://doi.org/10.1111/soru.12403)

111.

Guell C, Brown CR, Navunicagi OW, Iese V, Badrie N, Wairiu M, et al. Perspectives on strengthening local food systems in Small Island Developing States. FOOD SECURITY. 2022;14: 1227–1240. doi:[10.1007/s12571-022-01281-0](https://doi.org/10.1007/s12571-022-01281-0)

112.

Clark JK, Jablonski BBR. Managing across boundaries for coordinated local and regional food system policy. FOOD POLICY. 2022;112. doi:[10.1016/j.foodpol.2022.102312](https://doi.org/10.1016/j.foodpol.2022.102312)

113.

Little M, Sylvester O. Agroecological producers shortening food chains during Covid-19: opportunities and challenges in Costa Rica. AGRICULTURE AND HUMAN VALUES. 2022;39: 1133–1140. doi:[10.1007/s10460-022-10298-2](https://doi.org/10.1007/s10460-022-10298-2)

114.

Belda-Miquel S. Expanding Well-Being by Participating in Grassroots Innovations: Using the Capability Approach to Explore the Interest of Alternative Food Networks for Community Social Services. BRITISH JOURNAL OF SOCIAL WORK. 2022;52: 3618–3638. doi:[10.1093/bjsw/bcab267](https://doi.org/10.1093/bjsw/bcab267)

115.

Souza A, Fornazier A. Case Study of the School Feeding Program in Distrito Federal, Brazil: Building Quality in Short Food Supply Chains. SUSTAINABILITY. 2022;14. doi:[10.3390/su141610192](https://doi.org/10.3390/su141610192)

116.

Csordas A, Lengyel P, Fuzesi I. Who Prefers Regional Products? A Systematic Literature Review of Consumer Characteristics and Attitudes in Short Food Supply Chains. SUSTAINABILITY. 2022;14. doi:[10.3390/su14158990](https://doi.org/10.3390/su14158990)

117.

Wang M, Kumar V, Ruan X, Saad M, Garza-Reyes JA, Kumar A. Sustainability concerns on consumers’ attitude towards short food supply chains: an empirical investigation. OPERATIONS MANAGEMENT RESEARCH. 2022;15: 76–92. doi:[10.1007/s12063-021-00188-x](https://doi.org/10.1007/s12063-021-00188-x)

118.

Almuflih AS, Sharma J, Tyagi M, Bhardwaj A, Qureshi MRNM, Khan N. Leveraging the Dynamics of Food Supply Chains towards Avenues of Sustainability. SUSTAINABILITY. 2022;14. doi:[10.3390/su14126958](https://doi.org/10.3390/su14126958)

119.

Manderscheid M, Fiala V, Edwards F, Freyer B, Saeumel I. Let’s Do It Online⁈ Challenges and Lessons for Inclusive Virtual Participation. FRONTIERS IN SUSTAINABLE FOOD SYSTEMS. 2022;6. doi:[10.3389/fsufs.2022.732943](https://doi.org/10.3389/fsufs.2022.732943)

120.

Bezerra AGC, Soler Montiel M, Butto Zarzar AL, Garcia Roces I. Gender, agroecology and local markets at Sao Jose de Mipibu in Rio Grande do Norte, Brazil. AGROECOLOGY AND SUSTAINABLE FOOD SYSTEMS. 2022;46: 604–625. doi:[10.1080/21683565.2021.2025193](https://doi.org/10.1080/21683565.2021.2025193)

121.

Parrot L, Biard Y, Klaver D, Kabre E, Vanniere H. Slicing the fruit five ways: An economic, social, and environmental assessment of five mango food supply chains in Burkina Faso. SUSTAINABLE PRODUCTION AND CONSUMPTION. 2022;30: 1032–1043. doi:[10.1016/j.spc.2022.01.019](https://doi.org/10.1016/j.spc.2022.01.019)

122.

Hardman M, Clark A, Sherriff G. Mainstreaming Urban Agriculture: Opportunities and Barriers to Upscaling City Farming. AGRONOMY-BASEL. 2022;12. doi:[10.3390/agronomy12030601](https://doi.org/10.3390/agronomy12030601)

123.

Ghosh-Jerath S, Kapoor R, Dhasmana A, Singh A, Downs S, Ahmed S. Effect of COVID-19 Pandemic on Food Systems and Determinants of Resilience in Indigenous Communities of Jharkhand State, India: A Serial Cross-Sectional Study. FRONTIERS IN SUSTAINABLE FOOD SYSTEMS. 2022;6. doi:[10.3389/fsufs.2022.724321](https://doi.org/10.3389/fsufs.2022.724321)

124.

Mahroof K, Omar A, Kucukaltan B. Sustainable food supply chains: overcoming key challenges through digital technologies. INTERNATIONAL JOURNAL OF PRODUCTIVITY AND PERFORMANCE MANAGEMENT. 2022;71: 981–1003. doi:[10.1108/IJPPM-12-2020-0687](https://doi.org/10.1108/IJPPM-12-2020-0687)

125.

Font RJR, Perez MD, Chirino PRB. System for the visualization of food production indicators through the SAEN+C Pinar Observatory. REVISTA COOPERATIVISMO Y DESARROLLO-COODES. 2022;10: 176–202.

126.

Thant PS, Espino A, Soria G, Myae C, Rodriguez E, Barbon WJ, et al. Myanmar local food systems in a changing climate: Insights from multiple stakeholders. Environmental and Sustainability Indicators. 2022;14. doi:[10.1016/j.indic.2022.100170](https://doi.org/10.1016/j.indic.2022.100170)

127.

Scott MK, Gutuskey L, Zwemer T, Gallington K. Farmers Market Food Navigator Program: Key Stakeholder Perceptions and Program Outcomes. Health Promotion Practice. 2022;23: 166–173. doi:[10.1177/1524839920978163](https://doi.org/10.1177/1524839920978163)

128.

Schreiber K, Soubry B, Dove-McFalls C, MacDonald GK. Diverse adaptation strategies helped local food producers cope with initial challenges of the Covid-19 pandemic: Lessons from Québec, Canada. Journal of Rural Studies. 2022;90: 124–133. doi:[10.1016/j.jrurstud.2022.02.002](https://doi.org/10.1016/j.jrurstud.2022.02.002)

129.

Prišenk J, Turk J. Assessment of Concept between Rural Development Challenges and Local Food Systems: A Combination between Multi-Criteria Decision Analysis and Econometric Modelling Approach. Sustainability (Switzerland). 2022;14. doi:[10.3390/su14063477](https://doi.org/10.3390/su14063477)

130.

Pereira LM, Kushitor SB, Cramer C, Drimie S, Isaacs M, Malgas R, et al. Leveraging the potential of wild food for healthy, sustainable, and equitable local food systems: learning from a transformation lab in the Western Cape region. Sustainability Science. 2022. doi:[10.1007/s11625-022-01182-3](https://doi.org/10.1007/s11625-022-01182-3)

131.

Nurhidayah L, Djalante R. Government Responses to COVID-19 and Their Implications on Food Security in Indonesia. Global Pandemic and Human Security: Technology and Development Perspective. 2022; 323–339. doi:[10.1007/978-981-16-5074-1_18](https://doi.org/10.1007/978-981-16-5074-1_18)

132.

Morsel N, Garambois N. Agroecology in the Limousin Mountains: Relocating and Diversifying Food Production to Encourage Employment and Conserve Semi-Natural Spaces. Revue de Geographie Alpine. 2022;110. doi:[10.4000/rga.10633](https://doi.org/10.4000/rga.10633)

133.

Mercier A-P, Rochefort G, Fortier J, Parent G, Provencher V, Lemieux S, et al. Development and Validation of a Short Questionnaire Assessing the Behavior of Local Food Procurement in Quebec, Canada. Current Developments in Nutrition. 2022;6. doi:[10.1093/cdn/nzac097](https://doi.org/10.1093/cdn/nzac097)

134.

Martinez CL, Rosero D, Thomas T, Soto Mas F. Community Supported Agriculture, Human Capital, and Community Health. Health Promotion Practice. 2022;23: 407–415. doi:[10.1177/15248399211070546](https://doi.org/10.1177/15248399211070546)

135.

Marrero A, Mattei J. Reclaiming traditional, plant-based, climate-resilient food systems in small islands. The Lancet Planetary Health. 2022;6: e171–e179. doi:[10.1016/S2542-5196(21)00322-3](https://doi.org/10.1016/S2542-5196(21)00322-3)

136.

Lofton S, Kersten M, Lubimbi N, Odoms-Young A. How community capacity building in urban agriculture can improve food access in predominantly Black communities. Journal of Community Practice. 2022;30: 395–417. doi:[10.1080/10705422.2022.2138667](https://doi.org/10.1080/10705422.2022.2138667)

137.

Li X. The “Real” Organic Food in China: The Tradition-Modern Divide and the Role of Boundary Work. International Journal of Sociology of Agriculture and Food. 2022;28: 43–57. doi:[10.48416/ijsaf.v28i1.71](https://doi.org/10.48416/ijsaf.v28i1.71)

138.

Koempel A, Brislen L, Jacobsen K, Clouser J, Vundi N, Li J, et al. Growing health: Building partnerships in healthcare and food systems for improved food access in Appalachia. Journal of Agriculture, Food Systems, and Community Development. 2022;11: 261–271. doi:[10.5304/jafscd.2022.114.022](https://doi.org/10.5304/jafscd.2022.114.022)

139.

Kimiywe J, Craig H, Agyapong A, Thorne-Lyman A, Matsisa P, Kiige L, et al. Diets of infants and young children in two counties of Kenya: Key drivers and barriers to improvement. MATERNAL AND CHILD NUTRITION. 2022. doi:[10.1111/mcn.13334](https://doi.org/10.1111/mcn.13334)

140.

Galeano-Barrera CJ, Mendoza-García EM, Martínez-Amariz AD, Romero-Riaño E. Theoretical model of territorial agro-industrial development through multi-focus research analytics. Journal of Rural Studies. 2022;94: 295–304. doi:[10.1016/j.jrurstud.2022.06.014](https://doi.org/10.1016/j.jrurstud.2022.06.014)

141.

Freedman DA, Clark JK, Lounsbury DW, Boswell L, Burns M, Jackson MB, et al. Food system dynamics structuring nutrition equity in racialized urban neighborhoods. American Journal of Clinical Nutrition. 2022;115: 1027–1038. doi:[10.1093/ajcn/nqab380](https://doi.org/10.1093/ajcn/nqab380)

142.

Florick L, Park CH. A pilot study exploring the impacts of COVID-19 on small-scale direct-marketing farmers in Northwest Arkansas and their responses to the pandemic. Journal of Agriculture, Food Systems, and Community Development. 2022;12: 47–61. doi:[10.5304/jafscd.2022.121.006](https://doi.org/10.5304/jafscd.2022.121.006)

143.

Errickson LB, Schoolman ED, Quick V, Davis S, Capece A. Engaging Farmers, Culinary Schools, and Communities in Value-Added Production to Strengthen Local Food Systems. Journal of Extension. 2022;60. doi:[10.34068/joe.60.03.04](https://doi.org/10.34068/joe.60.03.04)

144.

Brown KA, Venkateshmurthy NS, Potubariki G, Sharma P, Cardwell JM, Prabhakaran D, et al. The role of dairy in healthy and sustainable food systems: community voices from India. BMC Public Health. 2022;22. doi:[10.1186/s12889-022-13194-w](https://doi.org/10.1186/s12889-022-13194-w)

145.

Blom CDB, Steegeman P, Voss C, Sonneveld BGJS. Food in the cold: exploring food security and sovereignty in Whitehorse, Yukon. International Journal of Circumpolar Health. 2022;81. doi:[10.1080/22423982.2022.2025992](https://doi.org/10.1080/22423982.2022.2025992)

146.

Azima S, Mundler P. The gendered motives and experiences of Canadian women farmers in short food supply chains: Work satisfaction, values of care, and the potential for empowerment. Journal of Rural Studies. 2022;96: 19–31. doi:[10.1016/j.jrurstud.2022.10.007](https://doi.org/10.1016/j.jrurstud.2022.10.007)

147.

Azima S, Mundler P. Does direct farm marketing fulfill its promises? analyzing job satisfaction among direct-market farmers in Canada. Agriculture and Human Values. 2022;39: 791–807. doi:[10.1007/s10460-021-10289-9](https://doi.org/10.1007/s10460-021-10289-9)

148.

Apaliya MT, Kwaw E, Osae R, Alolga RN, Aikins ASS, Otoo GS, et al. THE IMPACT OF COVID-19 ON FOOD SECURITY: GHANA IN REVIEW. Journal of Food Technology Research. 2022;9: 160–175. doi:[10.18488/jftr.v9i3.3228](https://doi.org/10.18488/jftr.v9i3.3228)

149.

Aouinaït C, Christen D, Carlen C, Massri C, Reipurth M, Hieke S, et al. Barriers and Facilitators of Purchasing from Short Food Supply Chains in Europe: Insights from a Stakeholder Perspective. International Journal of Food Studies. 2022;11: 196–207. doi:[10.7455/ijfs/11.SI.2022.a6](https://doi.org/10.7455/ijfs/11.SI.2022.a6)

150.

Hoang V. Modern Short Food Supply Chain, Good Agricultural Practices, and Sustainability: A Conceptual Framework and Case Study in Vietnam. AGRONOMY-BASEL. 2021;11. doi:[10.3390/agronomy11122408](https://doi.org/10.3390/agronomy11122408)

151.

Burke A. The Crossroads of Ecotourism Dependency, Food Security and a Global Pandemic in Galapagos, Ecuador. SUSTAINABILITY. 2021;13. doi:[10.3390/su132313094](https://doi.org/10.3390/su132313094)

152.

Anderson JD, Mitchell JL, Maples JG. INVITED REVIEW: Lessons from the COVID-19 pandemic for food supply chains. APPLIED ANIMAL SCIENCE. 2021;37: 738–747. doi:[10.15232/aas.2021-02223](https://doi.org/10.15232/aas.2021-02223)

153.

Bastian GE, Buro D, Palmer-Keenan DM. Recommendations for Integrating Evidence-Based, Sustainable Diet Information into Nutrition Education. NUTRIENTS. 2021;13. doi:[10.3390/nu13114170](https://doi.org/10.3390/nu13114170)

154.

Hervas A. Mapping oil palm-related land use change in Guatemala, 2003-2019: Implications for food security. LAND USE POLICY. 2021;109. doi:[10.1016/j.landusepol.2021.105657](https://doi.org/10.1016/j.landusepol.2021.105657)

155.

Foti VT, Timpanaro G. Relationships, sustainability and agri-food purchasing behaviour in farmer markets in Italy. BRITISH FOOD JOURNAL. 2021;123: 428–453. doi:[10.1108/BFJ-04-2021-0358](https://doi.org/10.1108/BFJ-04-2021-0358)

156.

Pretorius B, Ambuko J, Papargyropoulou E, Schonfeldt HC. Guiding Nutritious Food Choices and Diets along Food Systems. SUSTAINABILITY. 2021;13. doi:[10.3390/su13179501](https://doi.org/10.3390/su13179501)

157.

Medici M, Canavari M, Castellini A. Exploring the economic, social, and environmental dimensions of community-supported agriculture in Italy. JOURNAL OF CLEANER PRODUCTION. 2021;316. doi:[10.1016/j.jclepro.2021.128233](https://doi.org/10.1016/j.jclepro.2021.128233)

158.

Papacharalampous N. A new rural in the city: A no-middlemen markets’ ethnography. JOURNAL OF RURAL STUDIES. 2021;86: 702–710. doi:[10.1016/j.jrurstud.2021.06.002](https://doi.org/10.1016/j.jrurstud.2021.06.002)

159.

Cimino O, Vassallo M, Henke R, Vanni F. Income Diversification Strategies of Italian Peri-Urban Farms: A Structural Equation Modeling Approach. LAND. 2021;10. doi:[10.3390/land10080790](https://doi.org/10.3390/land10080790)

160.

Blue Bird Jernigan V, Maudrie TL, Nikolaus CJ, Benally T, Johnson S, Teague T, et al. Food Sovereignty Indicators for Indigenous Community Capacity Building and Health. FRONTIERS IN SUSTAINABLE FOOD SYSTEMS. 2021;5. doi:[10.3389/fsufs.2021.704750](https://doi.org/10.3389/fsufs.2021.704750)

161.

Le Velly R, Goulet F, Vinck D. Allowing for detachment processes in market innovation. The case of short food supply chains. CONSUMPTION MARKETS & CULTURE. 2021;24: 313–328. doi:[10.1080/10253866.2020.1807342](https://doi.org/10.1080/10253866.2020.1807342)

162.

Rivera-Ferre MG, Lopez-i-Gelats F, Ravera F, Oteros-Rozas E, di Masso M, Binimelis R, et al. The two-way relationship between food systems and the COVID19 pandemic: causes and consequences. AGRICULTURAL SYSTEMS. 2021;191. doi:[10.1016/j.agsy.2021.103134](https://doi.org/10.1016/j.agsy.2021.103134)

163.

Nchanji EB, Lutomia CK. Regional impact of COVID-19 on the production and food security of common bean smallholder farmers in Sub-Saharan Africa: Implication for SDG’s. GLOBAL FOOD SECURITY-AGRICULTURE POLICY ECONOMICS AND ENVIRONMENT. 2021;29. doi:[10.1016/j.gfs.2021.100524](https://doi.org/10.1016/j.gfs.2021.100524)

164.

Galati A, Giacomarra M, Concialdi P, Crescimanno M. Exploring the feasibility of introducing electric freight vehicles in the short food supply chain: A multi-stakeholder approach. CASE STUDIES ON TRANSPORT POLICY. 2021;9: 950–957. doi:[10.1016/j.cstp.2021.04.015](https://doi.org/10.1016/j.cstp.2021.04.015)

165.

de Oliveira IK, de Oliveira LK, Amorim Faria Lisboa MR, Nunes Madalon EC, de Freitas LF, Peres Filho AC. The Geographical Distance between Producers and Consumers of the Organic Street Markets: The Case of Belo Horizonte, Brazil. LOGISTICS-BASEL. 2021;5. doi:[10.3390/logistics5020030](https://doi.org/10.3390/logistics5020030)

166.

Bottazzi P, Boillat S. Political Agroecology in Senegal: Historicity and Repertoires of Collective Actions of an Emerging Social Movement. SUSTAINABILITY. 2021;13. doi:[10.3390/su13116352](https://doi.org/10.3390/su13116352)

167.

Biazoti AR, Nakamura AC, Nagib G, Leao VOP de S, Giacche G, Mauad T. The Impact of COVID-19 on Urban Agriculture in Sao Paulo, Brazil. SUSTAINABILITY. 2021;13. doi:[10.3390/su13116185](https://doi.org/10.3390/su13116185)

168.

Raridon A, Mix TL, Einwohner RL. “Workarounds and Roadblocks”: Risk and Resistance among Food Movement Activists. SOCIAL CURRENTS. 2021;8: 182–198. doi:[10.1177/2329496520965627](https://doi.org/10.1177/2329496520965627)

169.

Kallas Z, Alba MF, Casellas K, Berges M, Degreef G, Gil JM. The development of short food supply chain for locally produced honey Understanding consumers’ opinions and willingness to pay in Argentina. BRITISH FOOD JOURNAL. 2021;123: 1664–1680. doi:[10.1108/BFJ-01-2019-0070](https://doi.org/10.1108/BFJ-01-2019-0070)

170.

Drottberger A, Melin M, Lundgren L. Alternative Food Networks in Food System Transition-Values, Motivation, and Capacity Building among Young Swedish Market Gardeners. SUSTAINABILITY. 2021;13. doi:[10.3390/su13084502](https://doi.org/10.3390/su13084502)

171.

Cristiano S. Organic vegetables from community-supported agriculture in Italy: Emergy assessment and potential for sustainable, just, and resilient urban-rural local food production. JOURNAL OF CLEANER PRODUCTION. 2021;292. doi:[10.1016/j.jclepro.2021.126015](https://doi.org/10.1016/j.jclepro.2021.126015)

172.

Escobar-Lopez SY, Amaya-Corchuelo S, Espinoza-Ortega A. Alternative Food Networks: Perceptions in Short Food Supply Chains in Spain. SUSTAINABILITY. 2021;13. doi:[10.3390/su13052578](https://doi.org/10.3390/su13052578)

173.

Mejia G, Granados-Rivera D, Jarrin JA, Castellanos A, Mayorquin N, Molano E. Strategic Supply Chain Planning for Food Hubs in Central Colombia: An Approach for Sustainable Food Supply and Distribution. APPLIED SCIENCES-BASEL. 2021;11. doi:[10.3390/app11041792](https://doi.org/10.3390/app11041792)

174.

Occelli M, Mantino A, Ragaglini G, Dell’Acqua M, Fadda C, Pe ME, et al. Traditional knowledge affects soil management ability of smallholder farmers in marginal areas. AGRONOMY FOR SUSTAINABLE DEVELOPMENT. 2021;41. doi:[10.1007/s13593-020-00664-x](https://doi.org/10.1007/s13593-020-00664-x)

175.

Levidow L, Sansolo D, Schiavinatto M. Agroecological innovation constructing socionatural order for social transformation: two case studies in Brazil. TAPUYA-LATIN AMERICAN SCIENCE TECHNOLOGY AND SOCIETY. 2021;4. doi:[10.1080/25729861.2020.1843318](https://doi.org/10.1080/25729861.2020.1843318)

176.

Zoller HM. Re-Imagining Localism and Food Justice: Co-Op Cincy and the Union Cooperative Movement. Frontiers in Communication. 2021;6. doi:[10.3389/fcomm.2021.686400](https://doi.org/10.3389/fcomm.2021.686400)

177.

Vatta K, Bhogal S, Petrie CA, Greens AS, Dixit S. Impact of COVID-19 Lockdown on Punjab Agriculture. Covid-19 Pandemic and Economic Development: Emerging Public Policy Lessons for Indian Punjab. 2021; 33–47. doi:[10.1007/978-981-16-4442-9_3](https://doi.org/10.1007/978-981-16-4442-9_3)

178.

Saediman H, Gafaruddin A, Hidrawati H, Salam I, Ulimaz A, Sarimustaqiyma Rianse I, et al. The contribution of home food gardening program to household food security in indonesia: A review. WSEAS Transactions on Environment and Development. 2021;17: 795–809. doi:[10.37394/232015.2021.17.75](https://doi.org/10.37394/232015.2021.17.75)

179.

Rodríguez-García MÁ, García-Sánchez F, Valencia-García R. Knowledge-based system for crop pests and diseases recognition. Electronics (Switzerland). 2021;10. doi:[10.3390/electronics10080905](https://doi.org/10.3390/electronics10080905)

180.

Reese AM, Cooper D. Making Spaces Something Like Freedom: Black Feminist Praxis In The Re/Imagining Of A Just Food System. ACME. 2021;20: 450–459.

181.

Radcliffe J, Skinner K, Spring A, Picard L, Benoit F, Dodd W. Virtual barriers: unpacking the sustainability implications of online food spaces and the Yellowknife Farmers Market’s response to COVID-19. Nutrition Journal. 2021;20. doi:[10.1186/s12937-021-00664-x](https://doi.org/10.1186/s12937-021-00664-x)

182.

Preininger EM, Hafner R. I have a garden on the Internet! Searching for the farmer in a remotely controlled farming enterprise. Geographica Helvetica. 2021;76: 249–260. doi:[10.5194/gh-76-249-2021](https://doi.org/10.5194/gh-76-249-2021)

183.

Plakias ZT. Cost-benefit analysis as a tool for measuring economic impacts of local food systems: Case study of an institutional sourcing change. Journal of Agriculture, Food Systems, and Community Development. 2021;10: 161–185. doi:[10.5304/jafscd.2021.103.011](https://doi.org/10.5304/jafscd.2021.103.011)

184.

Paciarotti C, Torregiani F. The logistics of the short food supply chain: A literature review. Sustainable Production and Consumption. 2021;26: 428–442. doi:[10.1016/j.spc.2020.10.002](https://doi.org/10.1016/j.spc.2020.10.002)

185.

Naglis-Liepa K, Proškina L, Paula L, Kaufmane D. Modelling the multiplier effect of a local food system. Agronomy Research. 2021;19: 1075–1086. doi:[10.15159/AR.21.072](https://doi.org/10.15159/AR.21.072)

186.

Miranda DLR, Escosteguy IL, Rover OJ, Sampaio CAC. Organic markets social construction: the case of Responsible Consumer Cells in Florianópolis-SC. Revista de Economia e Sociologia Rural. 2021;59: 1–14. doi:[10.1590/1806-9479.2021.220071](https://doi.org/10.1590/1806-9479.2021.220071)

187.

McDaniel T, Soto Mas F, Sussman AL. Growing Connections: Local Food Systems and Community Resilience. Society and Natural Resources. 2021;34: 1375–1393. doi:[10.1080/08941920.2021.1958965](https://doi.org/10.1080/08941920.2021.1958965)

188.

Marat-Mendes T, Isidoro I, Catela J, Pereira M, Borges J, Lopes SS, et al. Drivers of change: How the food system of the Lisbon Metropolitan Area is being shaped by activities, initiatives and citizens needs towards a sustainable transition. Cidades. 2021; 41–62. doi:[10.15847/CCT.20490](https://doi.org/10.15847/CCT.20490)

189.

Lovatto AB, Miranda DLR, Rover OJ, Neto AB. Relationship and loyalty between farmers and consumers in direct selling groups of agroecological foods in Florianopolis-SC. Revista de Economia e Sociologia Rural. 2021;59: 1–14. doi:[10.1590/1806-9479.2021.227676](https://doi.org/10.1590/1806-9479.2021.227676)

190.

Kondo C. Re-energizing Japan’s *teikei* movement: Understanding intergenerational transitions of diverse economies. JOURNAL OF AGRICULTURE FOOD SYSTEMS AND COMMUNITY DEVELOPMENT. 2021;10: 103–121. doi:[10.5304/jafscd.2021.104.0031](https://doi.org/10.5304/jafscd.2021.104.0031)

191.

Klimek M, Bingen J, Freyer B, Paxton R. From schnitzel to sustainability: Shifting values at Vienna’s urban farmers markets. Sustainability (Switzerland). 2021;13. doi:[10.3390/su13158327](https://doi.org/10.3390/su13158327)

192.

Havran D, Kerényi P, Víg AA. Social finance and agricultural funding. Innovations in Social Finance: Transitioning Beyond Economic Value. 2021; 269–290. doi:[10.1007/978-3-030-72535-8_13](https://doi.org/10.1007/978-3-030-72535-8_13)

193.

Harden N, Bertsch B, Carlson K, Myrdal M, Bobicic I, Gold A, et al. Cass Clay Food Partners: A networked response to COVID-19. Journal of Agriculture, Food Systems, and Community Development. 2021;10: 181–196. doi:[10.5304/jafscd.2021.102.050](https://doi.org/10.5304/jafscd.2021.102.050)

194.

Gallegos-Riofrío CA, Waters WF, Carrasco A, Riofrío LA, Pintag M, Caranqui M, et al. Caliata: An Indigenous Community in Ecuador Offers Lessons on Food Sovereignty and Sustainable Diets. Current Developments in Nutrition. 2021;5: 61–73. doi:[10.1093/cdn/nzab009](https://doi.org/10.1093/cdn/nzab009)

195.

Dharmalingam B, Giri Nandagopal MS, Thulasiraman V, Kothakota A. Short food supply chains to resolve food scarcity during COVID-19 pandemic—An Indian model. Advances in Food Security and Sustainability. 2021;6: 35–63. doi:[10.1016/bs.af2s.2021.08.001](https://doi.org/10.1016/bs.af2s.2021.08.001)

196.

Costello C, Oveysi Z, Dundar B, McGarvey R. Assessment of the effect of urban agriculture on achieving a localized food system centered on chicago, il using robust optimization. Environmental Science and Technology. 2021;55: 2684–2694. doi:[10.1021/acs.est.0c04118](https://doi.org/10.1021/acs.est.0c04118)

197.

Cengız AE, Özdemır ZB. Agricultural Landscape Planning on the Basis of Conservation and Sustainable Use of Landscapes. Theories, Techniques, Strategies For Spatial Planners and Designers: Planning, Design, Applications. 2021; 725–746.

198.

Carvalho Verano TD, Figueiredo RS, Silva Medina GD. Family farmers in short food supply chains: a quantitative assessment of farmers’ markets. Revista de Economia e Sociologia Rural. 2021;59: 1–17. doi:[10.1590/1806-9479.2021.228830](https://doi.org/10.1590/1806-9479.2021.228830)

199.

Canal Vieira L, Serrao-Neumann S, Howes M. Daring to build fair and sustainable urban food systems: A case study of alternative food networks in Australia. Agroecology and Sustainable Food Systems. 2021;45: 344–365. doi:[10.1080/21683565.2020.1812788](https://doi.org/10.1080/21683565.2020.1812788)

200.

Campos ASN, Satolo EG, Mac-Lean PAB, Júnior SSB. Economic sustainability analysis of the specialty coffee farmers in Garça/SP. Coffee Science. 2021;16. doi:[10.25186/.V16I.1993](https://doi.org/10.25186/.V16I.1993)

201.

Camara SB, Andreatta T. Socioeconomic reproduction of farmers from the extreme north of rio grande do sul-br inserted in short food supply chains (SFSCS). Revista Brasileira de Gestao e Desenvolvimento Regional. 2021;17: 33–47.

202.

Brune S, Knollenberg W, Stevenson KT, Barbieri C, Schroeder-Moreno M. The Influence of Agritourism Experiences on Consumer Behavior toward Local Food. Journal of Travel Research. 2021;60: 1318–1332. doi:[10.1177/0047287520938869](https://doi.org/10.1177/0047287520938869)

203.

Bottazzi P, Boillat S. Agroecological Farmer Movements and Advocacy Coalitions in Sub-Saharan Africa: Between De-Politicization and Re-Politicization. The Palgrave Handbook of Environmental Labour Studies. 2021; 415–440. doi:[10.1007/978-3-030-71909-8_18](https://doi.org/10.1007/978-3-030-71909-8_18)

204.

Benedek Z, Fertö I, Marreiros CG, De Aguiar PM, Pocol CB, Čechura L, et al. Farm diversification as a potential success factor for small-scale farmers constrained by COVID-related lockdown. Contributions from a survey conducted in four European countries during the first wave of COVID-19. PLoS ONE. 2021;16. doi:[10.1371/journal.pone.0251715](https://doi.org/10.1371/journal.pone.0251715)

205.

Belda-Miquel S, Ruiz-Molina E, Gil-Saura I. Social Innovation for Sustainability and the Common Good in Ecosystems of the Fourth Sector: The Case of Distribution Through Alternative Food Networks in Valencia (Spain). Studies on Entrepreneurship, Structural Change and Industrial Dynamics. 2021; 141–167. doi:[10.1007/978-3-030-68390-0_8](https://doi.org/10.1007/978-3-030-68390-0_8)

206.

Bachman GH, Lupolt SN, Strauss M, Kennedy RD, Nachman KE. An examination of adaptations of direct marketing channels and practices by Maryland fruit and vegetable farmers during the COVID-19 pandemic. JOURNAL OF AGRICULTURE FOOD SYSTEMS AND COMMUNITY DEVELOPMENT. 2021;10: 283–301. doi:[10.5304/jafscd.2021.104.010](https://doi.org/10.5304/jafscd.2021.104.010)

207.

Atalan-Helicke N, Abiral B. Alternative food distribution networks, resilience, and urban food security in Turkey during the COVID-19 pandemic. JOURNAL OF AGRICULTURE FOOD SYSTEMS AND COMMUNITY DEVELOPMENT. 2021;10: 89–104. doi:[10.5304/jafscd.2021.102.021](https://doi.org/10.5304/jafscd.2021.102.021)

208.

Ammons S, Blacklin S, Bloom D, Brown S, Cappellazzi M, Creamer N, et al. A collaborative approach to COVID-19 response: The Center for Environmental Farming Systems community-based food system initiatives. Journal of Agriculture, Food Systems, and Community Development. 2021;10: 297–302. doi:[10.5304/jafscd.2021.102.004](https://doi.org/10.5304/jafscd.2021.102.004)

209.

Allaby M, MacDonald GK, Turner S. Growing pains: Small-scale farmer responses to an urban rooftop farming and online marketplace enterprise in Montréal, Canada. Agriculture and Human Values. 2021;38: 677–692. doi:[10.1007/s10460-020-10173-y](https://doi.org/10.1007/s10460-020-10173-y)

210.

Ajayi O, Ekanem E, Mafuyai M. Economic contributions of the local food systems in tennessee. Journal of Food Distribution Research. 2021;52: 8–17.

211.

Zwart TA, Mathijs E. Exploring emergent practices in Alternative Food Networks: Voedselteams in Belgium. JOURNAL OF RURAL STUDIES. 2020;80: 586–594. doi:[10.1016/j.jrurstud.2020.10.049](https://doi.org/10.1016/j.jrurstud.2020.10.049)

212.

Zhang L. From left behind to leader: gender, agency, and food sovereignty in China. AGRICULTURE AND HUMAN VALUES. 2020;37: 1111–1123. doi:[10.1007/s10460-020-10114-9](https://doi.org/10.1007/s10460-020-10114-9)

213.

Vincent O, Feola G. A framework for recognizing diversity beyond capitalism in agri-food systems. JOURNAL OF RURAL STUDIES. 2020;80: 302–313. doi:[10.1016/j.jrurstud.2020.10.002](https://doi.org/10.1016/j.jrurstud.2020.10.002)

214.

Sitaker M, Kolodinsky J, Wang W, Chase LC, Kim JVS, Smith D, et al. Evaluation of Farm Fresh Food Boxes: A Hybrid Alternative Food Network Market Innovation. SUSTAINABILITY. 2020;12. doi:[10.3390/su122410406](https://doi.org/10.3390/su122410406)

215.

Mundler P, Jean-Gagnon J. Short food supply chains, labor productivity and fair earnings: an impossible equation? RENEWABLE AGRICULTURE AND FOOD SYSTEMS. 2020;35: 697–709. doi:[10.1017/S1742170519000358](https://doi.org/10.1017/S1742170519000358)

216.

Mert-Cakal T, Miele M. `Workable utopias’ for social change through inclusion and empowerment? Community supported agriculture (CSA) in Wales as social innovation. AGRICULTURE AND HUMAN VALUES. 2020;37: 1241–1260. doi:[10.1007/s10460-020-10141-6](https://doi.org/10.1007/s10460-020-10141-6)

217.

Cardarelli K, DeWitt E, Gillespie R, Norman-Burgdolf H, Jones N, Mullins JT. “We’re, Like, the Most Unhealthy People in the Country”: Using an Equity Lens to Reduce Barriers to Healthy Food Access in Rural Appalachia. PREVENTING CHRONIC DISEASE. 2020;17. doi:[10.5888/pcd17.200340](https://doi.org/10.5888/pcd17.200340)

218.

Boz Z, Koelsch Sand C. A systematic analysis of the overall nutritional contribution of food loss and waste in tomatoes, spinach, and kidney beans as a function of processing. JOURNAL OF FOOD PROCESS ENGINEERING. 2020;43. doi:[10.1111/jfpe.13509](https://doi.org/10.1111/jfpe.13509)

219.

da Cunha MA, Arlego Paraguassu LA, de Aquino Assis JG, de Paula Carvalho Silva AB, Vieira Cardoso R de C. Urban gardening and neglected and underutilized species in Salvador, Bahia, Brazil. JOURNAL OF ETHNOBIOLOGY AND ETHNOMEDICINE. 2020;16. doi:[10.1186/s13002-020-00421-0](https://doi.org/10.1186/s13002-020-00421-0)

220.

Weber H, Wiek A, Lang DJ. Sustainability entrepreneurship to address large distances in international food supply. BUSINESS STRATEGY AND DEVELOPMENT. 2020;3: 318–331. doi:[10.1002/bsd2.97](https://doi.org/10.1002/bsd2.97)

221.

Panday US, Pratihast AK, Aryal J, Kayastha RB. A Review on Drone-Based Data Solutions for Cereal Crops. DRONES. 2020;4. doi:[10.3390/drones4030041](https://doi.org/10.3390/drones4030041)

222.

Bounie D, Arcot J, Cole M, Egal F, Juliano P, Mejia C, et al. The role of food science and technology in humanitarian response. TRENDS IN FOOD SCIENCE & TECHNOLOGY. 2020;103: 367–375. doi:[10.1016/j.tifs.2020.06.006](https://doi.org/10.1016/j.tifs.2020.06.006)

223.

April-Lalonde G, Latorre S, Paredes M, Hurtado MF, Munoz F, Deaconu A, et al. Characteristics and Motivations of Consumers of Direct Purchasing Channels and the Perceived Barriers to Alternative Food Purchase: A Cross-Sectional Study in the Ecuadorian Andes. SUSTAINABILITY. 2020;12. doi:[10.3390/su12176923](https://doi.org/10.3390/su12176923)

224.

Kusz B, Kilar J. CONSUMERS’ PREFERENCES FOR PLACES TO PURCHASE LOCAL DAIRY PRODUCTS. AGROLIFE SCIENTIFIC JOURNAL. 2020;9: 198–204.

225.

King HB. Whose work is real work? A triple labor framework for sustainable development initiatives. ECONOMIC ANTHROPOLOGY. 2020;7: 215–227. doi:[10.1002/sea2.12174](https://doi.org/10.1002/sea2.12174)

226.

Goszczynski W, Wroblewski M. Beyond rural idyll? Social imaginaries, motivations and relations in Polish alternative food networks. JOURNAL OF RURAL STUDIES. 2020;76: 254–263. doi:[10.1016/j.jrurstud.2020.04.031](https://doi.org/10.1016/j.jrurstud.2020.04.031)

227.

Fogarassy C, Nagy-Percsi K, Ajibade S, Gyuricza C, Ymeri P. Relations between Circular Economic “Principles” and Organic Food Purchasing Behavior in Hungary. AGRONOMY-BASEL. 2020;10. doi:[10.3390/agronomy10050616](https://doi.org/10.3390/agronomy10050616)

228.

Bisht IS, Rana JC, Ahlawat SP. The Future of Smallholder Farming in India: Some Sustainability Considerations. SUSTAINABILITY. 2020;12. doi:[10.3390/su12093751](https://doi.org/10.3390/su12093751)

229.

Rucabado-Palomar T, Cuellar-Padilla M. Short food supply chains for local food: a difficult path. RENEWABLE AGRICULTURE AND FOOD SYSTEMS. 2020;35: 182–191. doi:[10.1017/S174217051800039X](https://doi.org/10.1017/S174217051800039X)

230.

Watson DJ. Working the fields: The organization of labour in community supported agriculture. ORGANIZATION. 2020;27: 291–313. doi:[10.1177/1350508419888898](https://doi.org/10.1177/1350508419888898)

231.

Yu W, Spencer DM. A qualitative study of visitors to small-scale farms on a tropical Island. Tourism Recreation Research. 2020;45: 389–400. doi:[10.1080/02508281.2019.1710678](https://doi.org/10.1080/02508281.2019.1710678)

232.

von Braun J. Climate Change Risks for Agriculture, Health, and Nutrition. Health of People, Health of Planet and Our Responsibility: Climate Change, Air Pollution and Health. 2020; 135–148. doi:[10.1007/978-3-030-31125-4_11](https://doi.org/10.1007/978-3-030-31125-4_11)

233.

Torres AP. Research report: For young americans, sustainable is not organic. Journal of Food Distribution Research. 2020;51: 71–77.

234.

Thompson D, Johnson KR, Cistrunk KM, Vancil-Leap A, Nyatta T, Hossfeld L, et al. Assemblage, food justice, and intersectionality in rural Mississippi: the Oktibbeha Food Policy Council. Sociological Spectrum. 2020;40: 381–399. doi:[10.1080/02732173.2020.1801541](https://doi.org/10.1080/02732173.2020.1801541)

235.

Szegedyné Fricz Á, Ittzés A, Ózsvári L, Szakos D, Kasza G. Consumer perception of local food products in Hungary. British Food Journal. 2020;122: 2965–2979. doi:[10.1108/BFJ-07-2019-0528](https://doi.org/10.1108/BFJ-07-2019-0528)

236.

Slamet AS, Hadiguna RA, Mulyati H. Making food supply chain sustainable: participating smallholder farmers in modern retail channels. INTERNATIONAL JOURNAL OF SUSTAINABLE AGRICULTURAL MANAGEMENT AND INFORMATICS. 2020;6: 135–162. doi:[10.1504/IJSAMI.2020.108361](https://doi.org/10.1504/IJSAMI.2020.108361)

237.

Seidel M. Sustainable Consumption graduate course: a project-based approach to learning. Food, Culture and Society. 2020;23: 86–92. doi:[10.1080/15528014.2019.1679565](https://doi.org/10.1080/15528014.2019.1679565)

238.

Seguin RA, McGuirt JT, Jilcott Pitts SB, Garner J, Hanson KL, Kolodinsky J, et al. Knowledge and Experience Related to Community Supported Agriculture and Local Foods among Nutrition Educators. Journal of Hunger and Environmental Nutrition. 2020;15: 251–263. doi:[10.1080/19320248.2018.1549520](https://doi.org/10.1080/19320248.2018.1549520)

239.

Ramos LA. Entangled alternatives: political-economic conditions constructing farmer training programs as solutions to the farming crisis. Journal of Political Ecology. 2020;27: 1148–1165. doi:[10.2458/V27I1.23241](https://doi.org/10.2458/V27I1.23241)

240.

Qingbin WANG, LIU C-Q, ZHAO Y-F, KITSOS A, CANNELLA M, WANG S-K, et al. Impacts of the COVID-19 pandemic on the dairy industry: Lessons from China and the United States and policy implications. Journal of Integrative Agriculture. 2020;19: 2903–2915. doi:[10.1016/S2095-3119(20)63443-8](https://doi.org/10.1016/S2095-3119(20)63443-8)

241.

Pretty J, Attwood S, Bawden R, van den Berg H, Bharucha ZP, Dixon J, et al. Assessment of the growth in social groups for sustainable agriculture and land management. GLOBAL SUSTAINABILITY. 2020;3. doi:[10.1017/sus.2020.19](https://doi.org/10.1017/sus.2020.19)

242.

Popławska M. Towards producer-consumer cooperation: Collective learning in alternative food networks as a food sovereignty practice. Praktyka Teoretyczna. 2020;38: 49–73. doi:[10.14746/prt2020.4.3](https://doi.org/10.14746/prt2020.4.3)

243.

Mittal A, Grimm J. COMMENTARY ON COVID-19 AND THE FOOD SYSTEM: ICT solutions to support local food supply chains during the COVID-19 pandemic. Journal of Agriculture, Food Systems, and Community Development. 2020;10: 237–241. doi:[10.5304/jafscd.2020.101.015](https://doi.org/10.5304/jafscd.2020.101.015)

244.

Michel-Villarreal R, Vilalta-Perdomo EL, Hingley M. Exploring producers’ motivations and challenges within a farmers’ market. British Food Journal. 2020;122: 2089–2103. doi:[10.1108/BFJ-09-2019-0731](https://doi.org/10.1108/BFJ-09-2019-0731)

245.

Maslak N, Lei Z, Xu L. ANALYSIS OF AGRICULTURAL TRADE IN CHINA BASED ON THE THEORY OF FACTOR ENDOWMENT. AGRICULTURAL AND RESOURCE ECONOMICS-INTERNATIONAL SCIENTIFIC E-JOURNAL. 2020;6: 50–61.

246.

Lawrence G, Smith K. Neoliberal Globalization and Beyond: Food, Farming, and the Environment. The Cambridge Handbook of Environmental Sociology: Volume 2. 2020;2: 411–428. doi:[10.1017/9781108554558.026](https://doi.org/10.1017/9781108554558.026)

247.

Kurtsal Y, Ayalp EK, Viaggi D. Exploring governance mechanisms, collaborative processes and main challenges in short food supply chains: The case of turkey. Bio-based and Applied Economics. 2020;9: 201–221. doi:[10.13128/bae-8242](https://doi.org/10.13128/bae-8242)

248.

Ku HB, Kan K. Social work and sustainable rural development: The practice of social economy in China. International Journal of Social Welfare. 2020;29: 346–355. doi:[10.1111/ijsw.12422](https://doi.org/10.1111/ijsw.12422)

249.

Kirby CK, Goralnik L, Hodbod J, Piso Z, Libarkin JC. Resilience characteristics of the urban agriculture system in Lansing, Michigan: Importance of support actors in local food systems. Urban Agriculture and Regional Food Systems. 2020;5. doi:[10.1002/uar2.20003](https://doi.org/10.1002/uar2.20003)

250.

Kaiser ML, Ryan-Simkins K, Dionne J, Pence EK. Connecting small-scale producers and consumers: Exploring the feasibility of online food hubs in low-income communities. JOURNAL OF AGRICULTURE FOOD SYSTEMS AND COMMUNITY DEVELOPMENT. 2020;9: 179–196. doi:[10.5304/jafscd.2020.093.019](https://doi.org/10.5304/jafscd.2020.093.019)

251.

Ji C, Chen Q, Zhuo N. Enhancing consumer trust in short food supply chains: The case evidence from three agricultural e-commerce companies in China. Journal of Agribusiness in Developing and Emerging Economies. 2020;10: 103–116. doi:[10.1108/JADEE-12-2018-0180](https://doi.org/10.1108/JADEE-12-2018-0180)

252.

Hedberg RC, Zimmerer KS. What’s the market got to do with it? Social-ecological embeddedness and environmental practices in a local food system initiative. Geoforum. 2020;110: 35–45. doi:[10.1016/j.geoforum.2020.01.022](https://doi.org/10.1016/j.geoforum.2020.01.022)

253.

Hedberg RC. Coming Out of the Foodshed: Phosphorus Cycles and the Many Scales of Local Food. Annals of the American Association of Geographers. 2020;110: 684–704. doi:[10.1080/24694452.2019.1630248](https://doi.org/10.1080/24694452.2019.1630248)

254.

Greco L, Kolodinsky J, Sitaker M, Chase L, Conner D, Estrin H, et al. Farm Fresh Food Boxes: A pilot that examined relationships in value chain partnerships. JOURNAL OF AGRICULTURE FOOD SYSTEMS AND COMMUNITY DEVELOPMENT. 2020;9: 113–129. doi:[10.5304/jafscd.2020.094.012](https://doi.org/10.5304/jafscd.2020.094.012)

255.

Goszczyński W. In Search of the Vocabulary for Eastern European Food Studies. Conceptual Remarks after the Workshop: Alternative Food Supply Networks in Central and Eastern Europe. Eastern European Countryside. 2020;25: 273–279. doi:[10.12775/eec.2019.014](https://doi.org/10.12775/eec.2019.014)

256.

Frazzoli C. Field anthropological research for context-effective risk analysis science in traditional cultures: the case of Senegal. Journal of Global Health Reports. 2020;4. doi:[10.29392/001c.12922](https://doi.org/10.29392/001c.12922)

257.

de Molina MG. Strategies for scaling up agroecological experiences in the European Union. International Journal of Agriculture and Natural Resources. 2020;47: 187–203. doi:[10.7764/ijanr.v47i3.2257](https://doi.org/10.7764/ijanr.v47i3.2257)

258.

Darby KJ, Hinton T, Torre J. The motivations and needs of rural, low-income household food gardeners. Journal of Agriculture, Food Systems, and Community Development. 2020;9: 55–69. doi:[10.5304/jafscd.2020.092.002](https://doi.org/10.5304/jafscd.2020.092.002)

259.

Collins LA. The effect of farmers’ market access on residential property values. Applied Geography. 2020;123. doi:[10.1016/j.apgeog.2020.102272](https://doi.org/10.1016/j.apgeog.2020.102272)

260.

Chapman AM, Perkins HA. Malign and benign neglect: a local food system and the myth of sustainable redevelopment in Appalachia Ohio. Agriculture and Human Values. 2020;37: 113–127. doi:[10.1007/s10460-019-09976-5](https://doi.org/10.1007/s10460-019-09976-5)

261.

Cassol A, Vargas LP, Canever MD. Territorial development, covid-19 and the new strategies of production, commercialization and food consumption of family farming in the southern region of rio grande do sul. Revista Brasileira de Gestao e Desenvolvimento Regional. 2020;16: 374–387.

262.

Cameron G, Rosado FRP, Mederos DDD. Agricultural co-operatives in Canada and Cuba: trends, prospects and ways forward. Environment, Development and Sustainability. 2020;22: 643–660. doi:[10.1007/s10668-018-0213-0](https://doi.org/10.1007/s10668-018-0213-0)

263.

Blumberg R, Leitner H, Cadieux KV. For food space: Theorizing alternative food networks beyond alterity. Journal of Political Ecology. 2020;27: 1–22. doi:[10.2458/v27i1.23026](https://doi.org/10.2458/v27i1.23026)

264.

Ballamingie P, Nimmo ER, Blay-Palmer AD, Stahlbrand L, Knezevic I, Ayalon R, et al. Integrating a food systems lens into discussions of urban resilience: Analyzing the policy environment. Journal of Agriculture, Food Systems, and Community Development. 2020;9: 227–243. doi:[10.5304/jafscd.2020.093.021](https://doi.org/10.5304/jafscd.2020.093.021)

265.

Armesto-López XA, Gómez-Martín MB, Cors-Iglesias M, Martínez-Ibarra E. Short food supply chains in barcelona’s markets. WIT Transactions on Ecology and the Environment. 2020;243: 15–24. doi:[10.2495/UA200021](https://doi.org/10.2495/UA200021)

266.

Harrison B, Foley C, Edwards D, Donaghy G. Outcomes and challenges of an international convention centre’s local procurement strategy. TOURISM MANAGEMENT. 2019;75: 328–339. doi:[10.1016/j.tourman.2019.05.004](https://doi.org/10.1016/j.tourman.2019.05.004)

267.

Sureau S, Lohest F, Van Mol J, Bauler T, Achten WMJ. How Do Chain Governance and Fair Trade Matter? A S-LCA Methodological Proposal Applied to Food Products from Belgian Alternative Chains (Part 2). RESOURCES-BASEL. 2019;8. doi:[10.3390/resources8030145](https://doi.org/10.3390/resources8030145)

268.

Bruce AB. Farm entry and persistence: Three pathways into alternative agriculture in southern Ohio. JOURNAL OF RURAL STUDIES. 2019;69: 30–40. doi:[10.1016/j.jrurstud.2019.04.007](https://doi.org/10.1016/j.jrurstud.2019.04.007)

269.

Samoggia A, Perazzolo C, Kocsis P, Del Prete M. Community Supported Agriculture Farmers’ Perceptions of Management Benefits and Drawbacks. SUSTAINABILITY. 2019;11. doi:[10.3390/su11123262](https://doi.org/10.3390/su11123262)

270.

Mars MM, Schau HJ. The Jazziness of Local Food Practice Work: Organization-Level Ingenuity and the Entrepreneurial Formation and Evolution of Local Food Systems. RURAL SOCIOLOGY. 2019;84: 257–283. doi:[10.1111/ruso.12244](https://doi.org/10.1111/ruso.12244)

271.

Liu P, Ravenscroft N, Ding D, Li D. From pioneering to organised business: the development of ecological farming in China. LOCAL ENVIRONMENT. 2019;24: 539–553. doi:[10.1080/13549839.2019.1597032](https://doi.org/10.1080/13549839.2019.1597032)

272.

Givens G, Dunning R. Distributor intermediation in the farm to food service value chain. RENEWABLE AGRICULTURE AND FOOD SYSTEMS. 2019;34: 268–270. doi:[10.1017/S1742170517000746](https://doi.org/10.1017/S1742170517000746)

273.

Chiffoleau Y, Millet-Amrani S, Rossi A, Guadalupe Rivera-Ferre M, Merino PL. The participatory construction of new economic models in short food supply chains. JOURNAL OF RURAL STUDIES. 2019;68: 182–190. doi:[10.1016/j.jrurstud.2019.01.019](https://doi.org/10.1016/j.jrurstud.2019.01.019)

274.

Figueroa-Rodriguez KA, del Carmen Alvarez-Avila M, Hernandez Castillo F, Schwentesius Rindermann R, Figueroa-Sandoval B. Farmers’ Market Actors, Dynamics, and Attributes: A Bibliometric Study. SUSTAINABILITY. 2019;11. doi:[10.3390/su11030745](https://doi.org/10.3390/su11030745)

275.

Brulard N, Cung V-D, Catusse N, Dutrieux C. An integrated sizing and planning problem in designing diverse vegetable farming systems. INTERNATIONAL JOURNAL OF PRODUCTION RESEARCH. 2019;57: 1018–1036. doi:[10.1080/00207543.2018.1498985](https://doi.org/10.1080/00207543.2018.1498985)

276.

Kulick R. More time in the kitchen, less time on the streets: the micropolitics of cultivating an ethic of care in alternative food networks. LOCAL ENVIRONMENT. 2019;24: 37–51. doi:[10.1080/13549839.2018.1546281](https://doi.org/10.1080/13549839.2018.1546281)

277.

Trivette SA. The importance of food retailers: applying network analysis techniques to the study of local food systems. Agriculture and Human Values. 2019;36: 77–90. doi:[10.1007/s10460-018-9885-1](https://doi.org/10.1007/s10460-018-9885-1)

278.

Suttles S. The time for macroeconomics in municipal food policy. Journal of Agriculture, Food Systems, and Community Development. 2019;8: 29–32. doi:[10.5304/jafscd.2019.084.023](https://doi.org/10.5304/jafscd.2019.084.023)

279.

Soini K, Pouta E, Latvala T, Lilja T. Agrobiodiversity products in alternative food system: Case of finnish native cattle breeds. Sustainability (Switzerland). 2019;11. doi:[10.3390/SU11123408](https://doi.org/10.3390/SU11123408)

280.

Silva EM, Hendrickson J, Mitchell PD, Bietila E. From the field: A participatory approach to assess labor inputs on organic diversified vegetable farms in the Upper Midwestern USA. Renewable Agriculture and Food Systems. 2019;34: 1–6. doi:[10.1017/S1742170517000266](https://doi.org/10.1017/S1742170517000266)

281.

Richardson-Ngwenya P, Whatmore SJ. Food Networks. International Encyclopedia of Human Geography, Second Edition. 2019; 167–173. doi:[10.1016/B978-0-08-102295-5.10183-0](https://doi.org/10.1016/B978-0-08-102295-5.10183-0)

282.

McCune N, Perfecto I, Avilés-Vázquez K, Vázquez-Negrón J, Vandermeer J. Peasant balances and agroecological scaling in Puerto Rican coffee farming. Agroecology and Sustainable Food Systems. 2019;43: 810–826. doi:[10.1080/21683565.2019.1608348](https://doi.org/10.1080/21683565.2019.1608348)

283.

Machida D, Yoshida T. Factors that affect nonmarket fruit and vegetable receptions: Analyses of two cross-sectional surveys in Gunma, Japan. Agriculture (Switzerland). 2019;9. doi:[10.3390/agriculture9110230](https://doi.org/10.3390/agriculture9110230)

284.

Kraus S. San Jose Food Works Study: Demonstrating the Economics of Local Food Systems Toolkit methodology. Journal of Agriculture, Food Systems, and Community Development. 2019;8: 119–135. doi:[10.5304/jafscd.2019.08C.007](https://doi.org/10.5304/jafscd.2019.08C.007)

285.

Khan SS, Arita S, Howitt R, Leung P. A calibrated model of local food system of Hawaii: Whatare the economic implications of the state’s food goals and policies? Natural Resource Modeling. 2019;32. doi:[10.1111/nrm.12196](https://doi.org/10.1111/nrm.12196)

286.

Kacz K, Hegyi J, Gombkoto N. CHARACTERISTICS OF COMMUNITY SUPPORTED AGRICULTURE IN THE WESTERN TRANSDANUBIA REGION. DETUROPE-THE CENTRAL EUROPEAN JOURNAL OF REGIONAL DEVELOPMENT AND TOURISM. 2019;11: 42–54.

287.

Hyland J, Crehan P, Colantuono F, Macken-Walsh A. The Significance of Short Food Supply Chains: Trends and Bottlenecks from the SKIN Thematic Network. STUDIES IN AGRICULTURAL ECONOMICS. 2019;121: 59–66. doi:[10.7896/j.1904](https://doi.org/10.7896/j.1904)

288.

Gromasheva O. Buying directly from farmers: Tactics of St. Petersburg consumers. Demokratizatsiya. 2019;27: 101–128.

289.

Goldenberg MP, Meter K. Building economic multipliers, rather than measuring them: Community-minded ways to develop economic impacts. Journal of Agriculture, Food Systems, and Community Development. 2019;8: 153–164. doi:[10.5304/jafscd.2019.08C.010](https://doi.org/10.5304/jafscd.2019.08C.010)

290.

Francis CA, Lieblein G. Review and critique of the TATA-BOX model. Agroecological Transitions: From Theory to Practice in Local Participatory Design. 2019; 305–321. doi:[10.1007/978-3-030-01953-2](https://doi.org/10.1007/978-3-030-01953-2)

291.

Fernandez-Wulff P. Collective agency in the making: How social innovations in the food system practice democracy beyond consumption. Politics and Governance. 2019;7: 81–93. doi:[10.17645/pag.v7i4.2111](https://doi.org/10.17645/pag.v7i4.2111)

292.

Drejerska N, Bareja-Wawryszuk O, Gołębiewski J. Marginal, localized and restricted activity: Business models for creation a value of local food products: a case from Poland. British Food Journal. 2019;121: 1368–1381. doi:[10.1108/BFJ-05-2018-0337](https://doi.org/10.1108/BFJ-05-2018-0337)

293.

Dong H, Campbell B, Rabinowitz AN. Factors impacting producer marketing through community supported agriculture. PLoS ONE. 2019;14. doi:[10.1371/journal.pone.0219498](https://doi.org/10.1371/journal.pone.0219498)

294.

Dancer A, Newton P, House V. The Difficulties of Developing Local Food Systems: Perspectives of Farmers and Other Key Stakeholders in Boulder County, Colorado. Food Studies. 2019;9: 1–20. doi:[10.18848/2160-1933/CGP/v09i04/1-20](https://doi.org/10.18848/2160-1933/CGP/v09i04/1-20)

295.

Cumming G, Kelmenson S, Norwood C. Local motivations, regional implications: Scaling from local to regional food systems in northeastern North Carolina. Journal of Agriculture, Food Systems, and Community Development. 2019;9: 197–213. doi:[10.5304/jafscd.2019.091.041](https://doi.org/10.5304/jafscd.2019.091.041)

296.

Collison M, Collison T, Myroniuk I, Boyko N, Pellegrini G. Transformation trends in food logistics for short food supply chains - what is new? Studies in Agricultural Economics. 2019;121: 102–110. doi:[10.7896/j.1909](https://doi.org/10.7896/j.1909)

297.

Christensen L, Limbach L. Finding common ground: Defining agricultural viability and streamlining multi-organization data collection. Journal of Agriculture, Food Systems, and Community Development. 2019;8: 137–152. doi:[10.5304/jafscd.2019.08C.005](https://doi.org/10.5304/jafscd.2019.08C.005)

298.

Castaño LSÁ, Castro MAC, Vergara SDQ, Bedoya XM, Paniagua LMR. Organic food consumption: Is it possible to develop public policy? a case study of medellín. Nutricion Hospitalaria. 2019;36: 640–646. doi:[10.20960/nh.2022](https://doi.org/10.20960/nh.2022)

299.

Çalik M. European union short food supply chain policy and environmental management accounting. Handbook of Research on Social and Economic Development in the European Union. 2019; 276–287. doi:[10.4018/978-1-7998-1188-6.ch017](https://doi.org/10.4018/978-1-7998-1188-6.ch017)

300.

Blumberg R, Mincyte D. Infrastructures of taste: Rethinking local food histories in Lithuania. Appetite. 2019;138: 252–259. doi:[10.1016/j.appet.2019.02.016](https://doi.org/10.1016/j.appet.2019.02.016)

301.

Barbieri C, Stevenson KT, Knollenberg W. Broadening the utilitarian epistemology of agritourism research through children and families. Current Issues in Tourism. 2019;22: 2333–2336. doi:[10.1080/13683500.2018.1497011](https://doi.org/10.1080/13683500.2018.1497011)

302.

Astier M, Odenthal G, Patricio C, Orozco-Ramírez Q. Handmade tortilla production in the basins of lakes Pátzcuaro and Zirahuén, Mexico. Journal of Maps. 2019;15: 52–57. doi:[10.1080/17445647.2019.1576553](https://doi.org/10.1080/17445647.2019.1576553)

303.

Thompson HA, Mason CW, Robidoux MA. Hoop House Gardening in the Wapekeka First Nation as an Extension of Land-Based Food Practices. ARCTIC. 2018;71: 407–421. doi:[10.14430/arctic4746](https://doi.org/10.14430/arctic4746)

304.

Diaz II, Hunsberger C. Can agroecological coffee be part of a food sovereignty strategy in Puerto Rico? GEOFORUM. 2018;97: 84–94. doi:[10.1016/j.geoforum.2018.10.016](https://doi.org/10.1016/j.geoforum.2018.10.016)

305.

Forssell S, Lankoski L. Shaping norms. A convention theoretical examination of alternative food retailers as food sustainability transition actors. JOURNAL OF RURAL STUDIES. 2018;63: 46–56. doi:[10.1016/j.jrurstud.2018.04.015](https://doi.org/10.1016/j.jrurstud.2018.04.015)

306.

Saulters MM, Hendrickson MK, Chaddad F. Fairness in alternative food networks: an exploration with midwestern social entrepreneurs. AGRICULTURE AND HUMAN VALUES. 2018;35: 611–621. doi:[10.1007/s10460-018-9852-x](https://doi.org/10.1007/s10460-018-9852-x)

307.

Ngutu M, Bukachi S, Olungah CO, Kiteme B, Kaeser F, Haller T. The Actors, Rules and Regulations Linked to Export Horticulture Production and Access to Land and Water as Common Pool Resources in Laikipia County, Northwest Mount Kenya. LAND. 2018;7. doi:[10.3390/land7030110](https://doi.org/10.3390/land7030110)

308.

Donkor E, Owusu V, Owusu-Sekyere E, Ogundeji AA. The Adoption of Farm Innovations among Rice Producers in Northern Ghana: Implications for Sustainable Rice Supply. AGRICULTURE-BASEL. 2018;8. doi:[10.3390/agriculture8080121](https://doi.org/10.3390/agriculture8080121)

309.

Glennie C, Alkon AH. Food justice: cultivating the field. ENVIRONMENTAL RESEARCH LETTERS. 2018;13. doi:[10.1088/1748-9326/aac4b2](https://doi.org/10.1088/1748-9326/aac4b2)

310.

Beacham J. Organising food differently: Towards a more-than-human ethics of care for the Anthropocene. ORGANIZATION. 2018;25: 533–549. doi:[10.1177/1350508418777893](https://doi.org/10.1177/1350508418777893)

311.

Parodi G. Agroecological transition and reconfiguration of horticultural work among family farmers in Buenos Aires, Argentina. CAHIERS AGRICULTURES. 2018;27. doi:[10.1051/cagri/2018020](https://doi.org/10.1051/cagri/2018020)

312.

Wezel A, Goette J, Lagneaux E, Passuello G, Reisman E, Rodier C, et al. Agroecology in Europe: Research, Education, Collective Action Networks, and Alternative Food Systems. SUSTAINABILITY. 2018;10. doi:[10.3390/su10041214](https://doi.org/10.3390/su10041214)

313.

de Roest K, Ferrari P, Knickel K. Specialisation and economies of scale or diversification and economies of scope? Assessing different agricultural development pathways. JOURNAL OF RURAL STUDIES. 2018;59: 222–231. doi:[10.1016/j.jrurstud.2017.04.013](https://doi.org/10.1016/j.jrurstud.2017.04.013)

314.

Al Shamsi KB, Compagnoni A, Timpanaro G, Cosentino SL, Guarnaccia P. A Sustainable Organic Production Model for “Food Sovereignty” in the United Arab Emirates and Sicily-Italy. SUSTAINABILITY. 2018;10. doi:[10.3390/su10030620](https://doi.org/10.3390/su10030620)

315.

Tewari M, Kelmenson S, Guinn A, Cumming G, Colloredo-Mansfeld R. Mission-Driven Intermediaries as Anchors of the Middle Ground in the American Food System: Evidence from Warrenton, NC. Culture, Agriculture, Food and Environment. 2018;40: 114–123. doi:[10.1111/cuag.12175](https://doi.org/10.1111/cuag.12175)

316.

Tang J-W, Chen M-L, Chiu T-H. An exploratory study on local brand value development for Outlying Island Agriculture: Local food system and actor-network theory perspectives. Sustainability (Switzerland). 2018;10. doi:[10.3390/su10114186](https://doi.org/10.3390/su10114186)

317.

Skog KL, Eriksen SE, Brekken CA, Francis C. Building resilience in social-ecological food systems in Vermont. Sustainability (Switzerland). 2018;10. doi:[10.3390/su10124813](https://doi.org/10.3390/su10124813)

318.

Schmutz U, Kneafsey M, Kay CS, Doernberg A, Zasada I. Sustainability impact assessments of different urban short food supply chains: Examples from London, UK. Renewable Agriculture and Food Systems. 2018;33: 518–529. doi:[10.1017/S1742170517000564](https://doi.org/10.1017/S1742170517000564)

319.

Sandler R. An Ethical Theory Analysis of the Food System Discourse. International Library of Environmental, Agricultural and Food Ethics. 2018;27: 133–143. doi:[10.1007/978-3-319-92603-2_8](https://doi.org/10.1007/978-3-319-92603-2_8)

320.

Reckinger R. Social change for sustainable localised food sovereignty: Convergence between prosumers and ethical entrepreneurs*. Sociologia del Lavoro. 2018; 174–192. doi:[10.3280/SL2018-152010](https://doi.org/10.3280/SL2018-152010)

321.

Pérez-Neira D, Grollmus-Venegas A. Life-cycle energy assessment and carbon footprint of peri-urban horticulture. A comparative case study of local food systems in Spain. Landscape and Urban Planning. 2018;172: 60–68. doi:[10.1016/j.landurbplan.2018.01.001](https://doi.org/10.1016/j.landurbplan.2018.01.001)

322.

Nasiyev B, Tlepov A, Zhanatalapov N, Bekkaliev A, Yeleshev R. Studying agrophytocenoses of Sudan grass in the dry steppe zone of West Kazakhstan. Asian Journal of Microbiology, Biotechnology and Environmental Sciences. 2018;20: 594–600.

323.

Larimore S. Cultural Boundaries to Access in Farmers Markets Accepting Supplemental Nutrition Assistance Program (SNAP). Qualitative Sociology. 2018;41: 63–87. doi:[10.1007/s11133-017-9370-y](https://doi.org/10.1007/s11133-017-9370-y)

324.

Horst M, Gwin L. Land access for direct market food farmers in Oregon, USA. Land Use Policy. 2018;75: 594–611. doi:[10.1016/j.landusepol.2018.01.018](https://doi.org/10.1016/j.landusepol.2018.01.018)

325.

Hashem S, Migliore G, Schifani G, Schimmenti E, Padel S. Motives for buying local, organic food through English box schemes. British Food Journal. 2018;120: 1600–1614. doi:[10.1108/BFJ-08-2017-0426](https://doi.org/10.1108/BFJ-08-2017-0426)

326.

Hamann S. Agro-industrialisation and food security: dietary diversity and food access of workers in Cameroon’s palm oil sector. Canadian Journal of Development Studies. 2018;39: 72–88. doi:[10.1080/02255189.2017.1336079](https://doi.org/10.1080/02255189.2017.1336079)

327.

Gunarathne ADN, Navaratne DG, Pakianathan AE, Perera N YT. Sustainable food supply chain management: An integrated framework and practical perspectives. Understanding Complex Systems. 2018; 289–315. doi:[10.1007/978-3-319-94322-0_11](https://doi.org/10.1007/978-3-319-94322-0_11)

328.

Giomi T, Runhaar P, Runhaar H. Reducing agrochemical use for nature conservation by Italian olive farmers: an evaluation of public and private governance strategies. International Journal of Agricultural Sustainability. 2018;16: 94–105. doi:[10.1080/14735903.2018.1424066](https://doi.org/10.1080/14735903.2018.1424066)

329.

Garner B, Ayala C. Consumer supply-chain demands and challenges at farmers’ markets. BRITISH FOOD JOURNAL. 2018;120: 2734–2747. doi:[10.1108/BFJ-03-2018-0154](https://doi.org/10.1108/BFJ-03-2018-0154)

330.

Fraňková E, Cattaneo C. Organic farming in the past and today: sociometabolic perspective on a Central European case study. Regional Environmental Change. 2018;18: 951–963. doi:[10.1007/s10113-016-1099-8](https://doi.org/10.1007/s10113-016-1099-8)

331.

Flores H, Villalobos JR. A modeling framework for the strategic design of local fresh-food systems. Agricultural Systems. 2018;161: 1–15. doi:[10.1016/j.agsy.2017.12.001](https://doi.org/10.1016/j.agsy.2017.12.001)

332.

Fang X, Huang H, Leung P. Competitiveness of local food: an empirical analysis of the tomato market dynamics. INTERNATIONAL FOOD AND AGRIBUSINESS MANAGEMENT REVIEW. 2018;21: 89–100. doi:[10.22434/IFAMR2016.0139](https://doi.org/10.22434/IFAMR2016.0139)

333.

Enjolras G, Aubert M. Short food supply chains and the issue of sustainability: a case study of French fruit producers. INTERNATIONAL JOURNAL OF RETAIL & DISTRIBUTION MANAGEMENT. 2018;46: 194–209. doi:[10.1108/IJRDM-08-2016-0132](https://doi.org/10.1108/IJRDM-08-2016-0132)

334.

Dumas SE, Lewis D, Travis AJ. Small-scale egg production centres increase children’s egg consumption in rural Zambia. Maternal and Child Nutrition. 2018;14. doi:[10.1111/mcn.12662](https://doi.org/10.1111/mcn.12662)

335.

Ding D, Liu P, Ravenscroft N. The new urban agricultural geography of Shanghai. Geoforum. 2018;90: 74–83. doi:[10.1016/j.geoforum.2018.02.010](https://doi.org/10.1016/j.geoforum.2018.02.010)

336.

Deppermann A, Havlík P, Valin H, Boere E, Herrero M, Vervoort J, et al. The market impacts of shortening feed supply chains in Europe. Food Security. 2018;10: 1401–1410. doi:[10.1007/s12571-018-0868-2](https://doi.org/10.1007/s12571-018-0868-2)

337.

Charatsari C, Kitsios F, Stafyla A, Aidonis D, Lioutas E. Antecedents of farmers’ willingness to participate in short food supply chains. British Food Journal. 2018;120: 2317–2333. doi:[10.1108/BFJ-09-2017-0537](https://doi.org/10.1108/BFJ-09-2017-0537)

338.

Brinkley C. The smallworld of the alternative food network. Sustainability (Switzerland). 2018;10. doi:[10.3390/su10082921](https://doi.org/10.3390/su10082921)

339.

Balázs B, Pataki G. Cooperative research for bottom-up food sovereignty and policy change. Action Research in Policy Analysis: Critical and Relational Approaches to Sustainability Transitions. 2018; 39–60. doi:[10.4324/9781315148724-4](https://doi.org/10.4324/9781315148724-4)

340.

Aguiar L da C, DelGrossi ME, Thome KM. Short food supply chain: characteristics of a family farm. CIENCIA RURAL. 2018;48. doi:[10.1590/0103-8478cr20170775](https://doi.org/10.1590/0103-8478cr20170775)

341.

McInnes A, Fraser E, Gedalof Z, Silver J. A quantitative analysis of food movement convergence in four Canadian provinces. AGRICULTURE AND HUMAN VALUES. 2017;34: 787–804. doi:[10.1007/s10460-017-9775-y](https://doi.org/10.1007/s10460-017-9775-y)

342.

Bruce AB, Castellano RLS. Labor and alternative food networks: challenges for farmers and consumers. RENEWABLE AGRICULTURE AND FOOD SYSTEMS. 2017;32: 403–416. doi:[10.1017/S174217051600034X](https://doi.org/10.1017/S174217051600034X)

343.

Dupre L, Lamine C, Navarrete M. Short Food Supply Chains, Long Working Days: Active Work and the Construction of Professional Satisfaction in French Diversified Organic Market Gardening. SOCIOLOGIA RURALIS. 2017;57: 396–414. doi:[10.1111/soru.12178](https://doi.org/10.1111/soru.12178)

344.

Galt RE, Bradley K, Christensen L, Fake C, Munden-Dixon K, Simpson N, et al. What difference does income make for Community Supported Agriculture (CSA) members in California? Comparing lower-income and higher-income households. AGRICULTURE AND HUMAN VALUES. 2017;34: 435–452. doi:[10.1007/s10460-016-9724-1](https://doi.org/10.1007/s10460-016-9724-1)

345.

Aggestam V, Fleiss E, Posch A. Scaling-up short food supply chains? A survey study on the drivers behind the intention of food producers. JOURNAL OF RURAL STUDIES. 2017;51: 64–72. doi:[10.1016/j.jrurstud.2017.02.003](https://doi.org/10.1016/j.jrurstud.2017.02.003)

346.

Zhou Z, Shen Y, Du C, Zhou J, Qin Y, Wu Y. Economic and Soil Environmental Benefits of Using Controlled-Release Bulk Blending Urea in the North China Plain. Land Degradation and Development. 2017;28: 2370–2379. doi:[10.1002/ldr.2767](https://doi.org/10.1002/ldr.2767)

347.

VanWinkle TN. “Savor the earth to save it!”—The pedagogy of sustainable pleasure and relational ecology in a place-based public culinary culture. Food and Foodways. 2017;25: 40–57. doi:[10.1080/07409710.2017.1270648](https://doi.org/10.1080/07409710.2017.1270648)

348.

Van Sant L. When Local Comes to Town: Governing Local Agriculture in the South Carolina Lowcountry. Capitalism, Nature, Socialism. 2017;28: 64–83. doi:[10.1080/10455752.2016.1246583](https://doi.org/10.1080/10455752.2016.1246583)

349.

Turkkan C. What Is Being Sustained? Sustainability and Food Exchange Sites in Istanbul. Gender, Development and Social Change. 2017; 119–153. doi:[10.1057/978-1-349-95182-6_6](https://doi.org/10.1057/978-1-349-95182-6_6)

350.

Tulla AF, Vera A, Valldeperas N, Guirado C. New approaches to sustainable rural development: Social farming as an opportunity in Europe? Human Geographies. 2017;11: 25–40. doi:[10.5719/hgeo.2017.111.2](https://doi.org/10.5719/hgeo.2017.111.2)

351.

Som Castellano RL. Receiving assistance and local food system participation. Social Sciences. 2017;6. doi:[10.3390/socsci6010018](https://doi.org/10.3390/socsci6010018)

352.

Padro R, Marco I, Cattaneo C, Caravaca J, Tello E. Does Your Landscape Mirror What You Eat? A Long-Term Socio-metabolic Analysis of a Local Food System in Valles County (Spain, 1860-1956-1999) - SOCIO-METABOLIC PERSPECTIVES ON THE SUSTAINABILITY OF LOCAL FOOD SYSTEMS: INSIGHTS FOR SCIENCE, POLICY AND PRACTICE. Frankova E, Haas W, Singh S, editors. SOCIO-METABOLIC PERSPECTIVES ON THE SUSTAINABILITY OF LOCAL FOOD SYSTEMS: INSIGHTS FOR SCIENCE, POLICY AND PRACTICE. 2017;7: 133–164. doi:[10.1007/978-3-319-69236-4_5](https://doi.org/10.1007/978-3-319-69236-4_5)

353.

Nu J, Bersamin A. Collaborating with Alaska Native communities to design a cultural food intervention to address nutrition transition. Progress in Community Health Partnerships: Research, Education, and Action. 2017;11: 71–80. doi:[10.1353/cpr.2017.0009](https://doi.org/10.1353/cpr.2017.0009)

354.

Nelson P, Beckie MA, Krogman NT. The “Locavore” Chef in Alberta: A Situated Social Practice Analysis. FOOD CULTURE & SOCIETY. 2017;20: 503–524. doi:[10.1080/15528014.2017.1288798](https://doi.org/10.1080/15528014.2017.1288798)

355.

McLaughlin J. Strengthening the backbone: Local food, foreign labour and social justice. Nourishing Communities: From Fractured Food Systems to Transformative Pathways. 2017; 23–40. doi:[10.1007/978-3-319-57000-6_2](https://doi.org/10.1007/978-3-319-57000-6_2)

356.

Martin S, Horst M. Recipe for resiliency: There’s more to community food systems than farmers markets. Planning. 2017;83: 34–38.

357.

Mars MM, Schau HJ. Institutional entrepreneurship and the negotiation and blending of multiple logics in the Southern Arizona local food system. Agriculture and Human Values. 2017;34: 407–422. doi:[10.1007/s10460-016-9722-3](https://doi.org/10.1007/s10460-016-9722-3)

358.

Machado MR. Alternative to What? Agroecology, Food Sovereignty, and Cuba’s Agricultural Revolution. Human Geography(United Kingdom). 2017;10: 7–21. doi:[10.1177/194277861701000302](https://doi.org/10.1177/194277861701000302)

359.

Lutz J, Smetschka B, Grima N. Farmer cooperation as a means for creating local food systems-Potentials and challenges. Sustainability (Switzerland). 2017;9. doi:[10.3390/su9060925](https://doi.org/10.3390/su9060925)

360.

Leiper C, Clarke-Sather A. Co-creating an alternative: the moral economy of participating in farmers’ markets. LOCAL ENVIRONMENT. 2017;22: 840–858. doi:[10.1080/13549839.2017.1296822](https://doi.org/10.1080/13549839.2017.1296822)

361.

Laforge JML, Anderson CR, McLachlan SM. Governments, grassroots, and the struggle for local food systems: containing, coopting, contesting and collaborating. Agriculture and Human Values. 2017;34: 663–681. doi:[10.1007/s10460-016-9765-5](https://doi.org/10.1007/s10460-016-9765-5)

362.

Grando S, Carey J, Hegger E, Jahrl I, Ortolani L. Short Food Supply Chains in Urban Areas: Who Takes the Lead? Evidence from Three Cities across Europe. Urban Agriculture and Regional Food Systems. 2017;2: 1–11. doi:[10.2134/urbanag2016.05.0002](https://doi.org/10.2134/urbanag2016.05.0002)

363.

Frankova E, Haas W, Singh SJ. Introduction: Key Concepts, Debates and Approaches in Analysing the Sustainability of Agri-Food Systems - SOCIO-METABOLIC PERSPECTIVES ON THE SUSTAINABILITY OF LOCAL FOOD SYSTEMS: INSIGHTS FOR SCIENCE, POLICY AND PRACTICE. Frankova E, Haas W, Singh S, editors. SOCIO-METABOLIC PERSPECTIVES ON THE SUSTAINABILITY OF LOCAL FOOD SYSTEMS: INSIGHTS FOR SCIENCE, POLICY AND PRACTICE. 2017;7: 1–24. doi:[10.1007/978-3-319-69236-4_1](https://doi.org/10.1007/978-3-319-69236-4_1)

364.

Frankova E, Cattaneo C. Food, Feed, Fuel, Fibre *and* Finance: Looking for Sustainability Halfway Between Traditional Organic and Industrialised Agriculture in the Czech Republic - SOCIO-METABOLIC PERSPECTIVES ON THE SUSTAINABILITY OF LOCAL FOOD SYSTEMS: INSIGHTS FOR SCIENCE, POLICY AND PRACTICE. Frankova E, Haas W, Singh S, editors. SOCIO-METABOLIC PERSPECTIVES ON THE SUSTAINABILITY OF LOCAL FOOD SYSTEMS: INSIGHTS FOR SCIENCE, POLICY AND PRACTICE. 2017;7: 193–229. doi:[10.1007/978-3-319-69236-4_7](https://doi.org/10.1007/978-3-319-69236-4_7)

365.

Francis CA, Jensen ES, Lieblein G, Breland TA. Agroecologist education for sustainable development of farming and food systems. Agronomy Journal. 2017;109: 23–32. doi:[10.2134/agronj2016.05.0267](https://doi.org/10.2134/agronj2016.05.0267)

366.

Ferrazzi G, Ventura V, Ratti S, Balzaretti C. Consumers’ preferences for a local food product: The case of a new Carnaroli rice product in Lombardy. Italian Journal of Food Safety. 2017;6: 71–74. doi:[10.4081/ijfs.2017.6186](https://doi.org/10.4081/ijfs.2017.6186)

367.

Demartini E, Gaviglio A, Pirani A. Farmers’ motivation and perceived effects of participating in short food supply chains: evidence from a North Italian survey. AGRICULTURAL ECONOMICS-ZEMEDELSKA EKONOMIKA. 2017;63: 204–216. doi:[10.17221/323/2015-AGRICECON](https://doi.org/10.17221/323/2015-AGRICECON)

368.

Delgado C. Mapping urban agriculture in Portugal: Lessons from practice and their relevance for European post-crisis contexts. Moravian Geographical Reports. 2017;25: 139–153. doi:[10.1515/mgr-2017-0013](https://doi.org/10.1515/mgr-2017-0013)

369.

Chaparro Africano A, Calle Collado Á. Peasant economy sustainability in peasant markets, Colombia. Agroecology and Sustainable Food Systems. 2017;41: 204–225. doi:[10.1080/21683565.2016.1266069](https://doi.org/10.1080/21683565.2016.1266069)

370.

Bakos IM. Local food systems supported by communities nationally and internationally. DETUROPE. 2017;9: 59–79.

371.

Torquati B, Vigano E, Taglioni C. Construction of Alternative Food Networks for organic products: A case study of “Organized Groups of Supply and Demand.” NEW MEDIT. 2016;15: 53–62.

372.

Recasens X, Alfranca O, Maldonado L. The adaptation of urban farms to cities: The case of the Alella wine region within the Barcelona Metropolitan Region. LAND USE POLICY. 2016;56: 158–168. doi:[10.1016/j.landusepol.2016.04.023](https://doi.org/10.1016/j.landusepol.2016.04.023)

373.

Doernberg A, Zasada I, Bruszewska K, Skoczowski B, Piorr A. Potentials and Limitations of Regional Organic Food Supply: A Qualitative Analysis of Two Food Chain Types in the Berlin Metropolitan Region. SUSTAINABILITY. 2016;8. doi:[10.3390/su8111125](https://doi.org/10.3390/su8111125)

374.

Tizard M, Hallerman E, Fahrenkrug S, Newell-McGloughlin M, Gibson J, de Loos F, et al. Strategies to enable the adoption of animal biotechnology to sustainably improve global food safety and security. TRANSGENIC RESEARCH. 2016;25: 575–595. doi:[10.1007/s11248-016-9965-1](https://doi.org/10.1007/s11248-016-9965-1)

375.

Kristensen DK, Kjeldsen C, Thorsoe MH. Enabling Sustainable Agro-Food Futures: Exploring Fault Lines and Synergies Between the Integrated Territorial Paradigm, Rural Eco-Economy and Circular Economy. JOURNAL OF AGRICULTURAL & ENVIRONMENTAL ETHICS. 2016;29: 749–765. doi:[10.1007/s10806-016-9632-9](https://doi.org/10.1007/s10806-016-9632-9)

376.

Weiler AM, Otero G, Wittman H. Rock Stars and Bad Apples: Moral Economies of Alternative Food Networks and Precarious Farm Work Regimes. ANTIPODE. 2016;48: 1140–1162. doi:[10.1111/anti.12221](https://doi.org/10.1111/anti.12221)

377.

Mincyte D, Dobernig K. Urban farming in the North American metropolis: Rethinking work and distance in alternative food networks. ENVIRONMENT AND PLANNING A-ECONOMY AND SPACE. 2016;48: 1767–1786. doi:[10.1177/0308518X16651444](https://doi.org/10.1177/0308518X16651444)

378.

Clark JK, Inwood SM. Scaling-up regional fruit and vegetable distribution: potential for adaptive change in the food system. AGRICULTURE AND HUMAN VALUES. 2016;33: 503–519. doi:[10.1007/s10460-015-9618-7](https://doi.org/10.1007/s10460-015-9618-7)

379.

Ponte S. Convention theory in the Anglophone agro-food literature: Past, present and future. JOURNAL OF RURAL STUDIES. 2016;44: 12–23. doi:[10.1016/j.jrurstud.2015.12.019](https://doi.org/10.1016/j.jrurstud.2015.12.019)

380.

Phillips C. Alternative food distribution and plastic devices: Performances, valuations, and experimentations. JOURNAL OF RURAL STUDIES. 2016;44: 208–216. doi:[10.1016/j.jrurstud.2016.02.006](https://doi.org/10.1016/j.jrurstud.2016.02.006)

381.

Joosse S. The Making and Re-making of a Regional Product: The Case of Zeeland Madder. SOCIOLOGIA RURALIS. 2016;56: 248–269. doi:[10.1111/soru.12076](https://doi.org/10.1111/soru.12076)

382.

Dixon J, Richards C. On food security and alternative food networks: understanding and performing food security in the context of urban bias. AGRICULTURE AND HUMAN VALUES. 2016;33: 191–202. doi:[10.1007/s10460-015-9630-y](https://doi.org/10.1007/s10460-015-9630-y)

383.

Udoh JP. Sustainable nondestructive mangrove-friendly aquaculture in Nigeria I: Ecological and environmental perspectives. AACL Bioflux. 2016;9: 50–70.

384.

Som Castellano RL. Alternative Food Networks and the Labor of Food Provisioning: A Third Shift? Rural Sociology. 2016;81: 445–469. doi:[10.1111/ruso.12104](https://doi.org/10.1111/ruso.12104)

385.

Schmit TM, Jablonski BBR, Mansury Y. Assessing the Economic Impacts of Local Food System Producers by Scale: A Case Study From New York. Economic Development Quarterly. 2016;30: 316–328. doi:[10.1177/0891242416657156](https://doi.org/10.1177/0891242416657156)

386.

O’Kane G. A moveable feast: Exploring barriers and enablers to food citizenship. Appetite. 2016;105: 674–687. doi:[10.1016/j.appet.2016.07.002](https://doi.org/10.1016/j.appet.2016.07.002)

387.

Niewolny KL, D’Adamo-Damery P. Learning through story as political praxis: The role of narratives in community food work. Learning, Food, and Sustainability: Sites for Resistance and Change. 2016; 113–131. doi:[10.1057/978-1-137-53904-5_7](https://doi.org/10.1057/978-1-137-53904-5_7)

388.

Mundler P, Laughrea S. The contributions of short food supply chains to territorial development: A study of three Quebec territories. Journal of Rural Studies. 2016;45: 218–229. doi:[10.1016/j.jrurstud.2016.04.001](https://doi.org/10.1016/j.jrurstud.2016.04.001)

389.

Meenar MR. Feeding the hungry: Analysis of food insecurity in lower income urban communities. Local Food Systems in Old Industrial Regions: Concepts, Spatial Context, and Local Practices. 2016; 71–91. doi:[10.4324/9781315592855-11](https://doi.org/10.4324/9781315592855-11)

390.

Levitte Y. Thinking About Labour in Alternative Food Systems. Imagining Sustainable Food Systems: Theory and Practice. 2016; 71–86. doi:[10.4324/9781315587905-7](https://doi.org/10.4324/9781315587905-7)

391.

Jonas AEG. ‘Alternative’ this, ‘alternative’ that. ..: Interrogating alterity and diversity. Interrogating Alterity: Alternative Economic and Political Spaces. 2016; 3–27. doi:[10.4324/9781315589633-10](https://doi.org/10.4324/9781315589633-10)

392.

Jablonski BBR, Schmit TM. Differential expenditure patterns of local food system participants. Renewable Agriculture and Food Systems. 2016;31: 139–147. doi:[10.1017/S1742170515000083](https://doi.org/10.1017/S1742170515000083)

393.

i Rico NM, Fuller AM. Newcomers to farming: Towards a new rurality in Europe. Documents d’Analisi Geografica. 2016;62: 531–551. doi:[10.5565/rev/dag.376](https://doi.org/10.5565/rev/dag.376)

394.

Hussain A, Rasul G, Mahapatra B, Tuladhar S. Household food security in the face of climate change in the Hindu-Kush Himalayan region. Food Security. 2016;8: 921–937. doi:[10.1007/s12571-016-0607-5](https://doi.org/10.1007/s12571-016-0607-5)

395.

Ferguson H, Res NRLH. More than something to hold the plants up: soil as a non-human ally in the struggle for food justice. LOCAL ENVIRONMENT. 2016;21: 956–968. doi:[10.1080/13549839.2015.1050659](https://doi.org/10.1080/13549839.2015.1050659)

396.

Desjardins E. The Urban Food Desert: Spatial Inequality or Opportunity for Change? Imagining Sustainable Food Systems: Theory and Practice. 2016; 87–111. doi:[10.4324/9781315587905-8](https://doi.org/10.4324/9781315587905-8)

397.

Conner DS, Garnett BR. Economic and Environmental Drivers of Fruit and Vegetable Intake Among Socioeconomically Diverse Adults in Vermont. Journal of Hunger and Environmental Nutrition. 2016;11: 263–271. doi:[10.1080/19320248.2015.1128862](https://doi.org/10.1080/19320248.2015.1128862)

398.

Betz ME, Farmer JR. Farmers’ market governance and its role on consumer motives and outcomes. Local Environment. 2016;21: 1420–1434. doi:[10.1080/13549839.2015.1129606](https://doi.org/10.1080/13549839.2015.1129606)

399.

Bauermeister MR. Social capital and collective identity in the local food movement. International Journal of Agricultural Sustainability. 2016;14: 123–141. doi:[10.1080/14735903.2015.1042189](https://doi.org/10.1080/14735903.2015.1042189)

400.

Wilkinson J. Food security and the global agrifood system: Ethical issues in historical and sociological perpspective. GLOBAL FOOD SECURITY-AGRICULTURE POLICY ECONOMICS AND ENVIRONMENT. 2015;7: 9–14. doi:[10.1016/j.gfs.2015.12.001](https://doi.org/10.1016/j.gfs.2015.12.001)

401.

Drake L, Lawson LJ. Results of a US and Canada community garden survey: shared challenges in garden management amid diverse geographical and organizational contexts. AGRICULTURE AND HUMAN VALUES. 2015;32: 241–254. doi:[10.1007/s10460-014-9558-7](https://doi.org/10.1007/s10460-014-9558-7)

402.

Turner B, Hope C. Staging the Local: rethinking scale in farmers’ markets. AUSTRALIAN GEOGRAPHER. 2015;46: 147–163. doi:[10.1080/00049182.2015.1020602](https://doi.org/10.1080/00049182.2015.1020602)

403.

Kulak M, Nemecek T, Frossard E, Chable V, Gaillard G. Life cycle assessment of bread from several alternative food networks in Europe. JOURNAL OF CLEANER PRODUCTION. 2015;90: 104–113. doi:[10.1016/j.jclepro.2014.10.060](https://doi.org/10.1016/j.jclepro.2014.10.060)

404.

Leclerc E, d’Arbaumont M, Verron J-P, Goldstein C, Cesar F, Dewonck S. Andra Environmental Specimen Bank: archiving the environmental chemical quality for long-term monitoring. ENVIRONMENTAL SCIENCE AND POLLUTION RESEARCH. 2015;22: 1568–1576. doi:[10.1007/s11356-014-2924-6](https://doi.org/10.1007/s11356-014-2924-6)

405.

Wolff LF, Gomes JCC. Beekeeping and Agroecological Systems for Endogenous Sustainable Development. Agroecology and Sustainable Food Systems. 2015;39: 416–435. doi:[10.1080/21683565.2014.991056](https://doi.org/10.1080/21683565.2014.991056)

406.

Tudisca S, Di Trapani AM, Sgroi F, Testa R. SOCIO-ECONOMIC ASSESSMENT OF DIRECT SALES IN SICILIAN FARMS. ITALIAN JOURNAL OF FOOD SCIENCE. 2015;27.

407.

Tsuchiya K, Hara Y, Thaitakoo D. Linking food and land systems for sustainable peri-urban agriculture in Bangkok Metropolitan Region. Landscape and Urban Planning. 2015;143: 192–204. doi:[10.1016/j.landurbplan.2015.07.008](https://doi.org/10.1016/j.landurbplan.2015.07.008)

408.

Timpanaro G, Lo Giudice V, Foti VT. The interest of consumers to safeguard plant biodiversity: The case of vegetable products. Quality - Access to Success. 2015;16: 237–245.

409.

Som Castellano RL. Alternative food networks and food provisioning as a gendered act. Agriculture and Human Values. 2015;32: 461–474. doi:[10.1007/s10460-014-9562-y](https://doi.org/10.1007/s10460-014-9562-y)

410.

Sadler RC, Arku G, Gilliland JA. Local food networks as catalysts for food policy change to improve health and build the economy. LOCAL ENVIRONMENT. 2015;20: 1103–1121. doi:[10.1080/13549839.2014.894965](https://doi.org/10.1080/13549839.2014.894965)

411.

Ruhf KZ. Regionalism: a New England recipe for a resilient food system. Journal of Environmental Studies and Sciences. 2015;5: 650–660. doi:[10.1007/s13412-015-0324-y](https://doi.org/10.1007/s13412-015-0324-y)

412.

Paltrinieri R, Spillare S. Well-being shift through healthy eating. From organic consumption to a paradigm of alternative local development. Rivista di Studi sulla Sostenibilita. 2015; 83–94. doi:[10.3280/RISS2015-002008](https://doi.org/10.3280/RISS2015-002008)

413.

Miller WM. UK allotments and urban food initiatives: (limited?) potential for reducing inequalities. LOCAL ENVIRONMENT. 2015;20: 1194–1214. doi:[10.1080/13549839.2015.1035239](https://doi.org/10.1080/13549839.2015.1035239)

414.

Migliore G, Schifani G, Romeo P, Hashem S, Cembalo L. Are Farmers in Alternative Food Networks Social Entrepreneurs? Evidence from a Behavioral Approach. Journal of Agricultural and Environmental Ethics. 2015;28: 885–902. doi:[10.1007/s10806-015-9562-y](https://doi.org/10.1007/s10806-015-9562-y)

415.

Mastronardi L, Marino D, Cavallo A, Giannelli A. Exploring the Role of Farmers in Short Food Supply Chains: The Case of Italy. INTERNATIONAL FOOD AND AGRIBUSINESS MANAGEMENT REVIEW. 2015;18: 109–129.

416.

Mastronardi L, Marino D, Cavallo A, Giannelli A. Exploring the role of farmers in short food supply chains: The case of Italy. International Food and Agribusiness Management Review. 2015;18: 109–130.

417.

Holland JH, Thompson OM, Godwin HH, Pavlovich NM, Stewart KB. Farm-to-School Programming in South Carolina: An Economic Impact Projection Analysis. Journal of Hunger and Environmental Nutrition. 2015;10: 526–538. doi:[10.1080/19320248.2014.980045](https://doi.org/10.1080/19320248.2014.980045)

418.

Holben DH, Morrone M. Development of an EcoNutrition Café Series to Increase Awareness and Knowledge of a Sustainable, Local Food System in Appalachian Ohio. Journal of Hunger and Environmental Nutrition. 2015;10: 293–296. doi:[10.1080/19320248.2015.1036603](https://doi.org/10.1080/19320248.2015.1036603)

419.

Clark JK, Sharp JS, Dugan KL. The agrifood system policy agenda and research domain. Journal of Rural Studies. 2015;42: 112–122. doi:[10.1016/j.jrurstud.2015.10.004](https://doi.org/10.1016/j.jrurstud.2015.10.004)

420.

Campbell BC, Veteto JR. Free seeds and food sovereignty: anthropology and grassroots agrobiodiversity conservation strategies in the US South. Journal of Political Ecology. 2015;22: 445–465. doi:[10.2458/v22i1.21118](https://doi.org/10.2458/v22i1.21118)

421.

Buchan R, Cloutier D, Friedman A, Ostry A. Local food system planning: The problem, conceptual issues, and policy tools for local government planners. Canadian Journal of Urban Research. 2015;24: 1–23.

422.

Bertmann FMW, Fricke HE, Carpenter LR, Schober DJ, Smith TM, Pinard CA, et al. A workplace farmstand pilot programme in Omaha, Nebraska, USA. Public Health Nutrition. 2015;18: 2402–2406. doi:[10.1017/S1368980015001706](https://doi.org/10.1017/S1368980015001706)

423.

Derioz P, Loireau M, Bachimon P, Cancel E, Clement D. What place for pastoral activities in the economic transformation of Vicdessos (Ariege Pyrenees)? REVUE DE GEOGRAPHIE ALPINE-JOURNAL OF ALPINE RESEARCH. 2014;102. doi:[10.4000/rga.2398](https://doi.org/10.4000/rga.2398)

424.

Larder N, Lyons K, Woolcock G. Enacting food sovereignty: values and meanings in the act of domestic food production in urban Australia. Local Environment. 2014;19: 56–76. doi:[10.1080/13549839.2012.716409](https://doi.org/10.1080/13549839.2012.716409)

425.

Fabbrizzi S, Menghini S, Marinelli N. The short food supply chain: A concrete example of sustainability. A literature review. Rivista di Studi sulla Sostenibilita. 2014; 189–206. doi:[10.3280/RISS2014-002012](https://doi.org/10.3280/RISS2014-002012)

426.

Erjavec E, Falkowski J, Juvančič L. Structural Change and Agricultural Policy for SSFs: A View from the 2004 NMSs. EuroChoices. 2014;13: 41–45. doi:[10.1111/1746-692X.12051](https://doi.org/10.1111/1746-692X.12051)

427.

Cleveland DA, Müller NM, Tranovich AC, Mazaroli DN, Hinson K. Local food hubs for alternative food systems: A case study from Santa Barbara County, California. Journal of Rural Studies. 2014;35: 26–36. doi:[10.1016/j.jrurstud.2014.03.008](https://doi.org/10.1016/j.jrurstud.2014.03.008)

428.

Aubry C, Kebir L. Shortening food supply chains: A means for maintaining agriculture close to urban areas? The case of the French metropolitan area of Paris. FOOD POLICY. 2013;41: 85–93. doi:[10.1016/j.foodpol.2013.04.006](https://doi.org/10.1016/j.foodpol.2013.04.006)

429.

Wilson AD. Beyond Alternative: Exploring the Potential for Autonomous Food Spaces. ANTIPODE. 2013;45: 719–737. doi:[10.1111/j.1467-8330.2012.01020.x](https://doi.org/10.1111/j.1467-8330.2012.01020.x)

430.

Mulcahy DN, Mulcahy DL, Dietz D. Biochar soil amendment increases tomato seedling resistance to drought in sandy soils. JOURNAL OF ARID ENVIRONMENTS. 2013;88: 222–225. doi:[10.1016/j.jaridenv.2012.07.012](https://doi.org/10.1016/j.jaridenv.2012.07.012)

431.

Patria HD. Uncultivated Biodiversity in Women’s hand: How to create food sovereignty. Asian Journal of Women’s Studies. 2013;19: 148–161. doi:[10.1080/12259276.2013.11666152](https://doi.org/10.1080/12259276.2013.11666152)

432.

Katchova AL, Woods TA. Local Foods and Food Cooperatives: Ethics, Economics and Competition Issues. International Library of Environmental, Agricultural and Food Ethics. 2013;20: 227–242. doi:[10.1007/978-94-007-6274-9_12](https://doi.org/10.1007/978-94-007-6274-9_12)

433.

Hassanein N. Practicing Food Democracy: A Pragmatic Politics of Transformation. Taking Food Public: Redefining Foodways in a Changing World. 2013; 461–474. doi:[10.4324/9781315881065-44](https://doi.org/10.4324/9781315881065-44)

434.

Gerster-Bentaya M. Nutrition-sensitive urban agriculture. Food Security. 2013;5: 723–737. doi:[10.1007/s12571-013-0295-3](https://doi.org/10.1007/s12571-013-0295-3)

435.

Fang M, Buttenheim AM, Havassy J, Gollust SE. “It’s Not an ‘If You Build It They Will Come’ Type of Scenario”: Stakeholder Perspectives on Farmers’ Markets as a Policy Solution to Food Access in Low-Income Neighborhoods. Journal of Hunger and Environmental Nutrition. 2013;8: 39–60. doi:[10.1080/19320248.2012.758065](https://doi.org/10.1080/19320248.2012.758065)

436.

Darnhofer I. Contributing to a transition to sustainability of agri-food systems: Potentials and pitfalls for organic farming. Organic Farming, Prototype for Sustainable Agricultures: Prototype for Sustainable Agricultures. 2013;9789400779273: 439–452. doi:[10.1007/978-94-007-7927-3_24](https://doi.org/10.1007/978-94-007-7927-3_24)

437.

Buckley J, Conner DS, Matts C, Hamm MW. Social Relationships and Farm-to-Institution Initiatives: Complexity and Scale in Local Food Systems. Journal of Hunger and Environmental Nutrition. 2013;8: 397–412. doi:[10.1080/19320248.2013.816988](https://doi.org/10.1080/19320248.2013.816988)

438.

Bailey C. Local solutions to inequality: Steps toward fostering a progressive social movement. Rural Sociology. 2013;78: 411–428. doi:[10.1111/ruso.12032](https://doi.org/10.1111/ruso.12032)

439.

Alkon AH. Food justice: An overview. Routledge International Handbook of Food Studies. 2013; 292–305. doi:[10.4324/9780203819227-36](https://doi.org/10.4324/9780203819227-36)

440.

Lin Y, Vogt R, Larssen T. Environmental mercury in China: A review. ENVIRONMENTAL TOXICOLOGY AND CHEMISTRY. 2012;31: 2431–2444. doi:[10.1002/etc.1980](https://doi.org/10.1002/etc.1980)

441.

Lewis CB, Peters CJ. A capacity assessment of New England’s large animal slaughter facilities as relative to meat production for the regional food system. RENEWABLE AGRICULTURE AND FOOD SYSTEMS. 2012;27: 192–199. doi:[10.1017/S1742170511000305](https://doi.org/10.1017/S1742170511000305)

442.

Hilimire K. The grass is greener: Farmers’ experiences with pastured poultry. RENEWABLE AGRICULTURE AND FOOD SYSTEMS. 2012;27: 173–179. doi:[10.1017/S1742170511000287](https://doi.org/10.1017/S1742170511000287)

443.

Peters CJ, Bills NL, Lembo AJ, Wilkins JL, Fick GW. Mapping potential foodsheds in New York State by food group: An approach for prioritizing which foods to grow locally. RENEWABLE AGRICULTURE AND FOOD SYSTEMS. 2012;27: 125–137. doi:[10.1017/S1742170511000196](https://doi.org/10.1017/S1742170511000196)

444.

Harris D, Lott M, Lakins V, Bowden B, Kimmons J. Farm to Institution: Creating Access to Healthy Local and Regional Foods. ADVANCES IN NUTRITION. 2012;3: 343–349. doi:[10.3945/an.111.001677](https://doi.org/10.3945/an.111.001677)

445.

Hayden J, Buck D. Doing community supported agriculture: Tactile space, affect and effects of membership. GEOFORUM. 2012;43: 332–341. doi:[10.1016/j.geoforum.2011.08.003](https://doi.org/10.1016/j.geoforum.2011.08.003)

446.

Ibnouf FO. Does it really matter whether food is produced and provided by a man or a woman? Food Security: Quality Management, Issues and Economic Implications. 2012; 1–40.

447.

Creamer NG, Dunning RD. Local food systems for a healthy population. North Carolina medical journal. 2012;73: 310–314.

448.

Bevier G. Global food systems: Feeding the world. Reproduction in Domestic Animals. 2012;47: 77–79. doi:[10.1111/j.1439-0531.2012.02058.x](https://doi.org/10.1111/j.1439-0531.2012.02058.x)

449.

Alkon AH, Mares TM. Food sovereignty in US food movements: Radical visions and neoliberal constraints. Agriculture and Human Values. 2012;29: 347–359. doi:[10.1007/s10460-012-9356-z](https://doi.org/10.1007/s10460-012-9356-z)

450.

Zasada I. Multifunctional peri-urban agriculture-A review of societal demands and the provision of goods and services by farming. LAND USE POLICY. 2011;28: 639–648. doi:[10.1016/j.landusepol.2011.01.008](https://doi.org/10.1016/j.landusepol.2011.01.008)

451.

Tregear A. Progressing knowledge in alternative and local food networks: Critical reflections and a research agenda. JOURNAL OF RURAL STUDIES. 2011;27: 419–430. doi:[10.1016/j.jrurstud.2011.06.003](https://doi.org/10.1016/j.jrurstud.2011.06.003)

452.

Bloom JD, Hinrichs CC. Moving local food through conventional food system infrastructure: Value chain framework comparisons and insights. RENEWABLE AGRICULTURE AND FOOD SYSTEMS. 2011;26: 13–23. doi:[10.1017/S1742170510000384](https://doi.org/10.1017/S1742170510000384)

453.

Holben DH. Field notes: People, Programs, & Policies: Development of an organic gardening workshop and community engagement activities to develop a healthy, local food system. Journal of Hunger and Environmental Nutrition. 2011;6: 233–235. doi:[10.1080/19320248.2011.576596](https://doi.org/10.1080/19320248.2011.576596)

454.

Gross J. Constructing a community food economy. Food and Foodways. 2011;19: 181–200. doi:[10.1080/07409710.2011.599775](https://doi.org/10.1080/07409710.2011.599775)

455.

Dunne JB, Chambers KJ, Giombolini KJ, Schlegel SA. What does local mean in the grocery store? Multiplicity in food retailers’ perspectives on sourcing and marketing local foods. Renewable Agriculture and Food Systems. 2011;26: 46–59. doi:[10.1017/S1742170510000402](https://doi.org/10.1017/S1742170510000402)

456.

De La Salle J. Local food hubs. Plan Canada. 2011;51: 33–37.

457.

Morris C, Kirwan J. Food commodities, geographical knowledges and the reconnection of production and consumption: The case of naturally embedded food products. GEOFORUM. 2010;41: 131–143. doi:[10.1016/j.geoforum.2009.09.004](https://doi.org/10.1016/j.geoforum.2009.09.004)

458.

Horne JE. Ensuring a sustainable, enduring agriculture. Agricultural and Resource Economics Review. 2010;39: 396–398. doi:[10.1017/S1068280500007395](https://doi.org/10.1017/S1068280500007395)

459.

Inwood SM, Sharp JS, Moore RH, Stinner DH. Restaurants, chefs and local foods: insights drawn from application of a diffusion of innovation framework. AGRICULTURE AND HUMAN VALUES. 2009;26: 177–191. doi:[10.1007/s10460-008-9165-6](https://doi.org/10.1007/s10460-008-9165-6)

460.

Follett JR. Choosing a Food Future: Differentiating Among Alternative Food Options. JOURNAL OF AGRICULTURAL & ENVIRONMENTAL ETHICS. 2009;22: 31–51. doi:[10.1007/s10806-008-9125-6](https://doi.org/10.1007/s10806-008-9125-6)

461.

Sherriff G. Towards healthy local food: Issues in achieving Just Sustainability. Local Environment. 2009;14: 73–92. doi:[10.1080/13549830802522566](https://doi.org/10.1080/13549830802522566)

462.

Parkins W, Craig G. Culture and the politics of alternative food networks. Food, Culture and Society. 2009;12: 77–103. doi:[10.2752/155280109X368679](https://doi.org/10.2752/155280109X368679)

463.

Goodman D, Goodman MK. Food Networks, Alternative. International Encyclopedia of Human Geography. 2009; 208–220. doi:[10.1016/B978-008044910-4.00889-0](https://doi.org/10.1016/B978-008044910-4.00889-0)

464.

Cross P, Edwards RT, Opondo M, Nyeko P, Edwards-Jones G. Does farm worker health vary between localised and globalised food supply systems? Environment International. 2009;35: 1004–1014. doi:[10.1016/j.envint.2009.04.009](https://doi.org/10.1016/j.envint.2009.04.009)

465.

Chalmers L, Joseph AE, Smithers J. Seeing farmers’ markets: Theoretical and media perspectives on new sites of exchange in New Zealand. Geographical Research. 2009;47: 320–330. doi:[10.1111/j.1745-5871.2009.00572.x](https://doi.org/10.1111/j.1745-5871.2009.00572.x)

466.

Alkon AH, Norgaard KM. Breaking the food Chains: An investigation of food justice activism. Sociological Inquiry. 2009;79: 289–305. doi:[10.1111/j.1475-682X.2009.00291.x](https://doi.org/10.1111/j.1475-682X.2009.00291.x)

467.

Anderson MD. Rights-based food systems and the goals of food systems reform. AGRICULTURE AND HUMAN VALUES. 2008;25: 593–608. doi:[10.1007/s10460-008-9151-z](https://doi.org/10.1007/s10460-008-9151-z)

468.

Smith BG. Developing sustainable food supply chains. PHILOSOPHICAL TRANSACTIONS OF THE ROYAL SOCIETY B-BIOLOGICAL SCIENCES. 2008;363: 849–861. doi:[10.1098/rstb.2007.2187](https://doi.org/10.1098/rstb.2007.2187)

469.

MacIas T. Working toward a just, equitable, and local food system: The social impact of community-based agriculture. Social Science Quarterly. 2008;89: 1086–1101. doi:[10.1111/j.1540-6237.2008.00566.x](https://doi.org/10.1111/j.1540-6237.2008.00566.x)

470.

Kinder CA. Connecting local food systems to youth. Journal of Extension. 2008;46. Available: <https://www.scopus.com/inward/record.uri?eid=2-s2.0-40249102268&partnerID=40&md5=656d343e58350de659ad56c192c4e4c7>

471.

Helmfrid H, Haden A, Ljung M. The role of Action Research (AR) in environmental research: Learning from a local organic food and farming research project. Systemic Practice and Action Research. 2008;21: 105–131. doi:[10.1007/s11213-007-9088-y](https://doi.org/10.1007/s11213-007-9088-y)

472.

Cox R, Holloway L, Venn L, Dowler L, Hein JR, Kneafsey M, et al. Common ground? Motivations for participation in a community-supported agriculture scheme. Local Environment. 2008;13: 203–218. doi:[10.1080/13549830701669153](https://doi.org/10.1080/13549830701669153)

473.

Alkon A. Paradise or pavement: The social constructions of the environment in two urban farmers’ markets and their implications for environmental justice and sustainability. Local Environment. 2008;13: 271–289. doi:[10.1080/13549830701669039](https://doi.org/10.1080/13549830701669039)

474.

Trauger A. Un/re-constructing the agrarian dream: Going back-to-the-land with an organic marketing co-operative in south-central Pennsylvania, USA. TIJDSCHRIFT VOOR ECONOMISCHE EN SOCIALE GEOGRAFIE. 2007;98: 9–20. doi:[10.1111/j.1467-9663.2007.00372.x](https://doi.org/10.1111/j.1467-9663.2007.00372.x)

475.

Dixon J, Omwega AM, Friel S, Burns C, Donati K, Carlisle R. The health equity dimensions of urban food systems. Journal of Urban Health. 2007;84: i118–i129. doi:[10.1007/s11524-007-9176-4](https://doi.org/10.1007/s11524-007-9176-4)

476.

Holloway L, Cox R, Venn L, Kneafsey M, Dowler E, Tuomainen H. Managing sustainable farmed landscape through `alternative’ food networks: a case study from Italy. GEOGRAPHICAL JOURNAL. 2006;172: 219–229. doi:[10.1111/j.1475-4959.2006.00205.x](https://doi.org/10.1111/j.1475-4959.2006.00205.x)

477.

Scholten BA. Firefighters in the UK and the US: Risk perception of local and organic foods. SCOTTISH GEOGRAPHICAL JOURNAL. 2006;122: 130–148. doi:[10.1080/00369220600917453](https://doi.org/10.1080/00369220600917453)

478.

Ross N. How civic is it? Success stories in locally focused agriculture in Maine. RENEWABLE AGRICULTURE AND FOOD SYSTEMS. 2006;21: 114–123. doi:[10.1079/RAF2005134](https://doi.org/10.1079/RAF2005134)

479.

Ilbery B, Maye D. Alternative (shorter) food supply chains and specialist livestock products in the Scottish-English borders. ENVIRONMENT AND PLANNING A-ECONOMY AND SPACE. 2005;37: 823–844. doi:[10.1068/a3717](https://doi.org/10.1068/a3717)

480.

Shreck A. Resistance, redistribution, and power in the Fair Trade banana initiative. AGRICULTURE AND HUMAN VALUES. 2005;22: 17–29. doi:[10.1007/s10460-004-7227-y](https://doi.org/10.1007/s10460-004-7227-y)

481.

DuPuis EM, Goodman D. Should we go “home” to eat?: Toward a reflexive politics of localism. Journal of Rural Studies. 2005;21: 359–371. doi:[10.1016/j.jrurstud.2005.05.011](https://doi.org/10.1016/j.jrurstud.2005.05.011)

482.

Combs Jr. GF, Hassan N. The Chakaria food system study: Household-level, case-control study to identify risk factor for rickets in Bangladesh. European Journal of Clinical Nutrition. 2005;59: 1291–1301. doi:[10.1038/sj.ejcn.1602242](https://doi.org/10.1038/sj.ejcn.1602242)

483.

Buttel F. Ever since Hightower: The politics of agricultural research activism in the molecular age. AGRICULTURE AND HUMAN VALUES. 2005;22: 275–283. doi:[10.1007/s10460-005-6043-3](https://doi.org/10.1007/s10460-005-6043-3)

484.

Feagan R, Morris D, Krug K. Niagara region farmers’ markets: Local food systems and sustainability considerations. Local Environment. 2004;9: 235–254. doi:[10.1080/1354983042000219351](https://doi.org/10.1080/1354983042000219351)

485.

Baker LE. Tending cultural landscapes and food citizenship in Toronto’s community gardens. Geographical Review. 2004;94: 305–325. doi:[10.1111/j.1931-0846.2004.tb00175.x](https://doi.org/10.1111/j.1931-0846.2004.tb00175.x)

486.

Weatherell C, Tregear A, Allinson J. In search of the concerned consumer: UK public perceptions of food, farming and buying local. Journal of Rural Studies. 2003;19: 233–244. doi:[10.1016/S0743-0167(02)00083-9](https://doi.org/10.1016/S0743-0167(02)00083-9)

487.

Burke MJ. Biotechnology in agriculture and food systems: An example of science and society at a crossroad. Emirates Journal of Food and Agriculture. 2003;15: 17–28. doi:[10.9755/ejfa.v15i2.5003](https://doi.org/10.9755/ejfa.v15i2.5003)

488.

Hinrichs C, Kremer KS. Social inclusion in a midwest local food system project. Journal of Poverty. 2002;6: 65–90. doi:[10.1300/J134v06n01_04](https://doi.org/10.1300/J134v06n01_04)

489.

Otzen U, Neubert S. Only swift agrarian development will create poverty-reducing economic dynamics in Africa. Quarterly Journal of International Agriculture. 2001;40: 295–299.

490.

Marsden T. Food matters and the matter of food: Towards a new food governance? SOCIOLOGIA RURALIS. 2000;40: 20+. doi:[10.1111/1467-9523.00129](https://doi.org/10.1111/1467-9523.00129)

491.

Lacy WB. Empowering communities through public work, science, and local food systems: Revisiting democracy and globalization. Rural Sociology. 2000;65: 3–26. doi:[10.1111/j.1549-0831.2000.tb00340.x](https://doi.org/10.1111/j.1549-0831.2000.tb00340.x)

492.

Pottier J. Village Responses to Food Marketing Alternatives in Northern Zambia: The Case of the Mambwe Economy. IDS Bulletin. 1986;17: 51–56. doi:[10.1111/j.1759-5436.1986.mp17001009.x](https://doi.org/10.1111/j.1759-5436.1986.mp17001009.x)

493.

Gephart M. African States and Agriculture: Issues for Research. IDS Bulletin. 1986;17: 57–63. doi:[10.1111/j.1759-5436.1986.mp17001010.x](https://doi.org/10.1111/j.1759-5436.1986.mp17001010.x)

494.

Mysids in the marine economy. Advances in Marine Biology. 1980;18: 258–263. doi:[10.1016/S0065-2881(08)60367-3](https://doi.org/10.1016/S0065-2881(08)60367-3)
