# Supplementary material for: The work of farmers in short food supply chains: Systematic literature review and research agenda
Source: PLoS One. 2025 Jun 4;20(6):e0314175. doi: 10.1371/journal.pone.0314175 (PMC12136416; doi:10.1371/journal.pone.0314175)
Supplement: Attachment — Submitted filename: Response to reviewers.docx [file pone.0314175.s010.docx]

**Response to reviewers**

Dear Editor and Reviewers,

We sincerely appreciate your insightful comments and valuable suggestions regarding our manuscript. In response to your recommendations, we have undertaken a substantial update of our Systematic Literature Review. This process has resulted in the incorporation of 23 additional documents into our corpus, expanding the total number of analyzed articles from 56 to 79. These new references are cataloged in the table at the end of this document. This expansion has significantly enriched our review's findings while maintaining the coherence of our primary conclusions. Consequently, the results sections have been substantially enhanced. Furthermore, we have bolstered the introduction with key references from our field of research.

In an effort to improve readability, we have made considerable strides in increasing conciseness, including reducing the overall manuscript length and shortening section titles. We have also ensured strict adherence to the methodological requirements specific to Systematic Literature Reviews. We trust that these modifications meet your expectations and elevate the overall quality of our manuscript.

Below, we provide point-by-point responses to your specific remarks. We would like to point out that, unlike our article, this document has not been reviewed by an English translator.

We look forward to your feedback on these revisions,

Philippine Dupé, Pierre Gasselin, Benoît Dedieu and Guillaume Ollivier.

# Editor comments

1. *I also noticed that the authors have cited only one paper published in 2024. Given the availability of numerous relevant papers on this topic, I recommend including more papers available for 2024 and beyond.*

In accordance with your recommendations, we have substantially updated our Systematic Literature Review. 23 additional documents have been incorporated, increasing the total number of analyzed articles from 56 to 79. 9 of these new references were obtained through an updated query, including articles published between 01/01/2024 and 31/08/2024. 14 articles were subsequently selected via our "citation chasing" stage, utilizing a corpus of cited references and those citing our corpus. Indeed, this corpus was itself updated through our revised query. All new references are listed in the table at the end of this document. This addition has considerably enriched the results of our review while maintaining the consistency of our main conclusions. The various results sections have been extensively reworked to incorporate these new findings.

2. *Both reviewers have suggested, and I agree, that the headings and sub-headings should be streamlined to better align with the context and adhere to standard conventions. Additionally, the manuscript in its current form is lengthy, with too many subheadings. Condensing the content and reducing the number of subheadings would improve readability and focus. Hence with the help of provided sample copy, please reduce it.*

We have undertaken a significant effort to improve concision while maintaining the integrity and richness of our analysis. The manuscript has been reduced by approximately 6,000 characters (including spaces), equivalent to nearly 1,000 words. The final text, from abstract to conclusion, now comprises approximately 12,000 words. This revised length is in line with the standards for literature reviews in our research field. Three examples of comparable reviews are provided below for reference. We believe this revision strikes a balance between comprehensive analysis and concise presentation, adhering to the expectations of our field while addressing the need for brevity.

1.	Martin T, Gasselin P, Hostiou N, Feron G, Laurens L, Purseigle F, et al. Robots and transformations of work in farm: a systematic review of the literature and a research agenda. Agron Sustain Dev. 2022;42. doi:10.1007/s13593-022-00796-2

2.	O’Connor G, Reis K, Desha C, Burkett I. Valuing farmers in transitions to more sustainable food systems: A systematic literature review of local food producers’ experiences and contributions in short food supply chains. Agric Hum Values. 2024 [cited 30 Aug 2024]. doi:10.1007/s10460-024-10601-3

3.	Csordas A, Lengyel P, Fuzesi I. Who Prefers Regional Products? A Systematic Literature Review of Consumer Characteristics and Attitudes in Short Food Supply Chains. Sustainability. 2022;14: 8990. doi:10.3390/su14158990

We faced two significant imperatives that limited our ability to reduce the manuscript’s length more than we did. Indeed, the incorporation of 23 new articles into our results has required additional content and analysis. In addition, to ensure our work qualifies as a Systematic Literature Review, we needed to include several methodological elements as required by the PRISMA method (S5 Table). However, we have removed two non-essential figures (relocated to S4 Table and Figs) for greater conciseness.

In response to your recommendations, we have shortened our headings, now focusing on key words to enhance readability. This revision was inspired by the exemplar articles you suggested. Finally, we propose to maintain a three-levels heading structure, which appears to be permitted by the journal. This structure enhances reading fluidity, by allowing for shorter paragraphs and more clearly signaling the nature of information addressed in each section.

# Comments from Reviewer #2

1. *The paper would be more attractive if it developed a theoretical model to make the Short food supply chain more effective.*

The primary objective of our Systematic Literature review is to provide a rigorous and comprehensive assessment of the available scientific knowledge on the topic of farmers' work in SFSCs. To achieve this goal, we have adopted a transparent and reproducible methodology, detailed in a research protocol (S1 Document). Based on our findings, we also propose a research agenda in our discussion section. Our choice to conduct a Systematic Literature Review does not appear to be compatible with developing a theoretical model to improve SFSCs efficiency. However, our results could serve as a foundation for future work on such models. In our conclusion, we emphasize the need for increased research on farmers' work in SFSCs.

2. *Besides, it will contribute significantly if some policy review in the global south and north context is incorporated.*

Conducting such a review appears crucial to enhance the effectiveness of SFSCs. However, in this article, we chose to focus on scientific knowledge. Nevertheless, we call for our findings to be taken into account when designing public policies around SFSCs in our conclusion, and emphasize the importance of strengthening our understanding of farmers' work in SFSCs with various methodologies.

3. *Besides, the research implications are another important part that needs to be addressed.*

Our discussion section (§9) proposes a research agenda that details all the "knowledge gaps" identified across the various dimensions of work in our analytical grid. We specifically highlight the less explored sub-themes and the less frequently used methodologies.

4. *Moreover, the papers seem very lengthy; if possible, it is better to reduce the size.*

Cf Response to editor’s comments 2

# Comments from Reviewer #1

1. *The language and grammar are NOT of an acceptable standard. The structure and layout of the paper is not logical. Acronyms & Abbreviations sections given in the paper need to be deleted and each of these acronyms need to be used inside the paper. The tables need to be short wherever possible only.*

Our article has been translated by an experienced English-speaking translator who is accustomed to collaborate with researchers in our field. The currently revised version has been proofread by the same translator.

Our paper is composed of:

- a contextual introduction (§1);
- a methods section (§2), detailing the various steps of the Systematic Literature Review methodology, on the basis of the PRISMA checklist (S5 Table);
- six results sections (§3-8), corresponding to the five analytical prisms of our research question and to a bibliometric analysis of the papers included in our review;
- a discussion section (§9), proposing a detailed research agenda;
- a conclusion.

To enhance readability, we have significantly reduced the length of our titles, now focusing on key words to enhance readability.

Acronyms and abbreviations are explained within the paper.

We have included only the essential tables for our article to be recognized as a Systematic Literature Review and have moved two figures to the supporting information (S4 Document).

2. *Introduction section should be supported by important pieces of literature on the issues discussed. All of the challenges discussed are fine but need to be summarized.*

We have added five references on our introduction to strengthen our presentation of the key issues, while also reducing the overall length of the introduction for greater conciseness. Four of these references are widely cited within our corpus (S3 Table).

- Renting H, Marsden TK, Banks J. Understanding Alternative Food Networks: Exploring the Role of Short Food Supply Chains in Rural Development. Environ Plan Econ Space. 2003;35: 393–411. doi:10.1068/a3510
- Forssell S, Lankoski L. The sustainability promise of alternative food networks: an examination through “alternative” characteristics. Agric Hum Values. 2015;32: 63–75. doi:10.1007/s10460-014-9516-4
- O’Connor G, Reis K, Desha C, Burkett I. Valuing farmers in transitions to more sustainable food systems: A systematic literature review of local food producers’ experiences and contributions in short food supply chains. Agric Hum Values. 2024 [cited 30 Aug 2024]. doi:10.1007/s10460-024-10601-3
- Allen P, FitzSimmons M, Goodman M, Warner K. Shifting plates in the agrifood landscape: the tectonics of alternative agrifood initiatives in California. J Rural Stud. 2003;19: 61–75. doi:10.1016/S0743-0167(02)00047-5
- Marsden T, Banks J, Bristow G. Food Supply Chain Approaches: Exploring their Role in Rural Development. Sociol Rural. 2000;40: 424–438. doi:10.1111/1467-9523.00158

3. *The methodology adopted for analyzing all these is not clear and needs to be written in detail as a section on what methodology and approach followed in the whole paper, why, and how done*

We have adopted a Systematic Literature Review methodology as outlined by Munn et al. (2018). In paragraphs §2.1, 2.2, 2.3, 2.4, and 2.5, we have detailed the various steps of this approach in accordance with the requirements of the PRISMA checklist (see Table S5). A detailed protocol, made public in October 2023, further specifies our choices and is included in the supporting information (S1 Document). It is referenced in the main text. To improve clarity, we have reduced the length of the methodology section and merged two paragraphs.

4. *Review of literature section: The paper only reviewed major policy documents that were published on this topic until now. The coverage of the literature is limited need to be discussed the main findings of key papers. Therefore, from the perspective of a literature review, the paper has some obvious shortages too, which should be overcome. At the end of the review of the literature section, it would be nice to have a small summary paragraph or two-three sentences, that may have discussed the fundamental linkage of these papers. For example, what mechanisms are emphasized in each or overall paper? How are those older and newer papers linked to each other, summarized? After all, by reading the review someone would expect a clear “summary line" that helps to understand the connection and contribution of these papers. This is the first thing that should be improved in the paper, so that will be clear to the reader.*

The primary objective of our systematic literature review is to provide a rigorous and comprehensive assessment of the available scientific knowledge on the topic of farmers' work in SFSCs. In our review, we have considered peer-reviewed articles, book chapters, or scientific reviews indexed in Web of Science and Scopus that meet our selection criteria, as detailed in our PICO table (Table 2). 79 articles were selected following careful examination of 920 articles (Fig 1) retrieved from a detailed search query (which is described in Table 1) and a citation chasing stage (§2.3). The selection process is detailed in section 2.3.

To complete our corpus, we have updated our review here, adding approximately 23 documents to our studied collection.

Each results section begins with an introduction presenting the nature of the various articles mobilized. This introduction serves to establish connections between these articles (methods, topics addressed).

The lack of information on certain topics is also a result of the review, which thus proposes a research agenda (§9). This agenda specifically highlights the less explored sub-themes and the less frequently used methodologies.

5. *The article is a little deficient in providing insights on policy and practices in other countries as well, that have adopted different policy challenges.*

Public policies are one element of results among others concerning farmers' work in SFSCs. Information on public policies impacting work of farmers marketing via SFSCs has been collected and summarized in the results sections §4.3 and §6.2.3. It is particularly noteworthy that very few papers dealing with work in SFSCs examine the dimensions of public policies related to this work. We have therefore identified this knowledge gap and call for strengthening research on this topic (§9.1, lines 779-780 and §9.2, lines 802-803).

6. *What specific policies need to be taken to be integrated? There has been quite a large amount of work done in this area both theoretical and empirical from different countries.*

In this review, we have chosen to focus on assessing the available scientific knowledge and proposing a research agenda. The collected articles present few results on public policies related to work in SFSCs. Therefore, we cannot propose a public policy agenda based on this corpus. However, we are convinced that our results could be used to design public policies around SFSCs. We believe that a more in-depth analysis of existing public policies could be the subject of another paper, employing different methodologies (databases, queries) than those used in this article.

7. *The paper is though supported by theoretical backdrops. It would have been more useful had the author discussed other countries' experiences before bringing in other experiences. Again, a literature review of the policy and practice aspects of other countries of the world would provide a comparative perspective to the study and add value to the study.*

In the introduction, we draw on references from various geographical contexts. In the results section §3.2, we emphasize that “*The study of agricultural work in SFSCs, with SFSCs designated as such, is mostly carried out by Western researchers focusing on Western contexts.*” Only 14 out of 79 publications come from South America, Africa, or Asia, even though marketing via SFSCs takes place in all countries of the world. This is therefore a significant finding. We thus call for strengthening case studies in other territories to better inform current practices in other locations.

8. *The paper could have discussed themes using ideas from existing literature which is missing like, what is the role of Govt interventions in this case? Possibly, a thorough literature review on the theoretical aspects would be useful and could add to the scholastic value of the article.*

Like other literature reviews, we have chosen to focus the discussion on developing a research agenda, rather than discussing new ideas by mobilizing other existing literature. We have been constrained by the already substantial size of the article and by the imperative to update the review. The selection of 23 new articles from 237 new references, as well as their analysis and integration into our results, indeed required considerable work, completed in just a month and a half.

The vast majority of articles in the corpus offer empirical results on our topic: our results therefore focus on these aspects rather than on theoretical aspects.

9. *As per the aim of the journal, at least to some extent author may like to add in review section relating to specific issues.*

Our article is composed of six results sections, dealing with specific issues linked to the topic of farmers' work in SFSFs. The choice of these sub-themes is based on our analysis grid. We have constructed this grid to cover the main dimensions of work present in the literature, by mobilizing a body of literature on work, gathered on the basis of our research expertise in this area. We feel that this grid covers our theme satisfactorily. We are also constrained by the current size of the manuscript.

10. *Most of the references cited are from different policy documents of national and international agencies, which need to be supported by adding a strong review of literature from journals.*

*Suggested reading and to be cited:*

*Follow FAO Rome, IRRI Philippines some study*

*Devkota, N., Joshi, A., Khanal, G., Mahapatra, S.K., Gautam, N., Paudel, U.R. and Bhandari, U. (2023), "Awareness on agricultural entrepreneurship among youth farmers: an empirical study from Western Nepal", Journal of Agribusiness in Developing and Emerging Economies, Vol.13, No.5, 21st November, pp. 812-830,*

In our review, we have considered 79 peer-reviewed articles, book chapters, or scientific reviews indexed in Web of Science and Scopus and retrieved from a detailed search query (Table 1). These articles were selected following careful examination of 920 articles (Fig 1) thanks to a selection process detailed in section 2.3. We cannot include in results from articles not originating from our query or our citation chasing stage in our results sections.

# Journal Requirement

*1. When submitting your revision, we need you to address these additional requirements.*

*Please ensure that your manuscript meets PLOS ONE's style requirements, including those for file naming. The PLOS ONE style templates can be found at 
https://journals.plos.org/plosone/s/file?id=wjVg/PLOSOne_formatting_sample_main_body.pdf and* 
[*https://journals.plos.org/plosone/s/file?id=ba62/PLOSOne_formatting_sample_title_authors_affiliations.pdf*](https://journals.plos.org/plosone/s/file?id=ba62/PLOSOne_formatting_sample_title_authors_affiliations.pdf)

Done.

*2. Thank you for stating in your Funding Statement: This work was financially supported by the Fondation de France and by the metaprogram "Métabio" from the National Research Institute for Agriculture, Food and the Environment (INRAE, France), which financed a PhD grant for Philippine Dupé. The funders had no role in study design, data collection and analysis, decision to publish, or preparation of the manuscript.Please provide an amended statement that declares *all* the funding or sources of support (whether external or internal to your organization) received during this study, as detailed online in our guide for authors at http://journals.plos.org/plosone/s/submit-now.  Please also include the statement “There was no additional external funding received for this study.” in your updated Funding Statement. 
Please include your amended Funding Statement within your cover letter. We will change the online submission form on your behalf.*

Done.

*3. Please upload a new copy of Figures 2, 5, 6 and 7, as the detail is not clear. Please follow the link for more information: https://blogs.plos.org/plos/2019/06/looking-good-tips-for-creating-your-plos-figures-graphics/"* [*https://blogs.plos.org/plos/2019/06/looking-good-tips-for-creating-your-plos-figures-graphics/*](https://blogs.plos.org/plos/2019/06/looking-good-tips-for-creating-your-plos-figures-graphics/)*"*

Done.

*4. Please review your reference list to ensure that it is complete and correct. If you have cited papers that have been retracted, please include the rationale for doing so in the manuscript text, or remove these references and replace them with relevant current references. Any changes to the reference list should be mentioned in the rebuttal letter that accompanies your revised manuscript. If you need to cite a retracted article, indicate the article’s retracted status in the References list and also include a citation and full reference for the retraction notice.*

Done.

# New cited references and deleted references

| **Document type** | **Authors** | **Date** | **Title** | **Journal** | **Source if included in the corpus** | **§** |
| --- | --- | --- | --- | --- | --- | --- |
| Article | Renting H, Marsden TK, Banks J. | 2003 | Understanding Alternative Food Networks: Exploring the Role of Short Food Supply Chains in Rural Development. | Environ Plan Econ Space. |  | Introduction |
| Article | Forssell S, Lankoski L. | 2015 | The sustainability promise of alternative food networks: an examination through “alternative” characteristics. | Agric Hum Values. |  | Introduction |
| Article | Allen P, FitzSimmons M, Goodman M, Warner K. | 2003 | Shifting plates in the agrifood landscape: the tectonics of alternative agrifood initiatives in California. | J Rural Stud. |  | Introduction |
| Article | Marsden T, Banks J, Bristow G. | 2000 | Food Supply Chain Approaches: Exploring their Role in Rural Development. | Sociol Rural. |  | Introduction |
| Article | Black, JE | 2024 | Fun, community, and culture in a Japanese alternative food network | FRONTIERS IN SUSTAINABLE FOOD SYSTEMS | Query 2024 | Results |
| Article | Chavez-Miguel, G; Hämmerle, J; González, A; Canetti, C; Gleich, P; Halfast, RL; et al. | 2024 | Local food system resilience in the context of shocks and crises: vulnerabilities and responses of agroecology-based farmers in Peru, Germany, and the United States | AGROECOLOGY AND SUSTAINABLE FOOD SYSTEMS | Query 2024 | Results |
| Article | Flachs, A; Raturi, A; Low, M; Miller, V; Norton, J; Redmond, C; Thomas, H | 2024 | Digital tools for local farmers: Thinking with spreadsheets in the wake of the COVID-19 pandemic | CULTURE AGRICULTURE FOOD AND ENVIRONMENT | Query 2024 | Results |
| Article | Hvitsand, C; Nicolaysen, AM; Gjotterud, S; Raanaas, RK | 2024 | Piloting a co-created local and alternative food network involving professional buyers in Norway: Forces and tensions influencing viability | JOURNAL OF RURAL STUDIES | Query 2024 | Results |
| Article | O'Connor, G; Reis, K; Desha, C; Burkett, I | 2024 | Valuing farmers in transitions to more sustainable food systems: A systematic literature review of local food producers' experiences and contributions in short food supply chains | AGRICULTURE AND HUMAN VALUES | Query 2024 | Results |
| Article | Rossi, A; Piccoli, A; Feola, G | 2024 | Transforming labour around food? the experience of community supported agriculture in Italy | AGRICULTURE AND HUMAN VALUES | Query 2024 | Results |
| Article | Rosman, A; Macpherson, J; Arndt, M; Helming, K | 2024 | Perceived resilience of community supported agriculture in Germany | AGRICULTURAL SYSTEMS | Query 2024 | Results |
| Article | Kondo, C., Zollet, S., Kobayashi, M., Yamamoto, N. | 2024 | Fifty years of Teikei: the evolution of the movement’s ten principles and its impact on alternative food initiatives in Japan | Frontiers in Sustainable Food Systems | Query 2024 | Results |
| Article | Paraušić, V., Dashi, E.M., Subić, J., Pomianek, I., Šarić, B.B. | 2024 | Response of Short Food Supply Chains in Western Balkan countries to the covid crise : a case study in honey sector | European Countryside | Query 2024 | Results |
| Article | Galt R.E. ; Bradley K.A. ; Christensen L.; Van Soelen Kim J. ; Lobo R. | 2015 | Eroding the community in Community supported agriculture : competition's effects in alternative food networks in California | Sociologia Ruralis | Citation Chasing 2024 | Results |
| Article | Ilbery, B.; Maye, D. | 2005 | alternative (shorter) food supply chains and specialist livestock products in the scottish–english borders | Environment and Planning A Economy and Space | Citation Chasing 2024 | Results |
| Article | Hvitsand, C. | 2016 | community supported agriculture (csa) as a transformational act—distinct values and multiple motivations among farmers and consumers | Agroecology and Sustainable Food Systems | Citation Chasing 2024 | Results |
| Article | Balázs B.; Balázs B. ; Lazányi O. | 2016 | prospects for the future: community supported agriculture in hungary | Futures | Citation Chasing 2024 | Results |
| Article | Conner, D.; King, B.R. ; Kolodinsky, J. ; Roche, E. ; Koliba, C.; Trubek, A.B. | 2012 | you can know your school and feed it too: vermont farmers’ motivations and distribution practices in direct sales to school food services | Agriculture and Human Values | Citation Chasing 2024 | Results |
| Article | Malak-Rawlikowska A; Majewski E; Wąs A; Ole Borgen S; Csillag P; Donati M; et al; | 2019 | measuring the economic, environmental, and social sustainability of short food supply chains | Sustainability | Citation Chasing 2024 | Results |
| Article | Cox, R.; Holloway, L.; Venn, L.; Dowler, L. ; Ricketts, Hein J.; Kneafsey, M. ; Tuomainen, H. | 2008 | common ground? motivations for participation in a community-supported agriculture scheme | Local Environment | Citation Chasing 2024 | Results |
| Article | Charatsari C ; Kitsios F; Lioutas ED | 2019 | short food supply chains: the link between participation and farmers' competencies | Renewable Agriculture and Food Systems | Citation Chasing 2024 | Results |
| Article | Bui T A; Nguyen AH; Huong L T T; Nguyen V P; Hao L T T; Tran TTH et al. | 2021 | can a short food supply chain create sustainable benefits for small farmers in developing countries? an exploratory study of vietnam | Sustainability | Citation Chasing 2024 | Results |
| Article | Eckert Matzembacher E.; Bittencourt Meira F. | 2019 | sustainability as business strategy in community supported agriculture | British Food Journal | Citation Chasing 2024 | Results |
| Article | Dunay A; Lehota J; Mácsai E C; Bálint Illés C | 2018 | short supply chain: goals, objectives and attitudes of producers | Acta Polytechnica Hungarica | Citation Chasing 2024 | Results |
| Article | de Mansoldo M; Cavagnaro E; de Oliveira Menezes V | 2019 | farmers’ trade skills: exploring the local food chain in leeuwarden, the 2018 european capital of culture | Research in Hospitality Management | Citation Chasing 2024 | Results |
| Article | Savels R; Dessein J; Lucantoni D ; Speelman S | 2024 | assessing the agroecological performance and sustainability of community supported agriculture farms in flanders, belgium | Frontiers in Sustainable Food Systems | Citation Chasing 2024 | Results |
| Article | Wang M; Kumar V; Ruan X; Mülling Neutzling D | 2019 | farmers’ attitudes towards participation in short food supply chains: evidence from a chinese field research | Revista Ciências Administrativas | Citation Chasing 2024 | Results |
| Website | The Cochrane Collaborati | 2024 | Cochrane.org. |  |  | Methods |
| Website | Joanna Briggs Institute. | 2024 | Home Page \| JBI. |  |  | Methods |
| Report | Agreste | 2023 | Recensement Agricole 2020 : Près d’une exploitation sur quatre vend en circuit court. Agreste |  |  | Conclusion |
| Thesis | Chiaverina P. | 2024 | An analysis of the sustainability and dynamics of short food supply chains. |  |  | Conclusion |
