# Supplementary material for: The work of farmers in short food supply chains: Systematic literature review and research agenda
Source: PLoS One. 2025 Jun 4;20(6):e0314175. doi: 10.1371/journal.pone.0314175 (PMC12136416; doi:10.1371/journal.pone.0314175)
Supplement: Attachment — Submitted filename: Reviewer comments 16th August 2024.doc [file pone.0314175.s008.doc]

**Reviewer comments**

**Title: "** **The work of farmers in Short Food Supply Chains: Systematic Literature Review and research agenda"**

Manuscript Number: PONE-D-24-29719

**Comments to the Author/s**

The paper is well organized. The author/s has carried out a study on the work of farmers in Short Food Supply Chains: Systematic Literature Review (SLR) and research agenda, using SLR techniques.

**Two main reasons for recommendation are:**

1. The subject matter of this work is of relevance to the theme of the journal which would be helpful in the context of analysis of the overall literature contribution to farmers in Short Food Supply Chains. The information presented is original. The content of the submission is factual. The title and abstract accurately reflect the content of the submission.
2. The most insightful part is that the results of this study will be useful for the government to make policies related to agriculture and farmers in Short Food Supply Chains.

**Minor Revisions recommend**

1. The language and grammar are NOT of an acceptable standard. The structure and layout of the paper is not logical. Acronyms & Abbreviations sections given in the paper need to be deleted and each of these acronyms need to be used inside the paper. The tables need to be short wherever possible only.
2. Introduction section should be supported by important pieces of literature on the issues discussed. All of the challenges discussed are fine but need to be summarized.
3. The methodology adopted for analyzing all these is not clear and needs to be written in detail as a section on what methodology and approach followed in the whole paper, why, and how done
4. Review of literature section: The paper only reviewed major policy documents that were published on this topic until now. The coverage of the literature is limited need to be discussed the main findings of key papers. Therefore, from the perspective of a literature review, the paper has some obvious shortages too, which should be overcome. At the end of the review of the literature section, it would be nice to have a small summary paragraph or two-three sentences, that may have discussed the fundamental linkage of these papers. For example, what mechanisms are emphasized in each or overall paper? How are those older and newer papers linked to each other, summarized? After all, by reading the review someone would expect a clear “summary line" that helps to understand the connection and contribution of these papers. This is the first thing that should be improved in the paper, so that will be clear to the reader.
5. The article is a little deficient in providing insights on policy and practices in other countries as well, that have adopted different policy challenges.
6. What specific policies need to be taken to be integrated? There has been quite a large amount of work done in this area both theoretical and empirical from different countries.
7. The paper is though supported by theoretical backdrops. It would have been more useful had the author discussed other countries' experiences before bringing in other experiences. Again, a literature review of the policy and practice aspects of other countries of the world would provide a comparative perspective to the study and add value to the study.
8. The paper could have discussed themes using ideas from existing literature which is missing like, what is the role of Govt interventions in this case? **Possibly, a thorough literature review on the theoretical aspects** would be useful and could add to the scholastic value of the article.
9. As per the aim of the journal, at least to some extent author may like to add in review section relating to specific issues.
10. Most of the references cited are from different policy documents of national and international agencies, which need to be supported by **adding a strong review of literature** from journals.

***Suggested reading and to be cited:***

1) Follow FAO Rome, IRRI Philippines some study
2) Devkota, N., Joshi, A., Khanal, G., Mahapatra, S.K., Gautam, N., Paudel, U.R. and Bhandari, U. (2023), "Awareness on agricultural entrepreneurship among youth farmers: an empirical study from Western Nepal", ***Journal of Agribusiness in Developing and Emerging Economies***, Vol.13, No.5, 21^st^ November, pp. 812-830,

<https://www.emerald.com/insight/content/doi/10.1108/JADEE-06-2021-0150/full/html>, <https://doi.org/10.1108/JADEE-06-2021-0150>

Overall, the manuscript is not publishable in its current form. I recommend **MINOR REVISIONS** for publication in the Journal after the author/s addresses the above queries and suggestions given.
